# The novel antibiotic rhodomyrtone traps membrane proteins in vesicles with increased fluidity

**DOI:** 10.1371/journal.ppat.1006876

**Published:** 2018-02-16

**Authors:** Dennapa Saeloh, Varomyalin Tipmanee, Kin Ki Jim, Marien P. Dekker, Wilbert Bitter, Supayang P. Voravuthikunchai, Michaela Wenzel, Leendert W. Hamoen

**Affiliations:** 1 Excellence Research Laboratory on Natural Products, Faculty of Science and Natural Product Research Center of Excellence, Prince of Songkla University, Hat Yai, Songkhla, Thailand; 2 Department of Microbiology, Faculty of Science, Prince of Songkla University, Hat Yai, Songkhla, Thailand; 3 Department of Biomedical Sciences, Faculty of Medicine, Prince of Songkla University, Hat Yai, Songkhla, Thailand; 4 Department of Medical Microbiology and Infection Control, VU University Medical Center, Amsterdam, The Netherlands; 5 Department of Clinical Genetics, Center for Neurogenomics and Cognitive Research (CNCR), Neuroscience Amsterdam, VU University Medical Center, Amsterdam, The Netherlands; 6 Department of Molecular Cell Biology, Amsterdam Institute for Molecules, Medicines and Systems, Faculty of Science, Vrije Universiteit Amsterdam, Amsterdam, The Netherlands; 7 Bacterial Cell Biology, Swammerdam Institute for Life Sciences, University of Amsterdam, Amsterdam, The Netherlands; Nanyang Technological University, SINGAPORE

## Abstract

The acylphloroglucinol rhodomyrtone is a promising new antibiotic isolated from the rose myrtle *Rhodomyrtus tomentosa*, a plant used in Asian traditional medicine. While many studies have demonstrated its antibacterial potential in a variety of clinical applications, very little is known about the mechanism of action of rhodomyrtone. Preceding studies have been focused on intracellular targets, but no specific intracellular protein could be confirmed as main target. Using live cell, high-resolution, and electron microscopy we demonstrate that rhodomyrtone causes large membrane invaginations with a dramatic increase in fluidity, which attract a broad range of membrane proteins. Invaginations then form intracellular vesicles, thereby trapping these proteins. Aberrant protein localization impairs several cellular functions, including the respiratory chain and the ATP synthase complex. Being uncharged and devoid of a particular amphipathic structure, rhodomyrtone did not seem to be a typical membrane-inserting molecule. In fact, molecular dynamics simulations showed that instead of inserting into the bilayer, rhodomyrtone transiently binds to phospholipid head groups and causes distortion of lipid packing, providing explanations for membrane fluidization and induction of membrane curvature. Both its transient binding mode and its ability to form protein-trapping membrane vesicles are unique, making it an attractive new antibiotic candidate with a novel mechanism of action.

## Author summary

Bacterial antibiotic resistance constitutes a major public healthcare issue and deaths caused by antimicrobial resistance are expected to soon exceed the number of cancer-related fatalities. In order to fight resistance, new antibiotics have to be developed that are not affected by existing microbial resistance strategies. Thus, antibiotics with novel or multiple targets are urgently needed. Rhodomyrtone displays excellent antibacterial activity, has been safely used in traditional Asian medicine for a long time, and resistance against this promising antibiotic candidate could not be detected in multiple passaging experiments. Here we demonstrate that rhodomyrtone possesses a completely novel mechanism of action, which is opposed to that of existing cell envelope-targeting drugs, minimizing the risk of cross-resistance, and in fact rhodomyrtone is highly active against e.g. vancomycin-resistant *Staphylococcus aureus*. Thus, rhodomyrtone is an extremely interesting compound for further antibacterial drug development.

## Introduction

The vast majority of antibiotics currently used in the clinic are derived from microbial sources [1]. However, plants represent an enormous source of potent bioactive molecules and several plant-derived compounds show promising antibacterial activities [2]. Considering the alarming rise in antibiotic-resistant pathogens, it is crucial to explore the potential of antimicrobials from herbal sources for new antibiotic development [3,4]. One such promising compound is the acylphloroglucinol rhodomyrtone, isolated from the leaves of the rose myrtle *Rhodomyrtus tomentosa* [5]. Rhodomyrtone is highly active against a broad range of Gram-positive bacteria, among which *Bacillus*, *Enterococcus*, *Staphylococcus*, and *Streptococcus* species, including clinical isolates and multi-resistant strains [5,6], and eradicates mature biofilms of *Propionibacterium acnes* [7], *Staphylococcus aureus*, and *Staphylococcus epidermidis* [8]. Rhodomyrtone is bactericidal and its minimal inhibitory concentrations are comparable to that of the last-resort antibiotics vancomycin and daptomycin [8]. Attempts to cultivate resistant mutants in the laboratory have not been successful in multiple passaging experiments [6]. Importantly, no cytotoxic effects have been observed on human fibroblasts and erythrocytes [6,9]. Rhodomyrtone did also not cause skin irritation upon topical application in rabbits [10] and acute toxicity tests did not show adverse effects in mice, when injected [11]. Furthermore, it has been proven effective against *Propionibacterium acne* biofilms [10,12], showed excellent results in preventing *staphylococcal* adhesion and invasion in a tissue model of bovine mastitis [13], and prevented adhesion of dental pathogens to plastic surfaces and human buccal cells [14].

Despite being such a promising new antibiotic candidate, the mechanism by which rhodomyrtone kills bacteria is not yet understood. Early studies with *Streptococcus mutans*, *Streptococcus pyogenes*, and *S*. *aureus* reported no distinct cell lysis or leakage of intracellular content, but upregulation of core metabolic pathways [5,6,15]. These results prompted the conclusion that rhodomyrtone likely has an intracellular target. A computational docking approach identified the dihydrofolate reductase DfrA as potential target but this could not be experimentally confirmed [16]. The same study also found a possible interaction of rhodomyrtone with the essential cell division protein FtsZ, and an earlier proteomic study showed reduced FtsZ levels and changes in cell size, shape, and septum formation in rhodomyrtone-treated *S*. *aureus* [17]. However, a recent study demonstrated that rhodomyrtone is unable to specifically inhibit *B*. *subtilis* FtsZ polymerization *in vivo* but rather affects several different cell division proteins [18].

Because of these conflicting observations, we investigated the effect of rhodomyrtone on the bacterial cell envelope more closely using a recently established cell biology-based approach to study the mode of action of antibacterial compounds [19,20]. Using fluorescence light and high-resolution microscopy, together with specialized membrane dyes, *in vitro* techniques, and molecular modeling, we found that rhodomyrtone primarily acts on the cell membrane, but in an unexpected manner. Rhodomyrtone induces dramatic membrane invaginations resulting in intracellular vesicles that attract and trap a variety of membrane proteins. These structures also attract flexible membrane lipids leading to highly fluid (liquid-disordered) membrane domains and subsequent rigidification (liquid-ordered) of the rest of the membrane, resulting in further delocalization of peripheral membrane proteins. These membrane distortions impair multiple essential membrane-associated processes, including respiration and ATP synthesis. Molecular dynamics simulations suggested that rhodomyrtone accomplishes membrane remodeling by transiently interacting with phospholipid head groups, thus without integrating into the lipid bilayer. Such a molecular mechanism has not been observed for any membrane-targeting antimicrobial before and explains why rhodomyrtone has long been thought not to target the cell envelope.

## Results

### Bacterial cytological profiling

**Fig 1:**
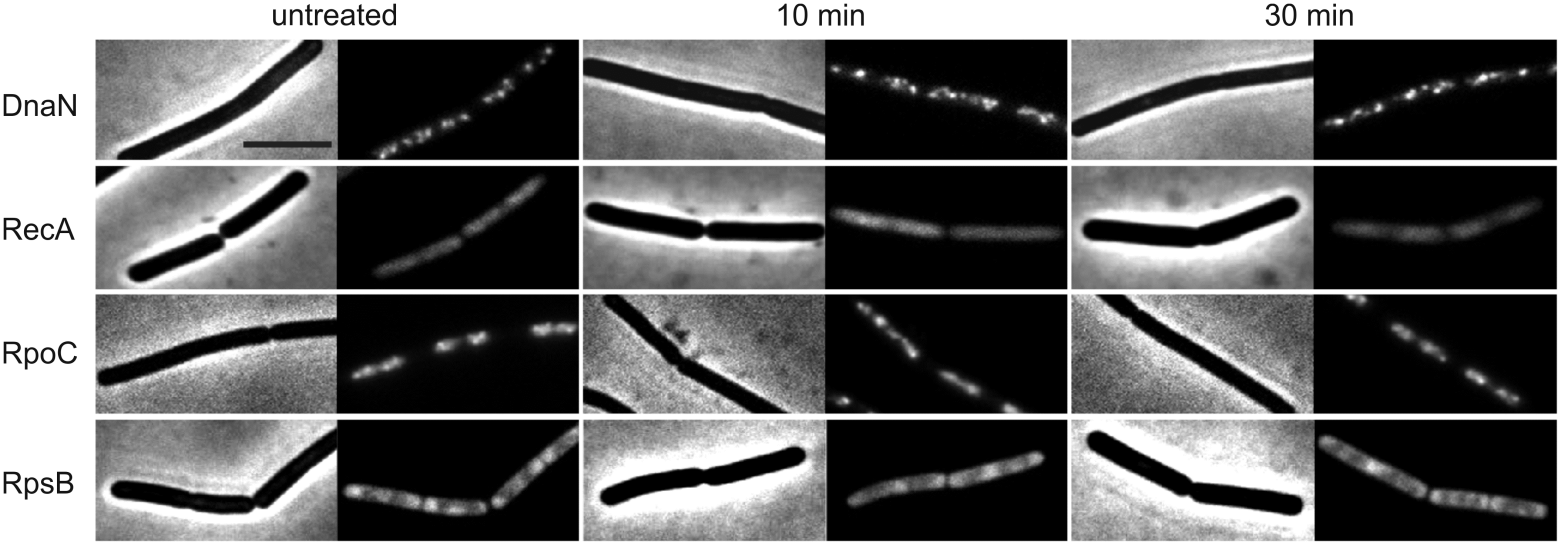
Localization of intracellular reporter proteins. *B*. *subtilis* strain HM771 (*dnaN-gfp*, reporter for impaired replication), strain UG-10 (*recA-gfp*, reporter for DNA damage), strain 1048 (*rpoC-gfp*, reporter for impaired RNA synthesis), and strain 1049 (*rpsB-gfp*, reporter for impaired protein synthesis) were grown until an OD_600_ of 0.3 and subsequently treated with 1x MIC of rhodomyrtone. Scale bar 2 μm.

Previous studies have suggested that rhodomyrtone could inhibit intracellular processes [5,16,21]. To investigate this further, we employed a recently developed assay that uses delocalization of marker proteins to identify the cellular process that is targeted by an antimicrobial compound [19]. This technique is also known as bacterial cytological profiling [22,23]. Firstly, we examined whether rhodomyrtone causes DNA damage, or inhibits DNA replication, RNA, or protein synthesis. To this end, the relevant enzymes were labelled with GFP and their cellular localization was monitored using fluorescence light microscopy (Fig 1, see S1 Table for strains list). A change in the localization of RecA (DNA-repairing enzyme), DnaN (DNA polymerase subunit), RpoC (RNA polymerase subunit), and RpsB (ribosomal subunit) is indicative of DNA damage, inhibition of DNA replication, RNA synthesis, and protein synthesis, respectively (S1 Fig). As shown in Fig 1, the cellular localization of none of these proteins was affected by rhodomyrtone, indicating that the compound does not inhibit any of these intracellular processes.

### Rhodomyrtone delocalizes membrane proteins

**Fig 2:**
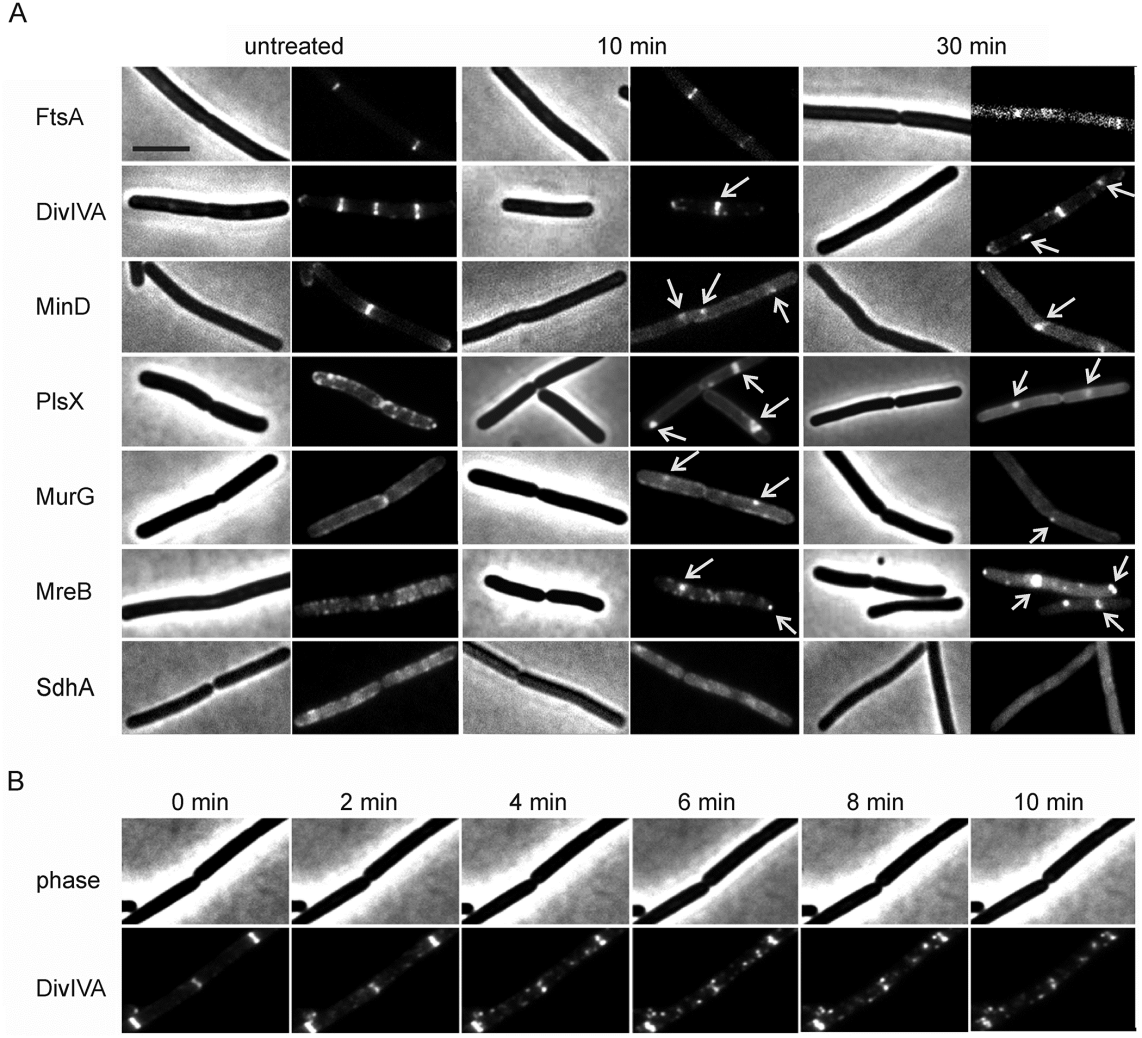
Localization of *B*. *subtilis* membrane proteins after treatment with rhodomyrtone. (A) *B*. *subtilis* strains (see S1 Table for detailed information) were grown until an OD_600_ of 0.3 and subsequently treated with rhodomyrtone (1x MIC). See also overview pictures of GFP-MreB in S2 and S3 Figs. (B) Time lapse microscopy of DivIVA-GFP. *B*. *subtilis* strain HS63 was mounted on an agarose patch, and placed in a flow chamber. Constant medium flow provided sufficient nutrient and oxygen supply during the experiment. Rhodomyrtone was added with the medium flow and pictures were taken every 2 min. See also S1 Movie. Scale bar 2 μm.

Previous studies have shown that rhodomyrtone induces cell shape deformations and cell lysis [17,18], suggesting that the compound could target the bacterial cell envelope. To examine this more closely, we monitored the localization of a representative set of peripheral membrane proteins that have been used to study the effects of membrane-active antibiotics [19,24,25]: (i) the cell division proteins FtsA, DivIVA, and MinD, (ii) the phospholipid synthase PlsX, (iii) the cell shape-determining protein MreB, (iv) the cell wall synthesis protein MurG, and (v) the succinate dehydrogenase SdhA, which is part of the respiratory chain. All proteins showed aberrant localization patterns after 10 min of treatment, which aggravated when treatment was continued for 30 min (Fig 2A). DivIVA, MinD, PlsX, MreB, and MurG accumulated in large fluorescent foci while FtsA and SdhA were completely detached from the membrane. These changes were observed in nearly all cells (see S2 and S3 Figs for overview pictures of GFP-MreB). To test how fast these changes happen, we selected DivIVA for a time lapse experiment. As shown in Fig 2B and S1 Movie, delocalization of DivIVA was already visible after 2 min. Thus, rhodomyrtone rapidly affects multiple membrane-bound processes.

### Cell wall integrity

**Fig 3:**
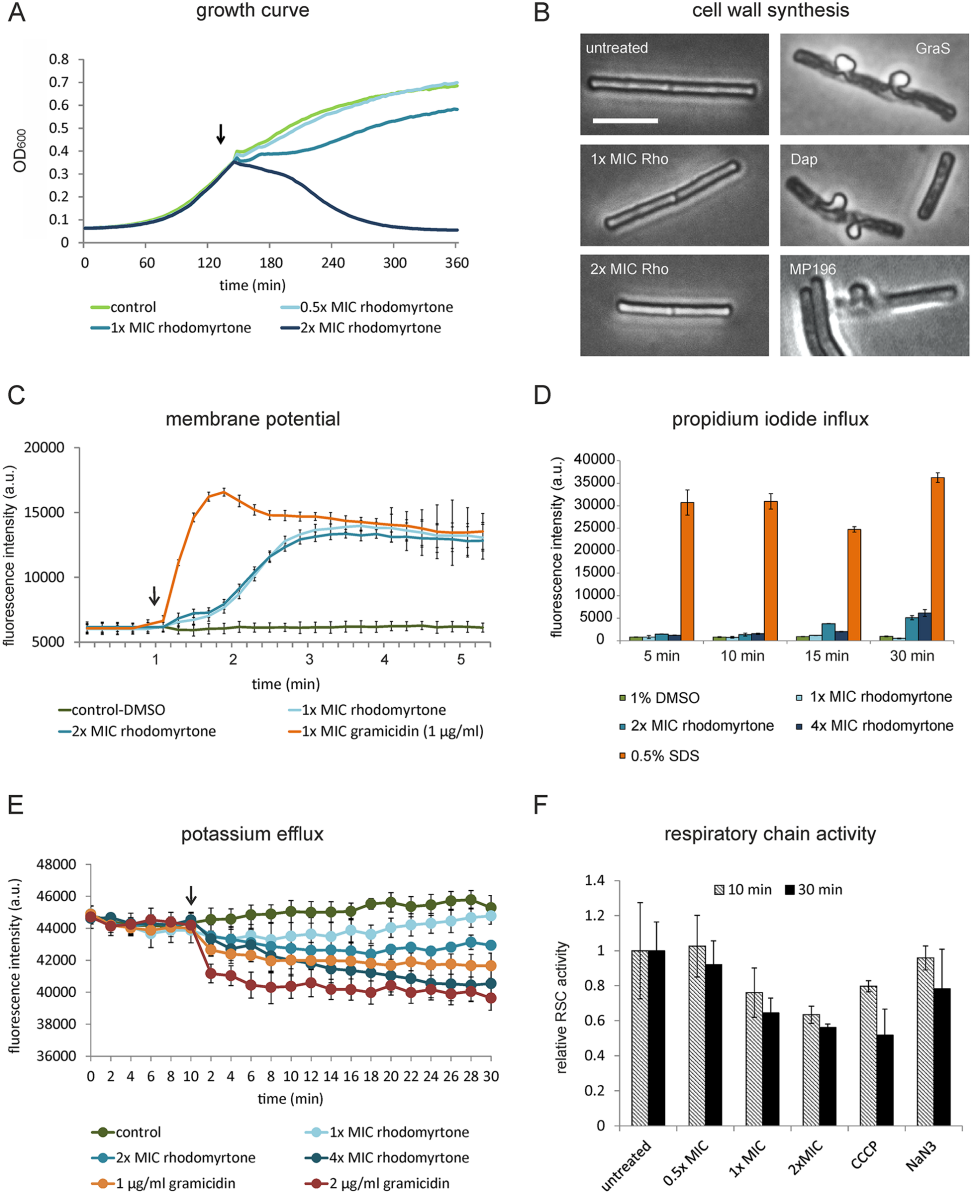
Effects of rhodomyrtone on the cell envelope of *B*. *subtilis*. **(A)** Impact of rhodomyrtone on growth of *B*. *subtilis*. Arrow marks time point of antibiotic addition. **(B)** Influence of rhodomyrtone on cell wall synthesis. *B*. *subtilis* was treated with 0.5 μg/ml (1x MIC) or 1 μg/ml (2x MIC) rhodomyrtone (Rho), 1 μg/ml gramicidin S (GraS), 2 μg/ml daptomycin (Dap), or 10 μg/ml MP196 for 15 min and subsequently fixed in a 1:3 mixture of acetic acid and methanol. Impairment of lipid II synthesis results in extrusion of the cell membrane through holes in the cell wall. Daptomycin, gramicidin S, and MP196, all of which inhibit lipid II synthesis by interfering with cell wall synthesis proteins at the membrane, served as positive controls. Scale bar 2 μm. **(C)** Membrane potential measured with the fluorescent dye DiSC(3)5. Arrow indicates time point of antibiotic addition. **(D)** Staining with propidium iodide. The fluorescent dye is a reporter for the presence of large membrane pores or severe membrane disruption. The detergent SDS, which causes pores by forming membrane micelles, was used as positive control. Error bars represent the standard error of the mean (SEM) of three biological replicates. **(E)** Efflux of potassium ions measured with the intracellular potassium-selective fluorescent dye APG-2. *B*. *subtilis* 168 was stained with APG-2 for 1 h prior to antibiotic addition (arrow). The K^+^/Na^+^ channel ionophore gramicidin served as positive control. **(F)** Activity of the respiratory chain measured by reduction of resazurin to resofurin. CCCP and NaN_3_ were used as positive controls. CCCP uncouples the respiratory chain from the proton gradient and NaN_3_ is an inhibitor of the cytochrome *c* oxidase (complex IV of the respiratory chain). Error bars represent standard deviation of the mean of three biological replicates. Note the different time scales in (A), (C), and (E).

Several studies have shown that rhodomyrtone treatment results in cell shape deformations and some degree of cell lysis [17,18], which fits well with our observation that the localization of MurG, an enzyme involved in the synthesis of the cell wall precursor lipid II, was affected by rhodomyrtone (Fig 2A). However, at 1x MIC we did not observe any lysis based on optical density (OD) measurements, and 2x MIC only led to a gradual OD reduction (Fig 3A). To examine whether rhodomyrtone affects the integrity of the cell wall, we employed a microscopic fixation method to visualize cell wall damage [26]. As shown in Fig 3B, rhodomyrtone did not have an immediate effect on cell wall integrity, which was in sharp contrast to the antimicrobial peptides daptomycin, gramicidin S, and MP196, all of which interfere with the synthesis of lipid II [19,27]. Thus, cell wall synthesis does not seem to be the primary target of rhodomyrtone.

### Membrane barrier function

Previously, we have shown that the localization of several peripheral membrane proteins, including MinD and FtsA, depends on the membrane potential, as the attachment of their membrane-targeting amphipathic helices to the cell membrane is strongly stimulated by the electric potential difference over the membrane [24]. Since we observed delocalization of a number of peripheral membrane proteins (Fig 2), among which MinD and FtsA, we examined whether rhodomyrtone dissipates the membrane potential using the membrane-potentiometric probe DiSC(3)5 [28] (Fig 3C). Indeed, cells were depolarized within approximately 3 min. This was not as fast and strong as the depolarization that occurs with the antibiotic peptide gramicidin, which forms a K^+^/Na^+^ channel in the membrane [29] (Fig 3C), suggesting that rhodomyrtone neither forms an ion channel nor a membrane pore. The latter was confirmed using propidium iodide (PI), a fluorescent indicator that enters cells through pores or severe membrane breaches [30]. As shown in Fig 3D, no significant PI influx was observed, not even after 30 min treatment with 4x MIC.

To test whether rhodomyrtone enhances the ion permeability of the membrane, we measured the intracellular concentrations of potassium with the potassium-selective fluorescent probe APG-2 for 20 min (Fig 3E). Indeed, rhodomyrtone caused a continuous, concentration-dependent leakage of potassium. However, this leakage occurred too slowly (10–30 min) to explain the much quicker (3 min) membrane depolarization (Fig 3C). Furthermore, at 1x MIC cells recovered their potassium levels after approximately 20 min (Fig 3E) but not their membrane potential (S4 Fig).

Depolarization can not only be caused by permeabilization of the membrane but also by impairing the electron transport chain, which generates the proton gradient [27]. In fact, Fig 2A shows that succinate dehydrogenase (SdhA), which channels protons from the TCA cycle into the electron transport chain [31], fully detaches from the membrane when cells are incubated with rhodomyrtone. Importantly, we have shown in previous work that this protein does not delocalize when the proton motive force is dissipated [25]. Thus, delocalization of SdhA is not a general effect of membrane depolarization but specific to rhodomyrtone treatment. To assess whether the activity of the respiratory chain was indeed affected by rhodomyrtone, we measured the reduction rates of resazurin, which is commonly used to measure the reductive capacity of cells [19,32]. Indeed, incubation with rhodomyrtone considerably diminished the reductive capacity of *B*. *subtilis* cells, which is indicative of a strong inhibition of the electron transport chain (Fig 3F). This effect was comparable to that of the proton ionophore carbonyl cyanide m-chlorphenylhydrazone (CCCP), a de-coupler of the electron transport chain, and much stronger than that of sodium azide, which inhibits complex IV of the respiratory chain (Fig 3F). Thus, rhodomyrtone affects both maintenance and generation of the proton motive force.

### Rhodomyrtone affects lipid domains

**Fig 4:**
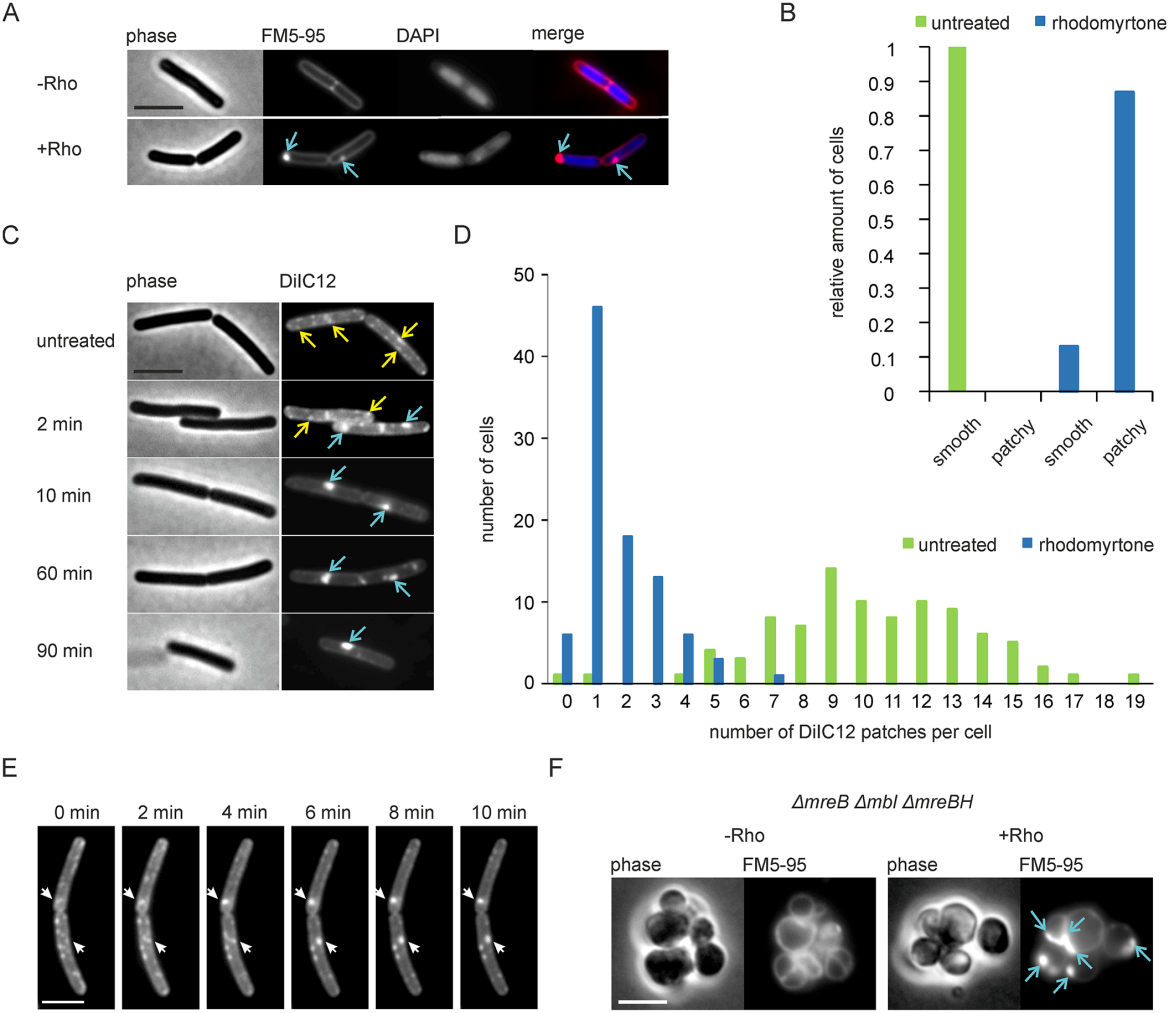
Rhodomyrtone (Rho) causes aberrant fluorescent membrane stains. **(A)** *B*. *subtilis* 168 stained with the membrane dye FM5-95 and the DNA dye DAPI. Blue arrows indicate membrane patches induced by rhodomyrtone. **(B)** Quantification of cells showing FM5-95-stained membrane patches in untreated (green, n = 311) and rhodomyrtone-treated (blue, n = 351) cells. See also overview microscopy pictures in S7 and S8 Figs). **(C)** *B*. *subtilis* 168 stained with the fluid lipid dye DiIC12. Natural RIFs are marked with yellow arrows, rhodomyrtone-induced fluid lipid accumulations with blue arrows. **(D)** Quantification of membrane foci per cells in untreated (green, n = 91) and rhodomyrtone-treated (blue, n = 93) cells. See also overview microscopy pictures in S10 and S11 Figs. **(E)** Time lapse microscopy showing fusion of native RIFs into large fluid lipid accumulations. **(F)** FM5-95 stain of *B*. *subtilis* strain 4277 (*ΔmreB Δmbl ΔmreBH*). Blue arrows indicate membrane patches induced by rhodomyrtone. Scale bars 2 μm.

While our results showed that rhodomyrtone affects different membrane functions, it is still unclear how the compound achieves these membrane effects. In order to shed light on its molecular mechanism, we first investigated the effect of rhodomyrtone on membrane organization using the fluorescent membrane dye FM5-95. Addition of the compound caused highly fluorescent membrane foci in almost 90% of the cells (Fig 4A and 4B, see S5 and S6 Figs for overview pictures), while it did not affect the nucleoid (Fig 4A, see S7 and S8 Figs for overview pictures). Such aberrant membrane stains have also been observed when the membrane potential was dissipated with CCCP, which has been attributed to the accumulation of highly flexible lipids [25]. A high concentration of such flexible lipids, i.e. lipids with short, branched and/or unsaturated fatty acid chains, increases local membrane fluidity (liquid-disordered state) and thus facilitates the insertion of fluorescent membrane probes and/or increases their fluorescence quantum yield, resulting in strongly enhanced fluorescence signals [19,25,33]. In logarithmically growing *B*. *subtilis* cells such flexible lipids will form microscopically visible fluid microdomains (S9 Fig) stimulated by the actin homologue MreB [24]. These regions of increased fluidity (RIFs) can be stained with the fluorescent lipid-mimicking dye DiIC12, which has a strong preference for fluid membrane domains due to its short acyl chain [25]. When DiIC12-stained cells were treated with rhodomyrtone, the regularly distributed RIFs quickly collapsed into large foci (Fig 4C), indicating that the strongly fluorescent FM5-95 patches are indeed enriched in flexible membrane lipids. Quantification of the number of DiIC12 foci per cell showed a clear reduction of multiple RIFs to one or few large DiIC12-stained fluid lipid foci per cell (Fig 4D, see S10 and S11 Figs for overview pictures), suggesting that RIFs might fuse together to generate these domains, a notion that was supported by time lapse microscopy (Fig 4E).

RIFs are formed by membrane-attached MreB polymers by a yet unknown mechanism [25,34]. Since MreB was delocalized by the compound (Fig 2A), we tested whether the rhodomyrtone-induced clustering of RIFs requires MreB or one of its homologues (Mbl, MreBH) using a *ΔmreB Δmbl ΔmreBH* deletion mutant. This mutant forms round cells since it lacks the longitudinal organization of peptidoglycan synthesis [35]. As shown in Fig 4F, rhodomyrtone was still able to cause fluorescent membrane foci, demonstrating that MreB is not required for this.

### Effect on membrane fluidity

**Fig 5:**
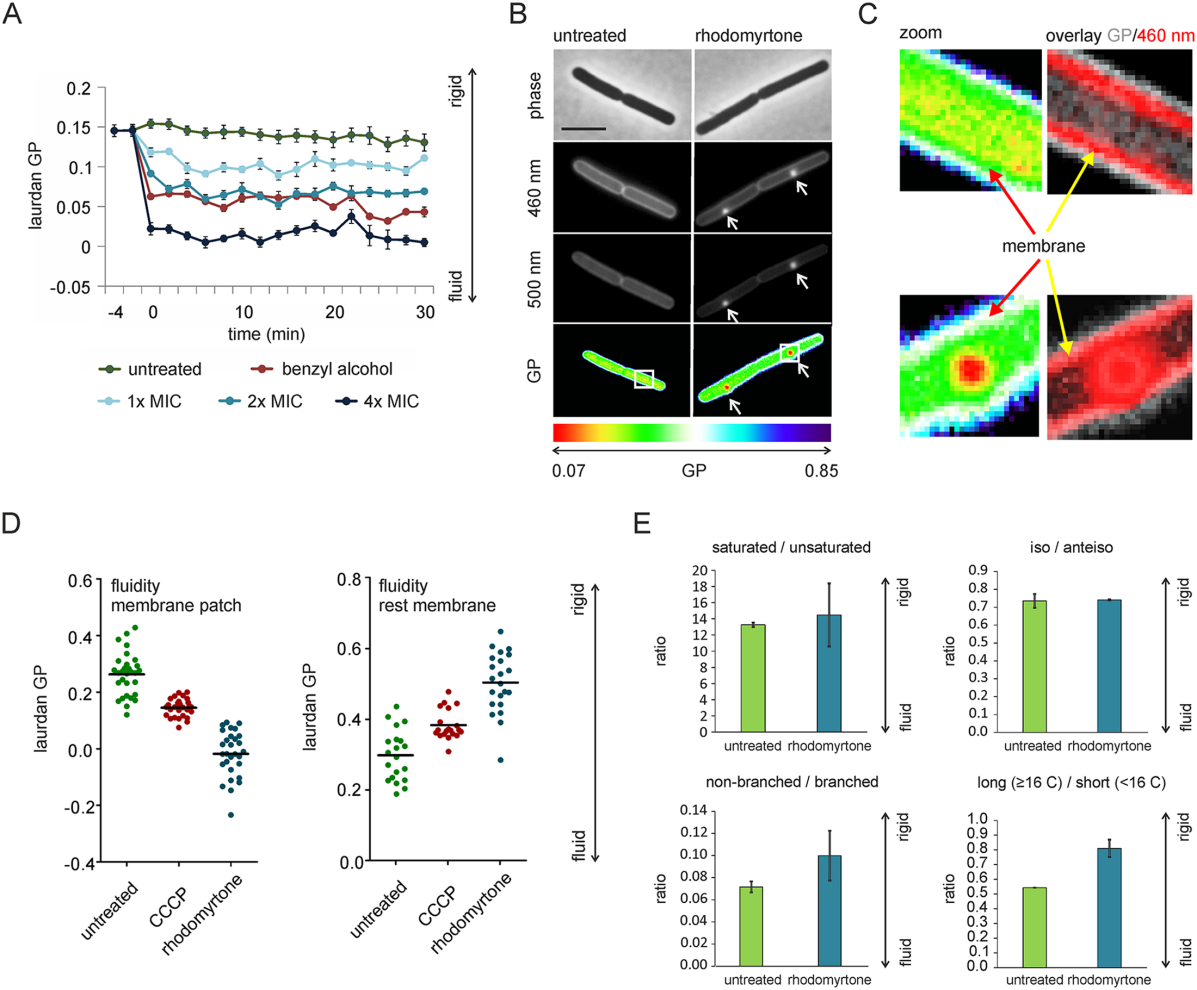
Rhodomyrtone causes highly fluid membrane domains. **(A)** Membrane fluidity of *B*. *subtilis* 168 treated with different concentrations of rhodomyrtone measured with laurdan generalized polarization (laurdan GP, on a scale from -1 (very fluid) to 1 (very rigid)). Benzyl alcohol served as control for fluidization. **(B)** Laurdan GP microscopy of *B*. *subtilis* 168. Cells were treated with 1x MIC of rhodomyrtone for 10 min. Arrows indicate fluid membrane patches. Scale bar 2 μm. Boxes represent areas magnified in (C). **(C)** Rigidification of the rest membrane. Arrows indicate position of the cell membrane. Left panels show magnified cutout from (B) with the same color scale. Right panels show a false-colored image depicting the laurdan GP picture in grey and the laurdan fluorescence stain (460 nm) in red to visualize the position of the plasma membrane (see Material and methods for details of GP calculation from images). The GP shifts from green (GP of ~0.3) to white (GP of ~0.5) showing rigidification of the membrane. **(D)** Quantification of membrane fluidity in individual membrane patches and in the rest membrane of cells stained with laurdan. 100 μM CCCP was used as positive control. **(E)** Fatty acid adaptation of *B*. *subtilis* after one doubling time under exposure to sub-inhibitory concentrations (0.25 μg/ml, 0.5x MIC) of rhodomyrtone. This concentration was chosen based on the growth effect of the rhodomyrtone-treated cultures in large culture flasks (see S15 Fig). Error bars represent standard error of the mean of two biologically independent replicate experiments.

To determine whether the clustering of flexible (= fluidizing) lipids affects overall membrane fluidity, we employed the fluorescence polarization probe laurdan, which changes its fluorescence properties depending on head group spreading and fatty acid chain flexibility [36]. Addition of rhodomyrtone resulted in a rapid (2 min), concentration-dependent reduction of laurdan generalized polarization (GP), which is indicative of an increase in membrane fluidity (Fig 5A), as can be seen from the positive control, the known membrane fluidizer benzyl alcohol [37].

In order to examine how fluidity changes at the single cell level, we employed laurdan microscopy (Fig 5B–5D). In line with the focal enrichment of flexible lipids, membrane foci of highly increased fluidity were apparent, and the rest of the cell membrane showed a clear reduction in fluidity (liquid-ordered state). Thus, the overall increase in membrane fluidity observed in Fig 5A is caused by the strongly fluid membrane foci in the cell. This effect is enhanced by the accumulation of laurdan in these fluid membrane areas (Fig 5B), resulting in higher signal intensities in these areas (S12 Fig). Interestingly, when we compared the fluid membrane patches formed by rhodomyrtone with the fluid lipid patches formed by CCCP, we found that rhodomyrtone had a substantially stronger local fluidizing effect (Fig 5D), supporting the notion that rhodomyrtone-induced patches are fundamentally different from fluid lipid clusters formed by CCCP through MreB delocalization [25].

In order to show that the effects of rhodomyrtone on membrane fluidity are not simply an effect of growth inhibition, we treated cells with ciprofloxacin, which does not target the bacterial membrane but kills bacteria by inhibiting DNA synthesis. Incubation of *B*. *subtilis* cells for 10 min with lethal concentrations of ciprofloxacin did not affect overall membrane fluidity and did not result in clear fluid membrane foci (S13 Fig).

If focal membrane fluidization is indeed a key feature of rhodomyrtone activity, it is likely that bacteria will attempt to adjust their fatty acid composition to compensate for these changes. Indeed, when cells were incubated with sub-inhibitory concentrations of rhodomyrtone, cells showed a significant decrease of short chain fatty acids in favor of long chain fatty acids, which will increase membrane rigidity (Fig 5E and S2 and S3 Tables). It should be noted that we did not observe changes in the ratios of *iso-* to *anteiso*-branched chain fatty acids or saturated to unsaturated fatty acids, which are the major adaptation strategy of *B*. *subtilis* in response to physical changes of membrane fluidity [38,39]. Since *B*. *subtilis* adapts its fatty acid composition towards higher membrane rigidity, it seems that the strong focal fluidization of the membrane is the major problem for cells rather than the rigidification of the rest of the cell membrane.

### Membrane invaginations

**Fig 6:**
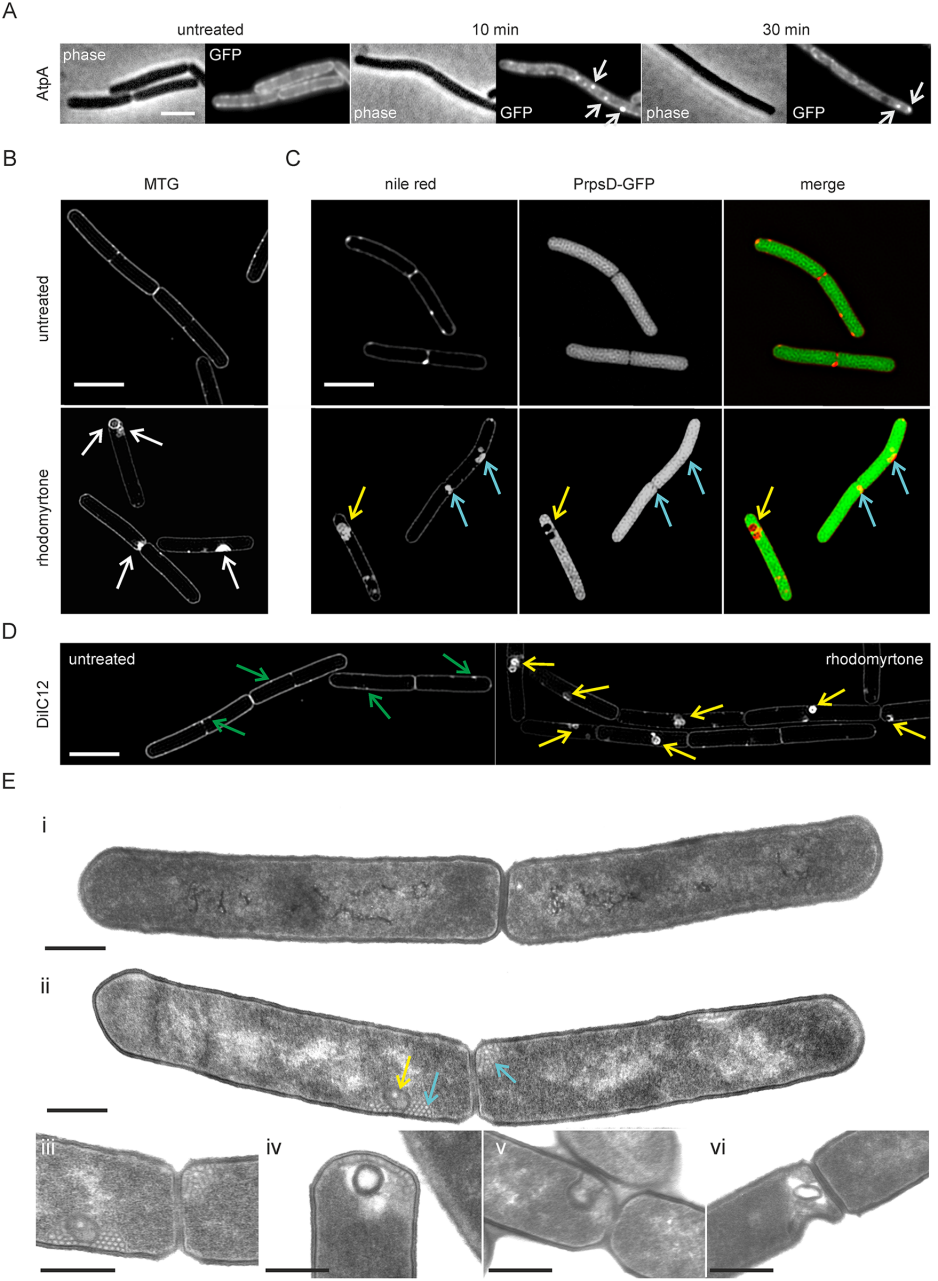
Rhodomyrtone causes membrane invaginations. **(A)** Delocalization of AtpA-GFP. **(B)** SIM images of *B*. *subtilis* 168 cells stained with mitotracker green (MTG) treated with 1x MIC rhodomyrtone for 10 min (lower panel, untreated cells in the upper panel). **(C)** SIM images of *B*. *subtilis* strain bSS82 expressing cytosolic GFP from the strong ribosomal *PrpsD* promoter stained with Nile red. Upper panels show untreated cells, lower panels show cells treated with 1x MIC rhodomyrtone for 10 min. The absence of a green fluorescence signal indicates the presence of large membrane invaginations (yellow arrows). Blue arrows indicate small membrane invaginations. **(D)** SIM images of *B*. *subtilis* 168 stained with DiIC12. Note that DiIC12 clearly stains rhodomyrtone-induced membrane invaginations (yellow arrows). Native RIFs only appear as small foci of increased fluorescence (green arrows), but not as invaginations or intense patches in untreated cells. Scale bars 2 μm. **(E)** TEM pictures of *B*. *subtilis* 168 treated with 1x MIC rhodomyrtone for 10 min. **(i)** Untreated control cell, **(ii)** representative cell treated with rhodomyrtone. Yellow arrow indicates a large intracellular membrane vesicle structures, blue arrows indicate small membrane vesicles. **(iii-vi)** Details of cells treated with rhodomyrtone. Membrane vesicles and/or invaginations occurred in all longitudinally cut cells that were observed in the rhodomyrtone-treated samples, while the untreated cells that we observed never showed these deformations (n ≥ 20). Scale bars 0.5 μm.

In previous studies we have shown that the highly fluorescent membrane spots that are formed when cells are treated with membrane potential-dissipating drugs, are not a consequence of the accumulation of extra membrane material, e.g. due to membrane invagination [25]. This was proven by showing that the localization of the transmembrane FoF1 ATP synthase remained undisturbed and that there was no accumulation of this protein at areas that showed strong fluorescence of membrane dyes. It was therefore remarkable that when we repeated this experiment with rhodomyrtone, AtpA, the subunit of the FoF1 ATP synthase complex, became delocalized and accumulated in foci when cells were treated with rhodomyrtone (Fig 6A, S14 Fig), suggesting that the compound does actually lead to membrane invaginations. To examine this, we used super-resolution Structured Illumination Microscopy (SIM). Cell membranes were stained with mitotracker green, which provides excellent SIM contrast and optimal resolution of membrane structures. After 10 min incubation with rhodomyrtone, membrane invaginations and large intracellular vesicles became clearly visible (Fig 6B). Since SIM image reconstruction can sometimes produce visual artefacts [40], we confirmed the presence of membrane invaginations using a *B*. *subtilis* strain expressing cytosolic GFP from the strong ribosomal *PrpsD* promoter (Fig 6C) [40]. Indeed, the large vesicle-like structures (yellow arrows) lacked GFP, indicating that they originated from cell membrane invaginations. Some of the smaller membrane invaginations (blue arrows) did not clearly show a displaced GFP signal, which can be the case when their internal volume is below SIM resolution. SIM microscopy of DiIC12-stained cells confirmed that the invaginations accumulated the fluid membrane probe DiIC12, as expected (Fig 6D).

To confirm the generation of internal membrane vesicles by rhodomyrtone with an independent technique, we performed transmission electron microscopy (TEM) (Fig 6E). In line with the SIM data, we observed large intracellular membrane structures, some of which were filled (Fig 6Eiii and 6Ev) and others devoid of cytosolic material (Fig 6Eiv and 6Evi), as well as smaller membrane vesicles (Fig 6Eiii and 6Eiv).

Similar membrane invaginations have been observed after overexpressing the acetyl-CoA carboxylase AccABCD, the enzyme catalyzing the rate-limiting step of fatty acid synthesis [41,42]. However, it takes several hours of AccABCD overexpression to form invaginations of significant size [42], whereas rhodomyrtone already causes large membrane invaginations after only 2–10 min of treatment. Moreover, inhibition of protein and lipid synthesis by chloramphenicol and triclosan, respectively, did not prevent the formation of invaginations by rhodomyrtone (S15 Fig), further indicating that the compound is directly responsible for membrane invaginations.

### Trapping membrane proteins

**Fig 7:**
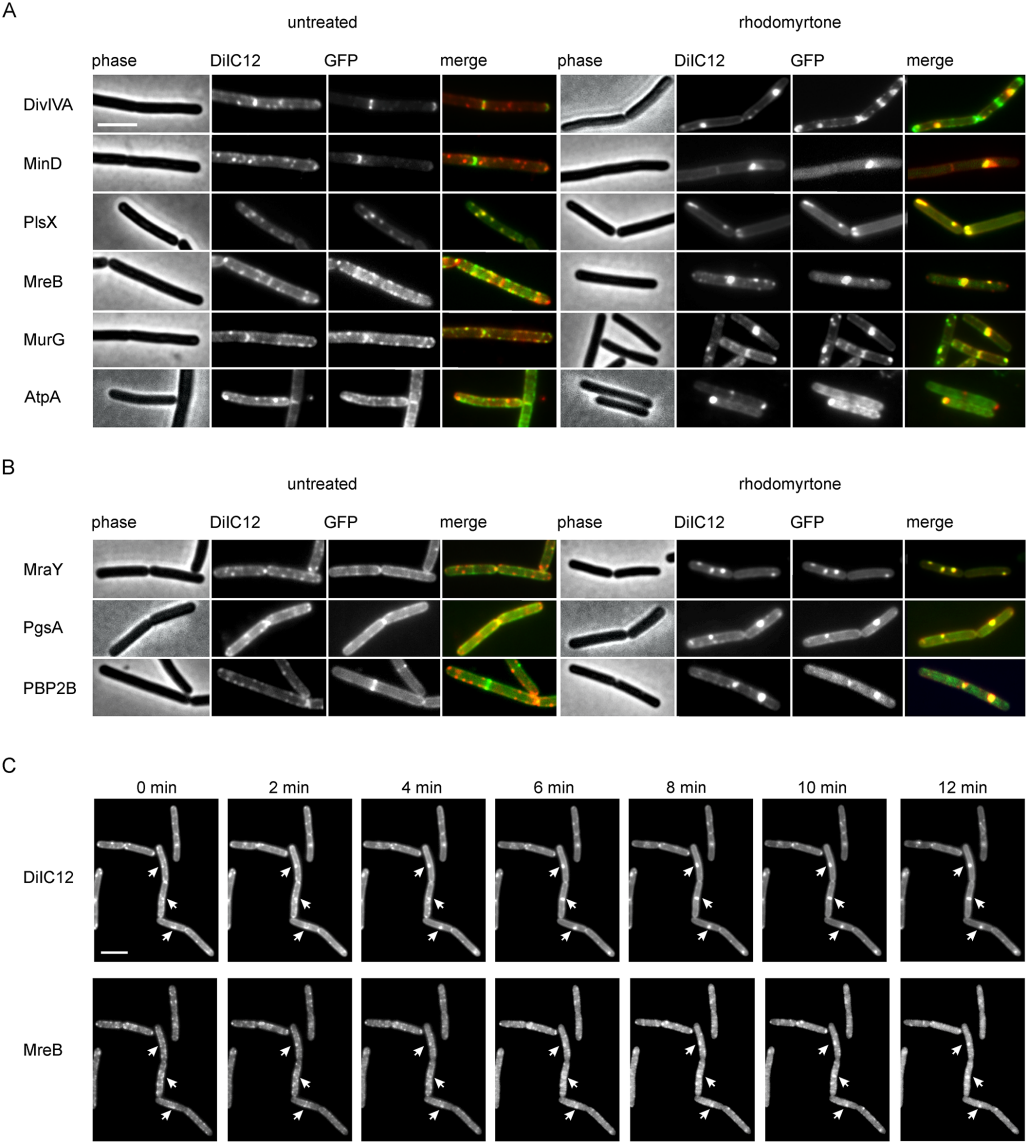
Co-localization of delocalized peripheral **(A)** and integral **(B)** membrane proteins with fluid lipid accumulations. *B*. *subtilis* strains (see S1 Table for detailed information) were treated with 1x MIC rhodomyrtone for 10 min. Membranes were stained with the fluid lipid domain dye DiIC12. **(C)** Time lapse microscopy of DiIC12-stained cells expressing GFP-MreB treated with 1xMIC rhodomyrtone. Several fluid lipid accumulations and corresponding accumulations of MreB are indicated by arrows. Scale bars 2 μm.

Since AtpA, our reporter for membrane invagination, accumulated into foci (Fig 6A), and the rhodomyrtone-induced membrane patches (= invaginations) were enriched in fluidizing lipids (Fig 4C–4E), we wondered whether these fluid membrane structures could attract membrane proteins in general, resulting in the patchy GFP localization patterns observed in Fig 2. To examine this, we stained cells expressing GFP-tagged peripheral membrane proteins, and cells expressing GFP-tagged integral membrane proteins, together with the fluid lipid domain dye DiIC12. As shown in Fig 7A, all tested peripheral membrane proteins showed a clear accumulation at bright DiIC12 spots. Importantly, Fig 7B shows that also the transmembrane proteins MraY and PBP2B, involved in cell wall biosynthesis, and the phospholipid synthase PgsA accumulated at the rhodomyrtone-induced fluid lipid clusters. We selected MreB as exemplary protein for a time lapse experiment to follow protein localization during the generation of fluid lipid clusters (Fig 7C). The accumulation of MreB clearly correlated with the formation of DiIC12-stained domains (arrows in Fig 7A) and was retained there. Together, these results indicate that both peripheral and integral membrane proteins become attracted by the fluid membrane domains and are then trapped in the resulting membrane vesicles caused by rhodomyrtone (see S16 Fig for the transmembrane protein MraY). These membrane domains were stable. Cells were neither able to remove these membrane structures and re-establish a normal membrane morphology (Fig 4C), nor to recover normal protein localization patterns (example MraY in S16 Fig) for the time spans observed in this study (60–90 min). Clearly, this trapping of membrane proteins in vesicles will strongly impair their function and explains the strong antimicrobial effect of rhodomyrtone.

### Molecular dynamics simulations

**Fig 8:**
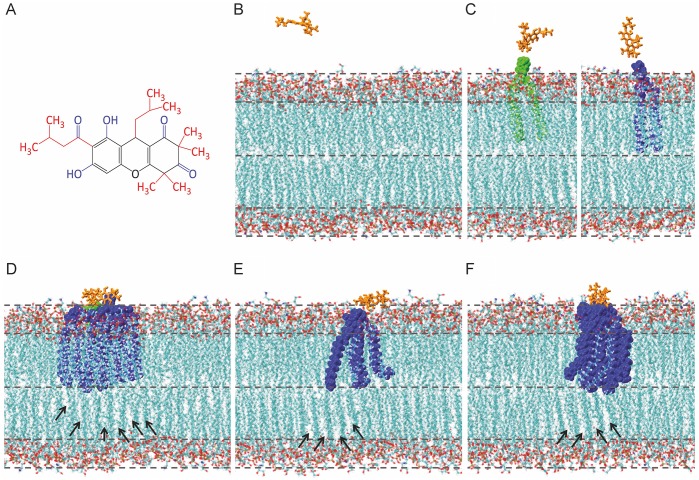
Molecular dynamics simulations of rhodomyrtone binding to 3:1 POPG/POPE bilayers (100 ns). **(A)** Structure of rhodomyrtone. Polar residues are displayed in blue, non-polar residues in red. **(B)** Membrane state before binding of rhodomyrtone (orange). **(C)** POPE (green) and POPG (blue) are dragged out of the bilayer by rhodomyrtone. **(D)** The compound induces tighter head group packing in the outer membrane leaflet, promoting wider fatty acid spreading in the inner leaflet (arrows), resulting in higher membrane fluidity. **(E-F)** Binding of rhodomyrtone to lipid head groups affects the arrangement of fatty acid chains within the bilayer. Rearrangement of lipids bound by rhodomyrtone (blue) also affects fatty acid chain packing in the inner leaflet (arrows).

Structurally, rhodomyrtone does not resemble a typical membrane-integrating agent. While it does contain both polar and non-polar groups, these are spread out over the molecule and do not define distinct amphipathicity (Fig 8A), as is typically the case for membrane-integrating antimicrobials [43]. Thus, it seemed rather unlikely that the compound intercalates in between phospholipids. To gain insight into how rhodomyrtone could interact with and affect phospholipids, we performed molecular dynamics simulations using a 3:1 mixture of 1-palmitoyl-2-oleoyl-posphatidylglycerol (POPG) and 1-palmitoyl-2-oleoylphosphatidylethanolamine (POPE), which mimics the Gram-positive cell membrane. As expected, rhodomyrtone was unable to penetrate into the lipid bilayer. Instead, it temporarily attached to phospholipid head groups (Fig 8B and 8C) resulting in repeated events of binding and release (S2 Movie). This binding involved both the polar and non-polar residues of the antibiotic but did not involve any selectivity for either PG or PE (Fig 8C, S4 Table). Binding of rhodomyrtone to phospholipid head groups affected the vertical arrangement of the phospholipid bilayer by temporarily dragging lipids out of the membrane (Fig 8C), resulting in tighter head group packing (Fig 8D) and rearrangement of the bound lipids in the outer membrane leaflet (Fig 8E and 8F). These changes in the outer leaflet also affected lipid packing in the inner leaflet in terms of spreading of fatty acid chains, promoting locally increased membrane disorder (Fig 8D–8F), which would explain the fluidizing effect of rhodomyrtone. To investigate whether increased concentrations of rhodomyrtone molecules showed a different effect, we also performed molecular dynamics simulations with two, four, and eight molecules of rhodomyrtone. Despite the fact that rhodomyrtone molecules tended to form clusters, the effects on lipid packing were similar (S17 Fig).

### General lipid bilayer effect

Together with our *in vivo* data, the molecular dynamics simulations indicated that rhodomyrtone is the first membrane-active antibiotic molecule that does not integrate into the lipid bilayer and causes severe lipid packing defects solely by interaction with phospholipid head groups. Molecular dynamics simulations showed a transient interaction between rhodomyrtone and phospholipid head groups, suggesting that it should be possible to remove the compound by washing. Indeed, when we treated *B*. *subtilis* with 1x MIC of rhodomyrtone for 2 min, cells recovered completely from membrane deformations, when they were washed and further grown in fresh medium (S18 Fig). This is in contrast to the membrane-intercalating antibiotic daptomycin, which cannot be washed out [19]. Cells treated with higher concentrations of rhodomyrtone or incubated for longer than 2 min were unable to recover, probably due to irreparable membrane damage, which is well in line with the formation of non-reversible membrane invaginations.

**Fig 9:**
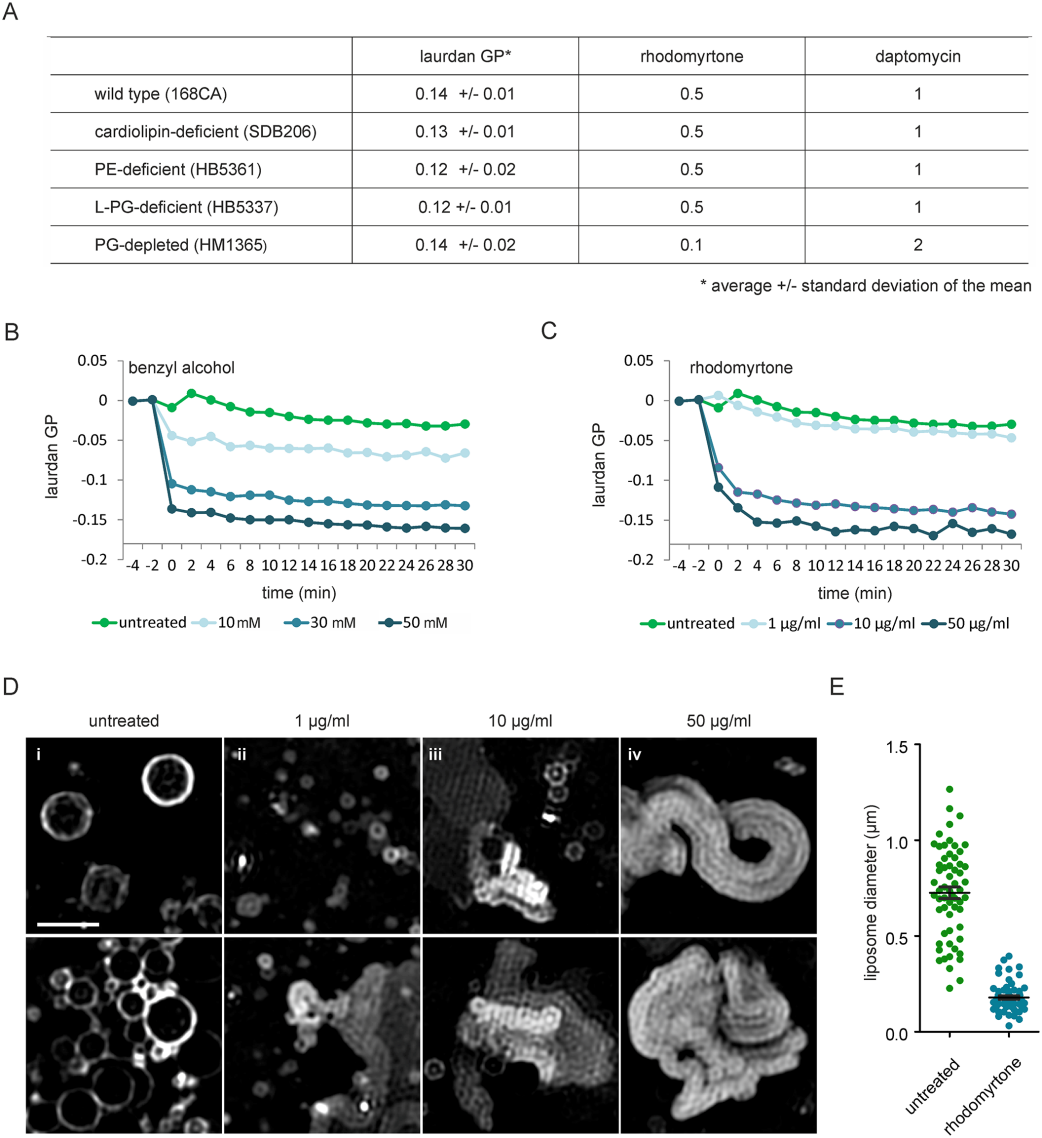
Rhodomyrtone acts directly on the lipid bilayer without specific head group preference. **(A)** Minimal inhibitory concentrations (μg/ml) of rhodomyrtone against different lipid head group mutants of *B*. *subtilis*. Daptomycin was used as a control. **(B)** Laurdan GP spectroscopy of liposomes prepared from *E*. *coli* polar lipid extract exposed to different concentrations of rhodomyrtone. Compound to lipid ratios: 1 μg/ml = 1:400, 10 μg/ml = 1:40, 50 μg/ml = 1:7. **(C)** Laurdan spectroscopy of liposomes treated with the membrane fluidizer benzyl alcohol. Compound to lipid ratios: 10 mM = 12.5:1, 30 mM = 37.5:1, 50 mM = 62.5:1. **(D)** SIM microscopy images of liposomes prepared from *E*. *coli* polar lipid extract exposed to different concentrations of rhodomyrtone for 5 min. Liposomes were stained with DiIC12. Compound to lipid ratios as in **(B)**. Upper and lower rows show two examples from the same samples. Scale bar 1 μm. **(E)** Diameter of liposomes from **(D i)** and **(D ii)**. Black bars represent mean with SEM.

The molecular dynamics simulations also indicated that rhodomyrtone does not display any selectivity for PG or PE lipids. To test this *in vivo*, we tested whether the lipid head group composition plays a role in the activity of rhodomyrtone, using *B*. *subtilis* mutants devoid of either cardiolipin, phosphatidylethanolamine (PE), lysyl-phospatidylglycerol (L-PG), or depleted for phosphatidylglycerol (PG), the four main phospholipid species in bacteria. If rhodomyrtone preferentially binds to one of these phospholipid species, it should be less active against a deficient strain. As a positive control we tested daptomycin, which is less effective against strains depleted for its docking molecule PG [44–46]. Whereas the MIC of daptomycin indeed doubled for PG-depleted cells, none of the tested mutations reduced the activity of rhodomyrtone (Fig 9A). This supports the molecular dynamics results suggesting that rhodomyrtone has no preference for a specific lipid head group type. It should be noted that a slightly enhanced rhodomyrtone activity was observed for the PG-depleted strain, which could be interpreted as a hampering effect on the action of rhodomyrtone by PG. However, it has been shown before that PG depletion leads to severe growth defects and morphological changes [25], and the lower MIC is likely a consequence of the reduced general fitness of PG-depleted cells. To prove this, we determined the MIC of several other antibiotics with different targets and indeed observed that PG-depleted cells were more sensitive to all of them (S5 Table).

Our molecular dynamics simulations suggested that membrane lipids are the direct target of rhodomyrtone. To confirm this, we performed an *in vitro* laurdan GP experiment using liposomes prepared from *Escherichia coli* polar lipid extract. Benzyl alcohol, which has as direct fluidizing effect on lipid membranes [47–49], was used as positive control (Fig 9B). As shown in Fig 9C, the addition of rhodomyrtone caused a clear reduction in laurdan GP at concentrations that roughly correspond to the estimated compound to lipid ratio under our *in vivo* experiments (1:20, further commented on in the Method section). Thus, membrane fluidization is a direct effect of rhodomyrtone on the phospholipid bilayer and does not require a protein target.

Molecular dynamics simulations also showed that rhodomyrtone induced tighter head group packing in the outer membrane leaflet (Fig 8D), which could lead to bending of the membrane and induction of membrane invaginations. To test whether rhodomyrtone is able to bend membranes, we analyzed the shape of liposomes treated with rhodomyrtone using SIM microscopy. At low rhodomyrtone concentrations liposomes became much smaller than in an untreated control sample (Fig 9D and 9E), indicating membrane remodeling and vesiculation [50,51]. Higher concentrations led to severe deformation of liposomes resulting in strongly bend lipid structures (Fig 9D). Membrane deformation and vesiculation are in line with our SIM and TEM data (Fig 6B–6E). Clearly, rhodomyrtone itself is capable of bending the membrane and forming small membrane vesicles.

### Fluid lipid accumulations and membrane invaginations in pathogens

**Fig 10:**
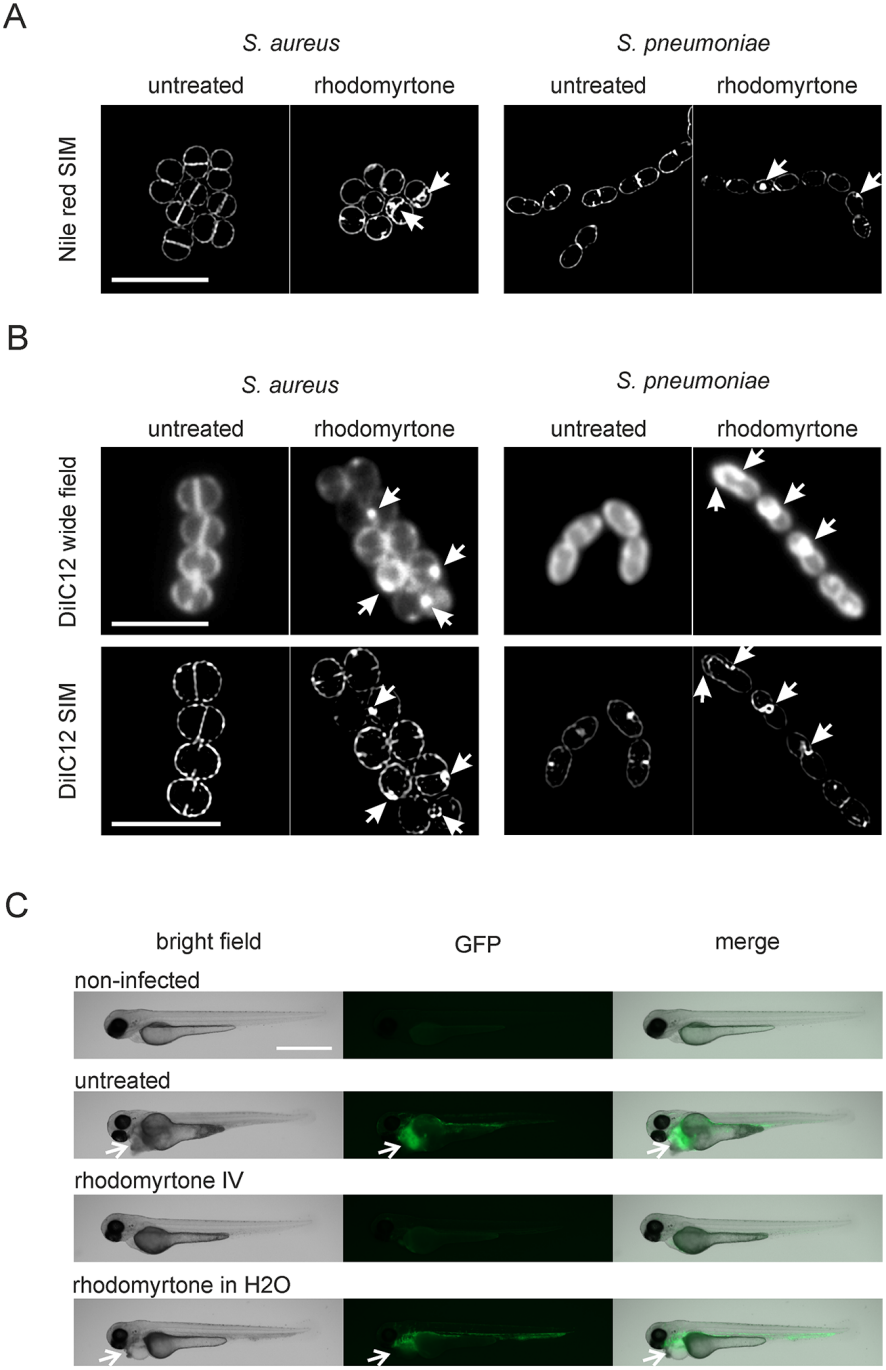
Effects of rhodomyrtone on *S*. *aureus* and *S*. *pneumoniae* and efficacy of rhodomyrtone against *S*. *pneumoniae* infection. *S*. *aureus* RN4220 and *S*. *pneumoniae* D39 were grown until early log phase and treated with 1 μg/ml rhodomyrtone for 10 min. **(A)** SIM pictures of *S*. *aureus* and *S*. *pneumoniae* stained with Nile red. Arrows indicate membrane invaginations in rhodomyrtone-treated cells. Scale bar 2 μm. **(B)** DiIC12-stained cells show bright fluorescent patches after treatment with DiIC12 (arrows, upper panels). Corresponding SIM images show that these patches are membrane invaginations (arrows, lower panels). Scale bars 2 μm. **(C)** Rhodomyrtone reduces the bacterial load and reduces damage to the heart region in zebra fish embryos infected with *S*. *pneumoniae*. One day old zebra fish embryos were injected with 160 CFU of *S*. *pneumoniae* JWV500 expressing HlpA-GFP in the tail vein. Fish were treated with two injections (45 and 75 min post infection) of 25 ng rhodomyrtone each. For treatment in water, 5 μg/ml rhodomyrtone were directly added to the water. Pictures were taken 18 hours post infection. Note the visible damage to the heart region of the fish caused by *S*. *pneumoniae* infection (arrows, left panel) and the bright fluorescence spots, indicating the presence of *S*. *pneumoniae* JW500 in this region (arrows, middle panel). Reduction of the fluorescence signal compared to untreated cells was consistently observed in all rhodomyrtone-injected fish (see also S18 Fig), but not in fish treated by addition of the compound to the water. Experiments were performed in biological triplicates with 15 fish per condition in each replicate. Scale bar 200 μm.

To determine whether the results obtained with *B*. *subtilis* can be transferred to pathogenic microorganisms, we tested the formation of DiIC12-stained fluid lipid accumulations and the formation of membrane vesicles in *S*. *aureus* and *S*. *pneumoniae*. Indeed, we observed clear membrane invaginations in both organisms after treatment with 1x MIC rhodomyrtone (1xMIC, 1 μg/ml) for 10 min, using the Nile red membrane dye (Fig 10A). When *S*. *aureus* and *S*. *pneumoniae* were stained with the fluid lipid domain dye DiIC12, bright fluorescent patches became visible, indicating the accumulation of fluid lipid domains (Fig 10B). High resolution SIM microscopy of the same cells, confirmed that these fluid lipid-enriched domains are membrane invaginations (Fig 10B), showing that rhodomyrtone has the same principal mechanism of action against clinically relevant pathogens.

### Rhodomyrtone is effective against *S*. *pneumoniae* infection

Although the rhodomyrtone-producing plant *R*. *tomentosa* is used in traditional Asian medicine [52] and several *in vitro* studies have shown promising results in terms of antibacterial activity and safety of rhodomyrtone [5–15], there is so far no information available on its effectiveness in an animal infection model. Therefore, we performed *S*. *pneumoniae* infection experiments with one day old zebra fish embryos, a well-established bacterial infection model [53–55]. To monitor the effect on *S*. *pneumoniae* cells in fish, we used a GFP-expressing strain [53,56]. Using fluorescence microscopy, we could clearly see that rhodomyrtone significantly reduced the bacterial load in the fish (Fig 10C, S19 Fig). Infection of zebra fish embryos with *S*. *pneumoniae* leads to characteristic damage to the heart region of the fish and bloating, which can easily be observed under the microscope (Fig 10C, S19 Fig). Injection of rhodomyrtone notably reduced this symptom (Fig 10C, S19 Fig). These findings show that the compound is effective against *S*. *pneumoniae* infection *in vivo*. Addition of rhodomyrtone to the water did not reduce bacterial load or bloating of the heart region (Fig 10C). We did not observe signs of toxicity at the highest rhodomyrtone concentration injected (Fig 10C, S19 Fig), indicating that there is a therapeutic window for systemic applications.

In line, we did not observe any fluid lipid accumulations up to 200x MIC, when we stained human erythrocytes with the fluid lipid domain dye DiIC12 (S20 Fig), indicating that the induction of membrane invaginations by rhodomyrtone is unique to bacterial membranes. So far, it is unclear which mechanism underlies the selectivity of rhodomyrtone for bacterial cells. Therefore, we performed laurdan spectroscopy of liposomes consisting of either POPG or 1-palmitoyl-2-oleoylphosphatidylcholine (POPC). Interestingly, no membrane fluidization was observed in POPC liposomes (S21 Fig). In contrast to PG, which is a typical bacterial membrane lipid, PC is abundant in mammalian cell membranes but not present in most bacteria. This might provide a first indication why rhodomyrtone is selective for bacterial over mammalian cells.

## Discussion

### Mechanism of action

**Fig 11:**
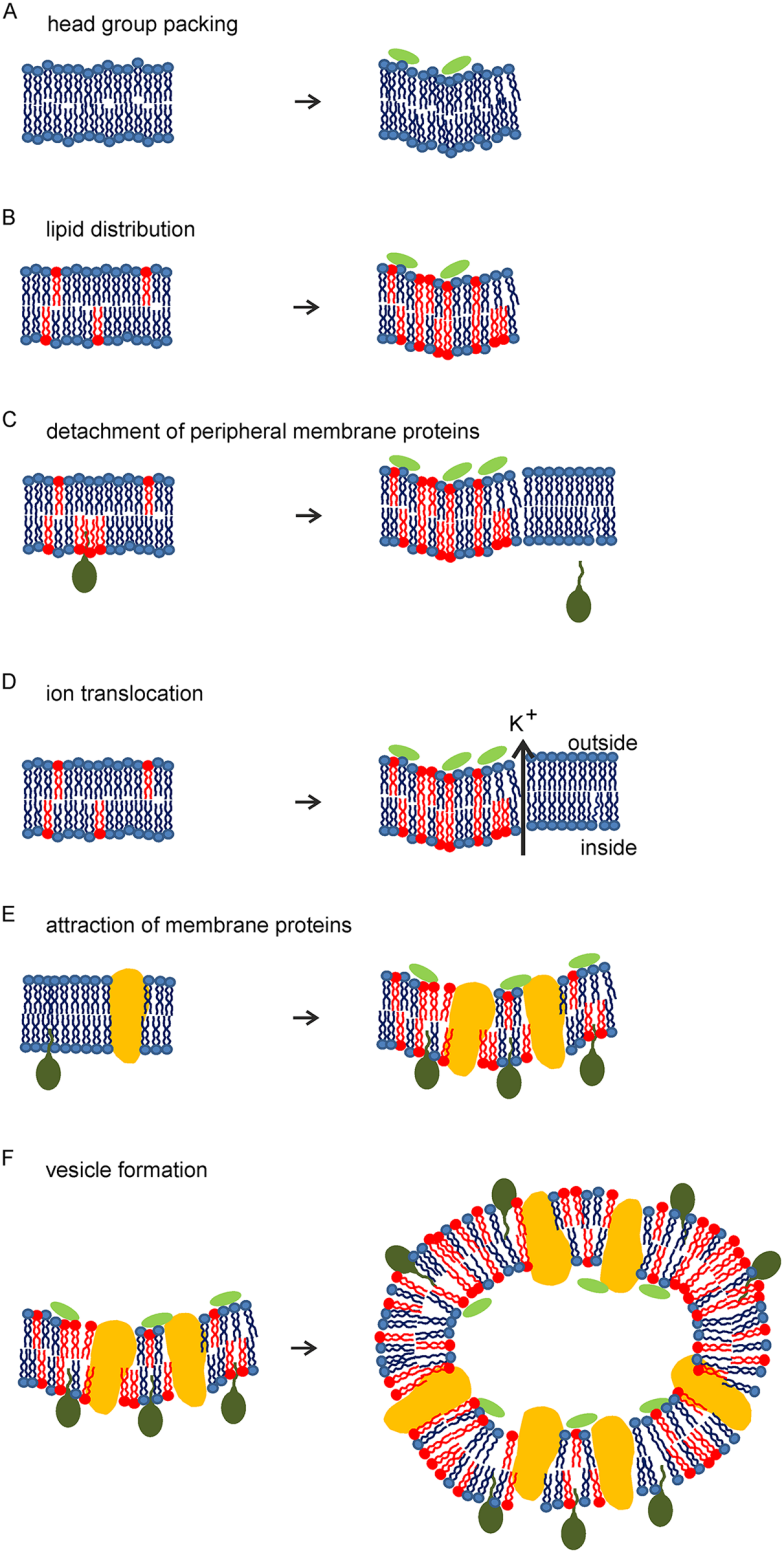
Effects of rhodomyrtone-lipid interaction on the bacterial cytoplasmic membrane (blue) and membrane proteins. **(A)** Rhodomyrtone (light green) causes tighter head group packing in the outer membrane leaflet leading to positive membrane curvature. **(B)** Flexible lipids (red) are attracted to the sites of positive curvature generating highly fluid lipid domains. **(C)** Attraction of flexible lipids to the sites of membrane invaginations leads to rigidification of the rest of the membrane and detachment of peripheral membrane proteins. **(D)** Phase boundary defects between curved fluid membrane domains and the rigidified rest of the membrane increase passive permeability for potassium ions. **(E)** Both peripheral (dark green) and integral (yellow) membrane proteins are attracted to membrane invaginations due to higher local fluidity leading to protein aggregates in the membrane. **(F)** Increased curvature culminates in membrane invaginations, thereby trapping both flexible lipids species and membrane proteins in vesicles.

Since rhodomyrtone does not form large membrane pores and does not strongly affect the cell wall, it has long been thought not to target the bacterial cell envelope [5,6,15]. Here, we show that rhodomyrtone acts on the cytoplasmic membrane via a novel molecular mechanism, resulting in large membrane invaginations and intracellular vesicles that trap membrane proteins. The results of this study are summarized in a working model schematically depicted in Fig 11. Contrary to other membrane-active antimicrobials [43], rhodomyrtone does not have a clear amphipathic membrane-interacting structure or domain and is very unlikely to insert into the cell membrane. Molecular dynamics simulations indicated that multiple repeats of binding and release cause tighter packing of the phospholipid head groups in the outer leaflet, resulting in wider fatty acid spreading in the inner membrane leaflet and bending of the membrane (Fig 11A). In addition, fluidizing lipids, i.e. lipids with short, branched, or unsaturated fatty acids, with a higher degree of flexibility, will locate to these sites since they better fit into this disordered lipid environment than less flexible lipid molecules (Fig 11B). The formation of these highly fluid domains in the membrane has three effects. Firstly, the local accumulation of fluidizing lipids leads to rigidification of the rest of the cell membrane resulting in dissociation of peripheral membrane proteins (Fig 11C), which require a certain degree of fluidity to insert their membrane-binding domains [57]. Secondly, the formation of membrane domains of different thickness causes hydrophobic mismatches that are likely to compromise the membrane barrier function and allow leakage of ions leading to a gradual dissipation of the membrane potential [19,58–62] (Fig 11D). And thirdly, these highly fluid lipid domains attract both peripheral and integral membrane proteins (Fig 11E), since a more fluid lipid environment accommodates most membrane proteins better than a rigid environment [57]. Finally, the combination of local membrane curvature and a high concentration of fluidizing lipids stimulates the formation of membrane invaginations leading to vesicles, effectively trapping the accumulated membrane proteins (Fig 11F).

Trapping of membrane proteins in vesicles by rhodomyrtone disturbs a wide variety of membrane processes. The delocalization of proteins involved in cell shape (MreB), cell wall synthesis (MurG, MraY, PBP2B), and cell division (FtsA, MinD, DivIVA) explains the effects on the cell size and shape of *B*. *subtilis*, *S*. *aureus*, and *S*. *pyogenes* that were observed in earlier studies [17,18,21]. In contrast to the antimicrobial peptides daptomycin, MP196, and gramicidin S, all of which affect the function of the cell wall synthesis protein MurG [19,27], rhodomyrtone did not have an immediate effect on cell wall integrity (Fig 3B), which was surprising considering the fact that the compound delocalizes MurG, MraY, and PBP2B. However, this is well in line with the stress response of *S*. *aureus* to treatment with rhodomyrtone, which did not show upregulation of typical cell envelope stress proteins, such as the *liaRS* two-component system [17,63]. Moreover, cell shape defects caused by rhodomyrtone become only visible after prolonged treatment times of one to four hours [18], suggesting that inhibition of cell wall synthesis is not the main mechanism of growth inhibition.

While rhodomyrtone had no immediate effect on cell wall synthesis, it had a rapid and strong effect on cellular respiration (Fig 3F), probably due to the displacement of respiratory chain proteins such as SdhA and dissociation of the FoF1 ATP synthase complex (Figs 2A and 6A and S17 Fig). This is in agreement with the observed upregulation of metabolic pathways in earlier rhodomyrtone studies [21], since energy depletion (ATP limitation) typically leads to an unspecific inhibition of the synthesis of the main cellular macromolecules [19,27]. Such a strong inhibitory effect on cellular respiration has not yet been observed with other antibiotics that cause membrane protein delocalization, including daptomycin, MP196, and gramicidin S [19,27]. In fact, no other antibiotic tested so far has been able to promote delocalization of the ATP synthase [19,24,27].

### Rhodomyrtone acts different from known antimicrobial compounds

The common view of membrane-targeting antibiotics is that they form pores or ion channels [64]. However, over the last few years a number of membrane-targeting compounds have been discovered that do not form membrane pores, including the antimicrobial peptides daptomycin, cWFW, MP196, and gramicidin S [19,27,65]. While daptomycin specifically inserts into specialized membrane microdomains [19], the other compounds act according to the interfacial activity model, i.e. they do not penetrate deeply enough into the membrane bilayer to form membrane-spanning pores, but rather act at the interface between phospholipid head groups and fatty acid chains of membrane lipids leading to membrane deformation [66]. None of these models applies to rhodomyrtone, which only binds to phospholipid head groups and does not intercalate in between membrane lipids. Thus, rhodomyrtone is the first antibiotic to achieve substantial changes in membrane architecture solely by binding to phospholipid head groups in the outer membrane leaflet.

Daptomycin, MP196, and gramicidin S also affect localization of certain membrane proteins (MurG and PlsX, or MurG and cytochrome *c*, respectively) [19,27] and the antimicrobial peptide cWFW spatially separates peripheral and integral membrane proteins into domains of different fluidity [67]. However, rhodomyrtone is unique in that it strongly affects a very broad range of membrane proteins and traps these proteins in intracellular membrane vesicles.

### Clinical implications

*R*. *tomentosa* leave decoctions are traditionally used in Asia to treat gastrointestinal infections and prevent postpartum infections, and crushed leaves are applied as wound dressings [52]. Rhodomyrtone, isolated from these leaf extracts [8], is not only bactericidal and very potent against a number of important bacterial pathogens, it also belongs to a new compound class and possesses a novel mechanism of action. Although it remained unclear which mechanism underlies its selectivity for bacterial membranes, it has been shown that the compound does not display toxicity against mammalian cells [6,8,11]. Here we could provide a first clue to the mechanism underlying the selectivity of rhodomyrtone, which appears to have a higher affinity for bacterial over mammalian membrane lipids (S20 and S21 Figs). We could also show for the first time that rhodomyrtone is effective against bacteria in an infection model, suggesting a therapeutic window for systemic treatment.

Since rhodomyrtone affects a number of different cellular processes and not a single protein target, a rapid development of resistance against the compound is not expected. In fact, no stable rhodomyrtone-resistant *S*. *aureus* mutant could be isolated in multiple passaging experiments [6]. Bacteria have developed several mechanisms of resistance against membrane-targeting antibiotics. For example, reduction of the PG content (in favor of L-PG or cardiolipin) in the cytoplasmic membrane and cell wall charge modifications are the most effective resistance mechanism against daptomycin and other membrane-targeting compounds [43,44]. In contrast to such compounds, rhodomyrtone does not have a strong preference for either negatively charged (PG) or neutral phospholipid (PE) head groups, and its activity is not affected by changes in the concentration of one of the main phospholipid species in bacteria (Fig 9A). In addition, rhodomyrtone delocalizes important membrane-bound lipid synthases (PlsX, PgsA), thereby complicating possible attempts by the bacterial cell to adapt the membrane lipid composition. Moreover, rhodomyrtone is not charged and therefore is also unlikely to be affected by D-alanylation of lipoteichoic acids in the cell wall, a common adaptation mechanism to positively charged antimicrobial molecules [68]. Therefore, the common resistance mechanisms against other membrane-targeting compounds are likely not effective against rhodomyrtone, minimizing the risk of cross-resistance with other antibiotics. In fact, rhodomyrtone is active against vancomycin-resistant strains of *S*. *aureus* [6]. Furthermore, it is effective against non-growing antibiotic-tolerant bacterial cultures (S22 Fig), suggesting that it could also eradicate persister cells that cause dormant and recurring infections. In conclusion, rhodomyrtone is a potent new antibiotic candidate that belongs to a novel compound class and possesses a unique mechanism of action.

## Material and methods

### Antibiotics

Rhodomyrtone was extracted from leaves of *Rhodomyrtus tomentosa* with 95% ethanol as described previously [21,69]. MP196 was synthesized as described before [70]. Daptomycin was purchased from Novartis. All other antibiotics were purchased from Sigma Aldrich in the highest possible purity. Rhodomyrtone, gramicidin, gramicidin S, triclosan, CCCP, MP196, benzyl alcohol, and mitomycin C were dissolved in sterile DMSO. Daptomycin, ciprofloxacin, and sodium azide were dissolved in sterile water. Chloramphenicol, erythromycin, and rifampicin were dissolved in ethanol. The bactericidal concentration of ciprofloxacin was determined by plating cultures treated with the antibiotic for 10 min on unselective agar plates. Treatment with 1 μg/ml ciprofloxacin resulted in no CFU after overnight incubation.

### Bacterial strains and growth conditions

*B*. *subtilis* strains used in this study are listed in S1 Table. Unless otherwise noted, all strains were grown in Luria Bertani (LB) broth at 37°C or 30°C (for microscopy) under steady agitation in the presence of inducer where appropriate (see S1 Table). Strain 4277 was grown in the presence of 20 mM MgCl_2_. Strain HM1365 was grown in the presence of selection marker. *S*. *aureus* was grown in LB at 37°C. *S*. *pneumoniae* was grown in Todd-Hewitt broth supplemented with 0.5% yeast extract at 37°C. All experiments were performed in early exponential growth phase (OD_600_ = 0.3–0.35). Unless otherwise stated, experiments were carried out in biological triplicates.

### Strain construction

Strain UG-10 (*amyE*::*spc Pxyl-recA-mgfp*) was constructed by restriction cloning of the *recA* gene into the pSG1729 plasmid [71] using *Escherichia coli* Top 10 as cloning host. RecA was amplified with the primers UG03a (5’GCGCGCCTCGAGATGAGTGATCGTCAGGCAGCC3’) and UG04a (5’CGCGCGGAATTCGGATCCTGAGCCGCTTCCTGAGCCTTCTTCAAATTCGAGTTC3’) and cloned into the vector using the XhoI and EcoRI restriction sites. The resulting plasmid (TNVS-*recA*-4GS-*mgfp*) was transformed in *B*. *subtilis* 168 using a standard starvation protocol [72]. TNVS30D (*amyE*::*spc-Pxyl-mgfp-pgsA*) was constructed by restriction cloning into the pSG1729 plasmid (carrying monomeric GFP) using XhoI/EagI restriction sites. The *pgsA* gene was amplified using the primers pgsA-Fw (gggCTCGAGggctcaggaagcggctcaggatccTTTAACTTACCAAATAAAATCACACTAGCT) and pgsA-Re (cccCGGCCGttaGTTAGATGTTTTTAACGCTTCCCA). The resulting plasmid (pTNV13) was transformed to *B*. *subtilis* 168 resulting in strain TNVS30D.

### Minimal inhibitory concentration (MIC) and growth curve

MICs were determined following a modified broth microdilution method recommended in the Clinical Laboratory Standardization Institute (CLSI) guidelines. Two-fold serial dilutions of rhodomyrtone were prepared in LB in 96-well microtiter plate format. Exponentially growing *B*. *subtilis* 168 was inoculated to each well to a final colony forming unit (CFU) count of 5x10^5^ CFU/ml and incubated at 37°C for 16 h. The MIC was defined as the lowest concentration inhibiting visible growth.

Growth experiments were performed with a Biotek Synergy MX plate reader in 96-well format under continuous shaking in a final volume of 150 μl per well. *B*. *subtilis* 168 was grown in LB at 37°C until an OD_600_ of 0.3 and subsequently treated with 0.25, 0.5, and 1 μg/ml rhodomyrtone (0.5x, 1x, and 2x MIC), or left untreated as control.

### Localization of fluorescent protein fusions

Strains expressing fluorescent protein fusions were grown at 30°C in the presence of appropriate inducer concentrations (S1 Table) until an OD_600_ of 0.3. Cells were subsequently treated with 0.5 μg/ml (1x MIC) rhodomyrtone or 1% DMSO (negative control). Unless otherwise stated, samples for microscopy were withdrawn after 10 and 30 min. Cells were immobilized on a thin film of 1% agarose and immediately observed using an Olympus BX 50 microscope equipped with a Photometrics CoolSNAP fx digital camera. Images were analyzed with Image J.

### Time-lapse microscopy

*B*. *subtilis* HS63 (*divIVA*-*msfgfp*) was grown as described above. Cells were mounted on 1% agarose patches containing 10% LB and were placed into a pre-warmed flow chamber (Ibidi sticky-Slide VI 0.4). Cells were kept at 30°C in a constant medium flow (10% LB, 20 μl/minute) and allowed to adjust for 10 min. After taking a picture of the untreated cells, rhodomyrtone, diluted in 10% LB to a final concentration of 0.5 μg/ml (1x MIC), was fed into the chamber with a continuous flow of 20 μl/min. Images were acquired every 2 min over the course of 30 min. Images were taken with a Nikon Eclipse Ti microscope equipped with a CFI Plan Apochromat DM 100x oil objective, an Intensilight HG 130 W lamp, a C11440-22CU Hamamatsu ORCA camera, and NIS elements software, version 4.20.01. Images were analyzed with Image J.

### Cell wall integrity assay

Exponentially growing (37°C) *B*. *subtilis* 168 cells were treated with 0.5 μg/ml (1x MIC) or 1 μg/ml (2x MIC) rhodomyrtone, 1 μg/ml gramicidin S, 1 μg/ml daptomycin (in the presence of 1.25 mM CaCl_2_), or 10 μg/ml MP196 for 10 min. 200 μl of sample were withdrawn and mixed with 1 ml 1:3 acetic acid methanol. This organic fixation leads to extrusion of the protoplast through cell wall holes, which occur when lipid II synthesis is inhibited while cell wall autolysins are still active [26]. Cells were mounted on 1% agarose films and observed with phase contrast microscopy using a Nikon Eclipse Ti microscope as specified above.

### Membrane potential measurements

The membrane potential was determined with the potentiometric fluorescent probe 3,3′-dipropylthiadicarbocyanine iodide (DiSC_3_(5)) using a Biotek Synergy MX plate reader as described before [28]. In short, 1 μM DiSC_3_(5) was added to exponentially growing (37°C) *B*. *subtilis* 168 cultures and the baseline was recorded for 5 min (651 nm excitation, 675 nm emission). Compounds were added (0.5 μg/ml (1x MIC), 1 μg/ml (2x MIC) rhodomyrtone, 1 μg/ml gramicidin (1x MIC), 1% DMSO) and samples were measured for another 25 min (37°C, shaking).

### Propidium iodide (PI) pore stain

Exponentially growing (37°C) *B*. *subtilis* 168 cultures were treated with 0.5 μg/ml (1x MIC), 1 μg/ml (2x MIC), 2 μg/ml (4x MIC) rhodomyrtone, 0.5% SDS (positive control), or 1% DMSO (negative control). Samples were withdrawn after 5, 10, 15, or 30 min and stained with 10 μg/ml PI for 5 min in the dark (37°C, shaking). Cells were washed twice with pre-warmed phosphate buffered saline (PBS) and resuspended in the same buffer. PI fluorescence was measured using a Biotek Synergy MX plate reader (535 nm excitation, 617 nm emission).

### Potassium leakage

*B*. *subtilis* 168 was grown in Belitzky minimal medium (BMM) at 37°C under steady agitation until early log phase. Cells were stained with 5 μM Asante Potassium Green-2 (APG-2) for 60 min in the dark (37°C, shaking). Samples were washed twice with pre-warmed BMM and resuspended in the same medium. Cells were transferred to black polystyrene 96-well plates and the baseline was recorded for 10 min (37°C, shaking) using a Biotek Synergy MX plate reader (488 nm excitation, 540 nm emission). Subsequently, antibiotics were added (0.5 μg/ml (1x MIC), 1 μg/ml (2x MIC), and 2 μg/ml (4x MIC) rhodomyrtone, 1 μg/ml (1x MIC) and 2 μg/ml (2x MIC) gramicidin (positive control), 1% DMSO (negative control)) and fluorescence was measured for additional 20 min (37°C, shaking).

### Resazurin assay

The reductive capacity of *B*. *subtilis* 168 cells, indicative of respiratory chain activity, was measured with resazurin as described before [19]. Exponentially growing cells (37°C) were treated with 0.25 μg/ml (0.5x MIC), 0.5 μg/ml (1x MIC), and 1 μg/ml (2x MIC) of rhodomyrtone, 100 μM CCCP, or 150 μM sodium azide. Aliquots were withdrawn after 10 and 30 min and adjusted to an OD_600_ of 0.15 with pre-warmed LB and 100 μg/ml resazurin was added. After 5 min incubation (37°C, shaking) the optical density was measured at 540 and 630 nm using a Biotek Synergy MX plate reader.

### Membrane staining

All membrane-specific experiments were performed at 30°C. Unless otherwise stated in the figure legends, exponentially growing cells were treated with 0.5 μg/ml rhodomyrtone (1x MIC) for 10 min. Membranes were stained with 2 μg/ml FM5-95 (Molecular Probes) for 10 min immediately prior to microscopy. Nucleoids were stained with 1 μg/ml DAPI (Thermo Scientific) for 2 min immediately prior to microscopy. For RIF staining with DiIC12 (Anaspec) overnight cultures were diluted 1:200 in LB containing 1% DMSO, 2 μg/ml DiIC12. Cells were washed four times and resuspended in pre-warmed LB containing 1% DMSO, prior to antibiotic treatment. Laurdan (Sigma Aldrich) microscopy was performed as described previously [19]. Analysis of laurdan microscopy images was performed with Image J using the ‘calculate GP’ plugin. GP calculation from microscopy pictures requires the following steps: correction of the image size, brightness and contrast enhancement, background subtraction, and GP calculation. Since the brightness and contrast adjustments must be the same for both pictures, cells are typically surrounded by a certain fluorescence background. Similarly, intracellular background fluorescence results in GP values in the cytosol of the cells, which are not representative of membrane fluidity. To clearly identify the membrane in these images, black and grey GP images were overlaid with the 460 nm fluorescence image (red). All microscopy was performed using the Nikon Eclipse Ti as specified above.

### Preparation of liposomes

Liposomes were either prepared from *Escherichia coli* polar lipid extract, POPG, or POPC (Avanti Polar Lipids) using a detergent-dialysis method described before [24] and extruded 20 times through 0.4 μm (spectroscopy) or 0.8 μm (microscopy) filters. Concentrated (10 mg/ml) liposome stock solutions were diluted to 1 mg/ml working solutions in 5 mM Tris, pH 7.4 (spectroscopy) or 50 mM Tris, pH 7.4 (microscopy). Liposomes were stained with 10 μM laurdan for 30 min or with 1 μg/ml DiIC12 for 2 min. Liposome samples were kept at 30°C during all experiments. Compound to lipid ratios *in vivo* were calculated based on lipid per cell data published for *E*. *coli* (2.2x10^7^ lipids per cell) [73], using our standard treatment conditions (OD_600_ of 0.3, 0.5 μg/ml rhodomyrtone) considering that a *B*. *subtilis* cell is twice as long as an *E*. *coli* cell and assuming that all rhodomyrtone would be bound to the membrane. This calculation resulted in an *in vivo* compound to lipid ratio of 1:20. However, since it is unknown how much rhodomyrtone is bound to the membrane, free in the medium, or sequestered in the *B*. *subtilis* cell wall, this number can only be considered a rough estimate of the magnitude of the *in vivo* compound to lipid ratio.

### Laurdan spectroscopy

Membrane fluidity was determined in batch culture with laurdan as described before [19]. Cells were grown until mid-log phase, stained with 10 μM laurdan, washed four times in PBS supplemented with 2% glucose and 1% DMF, and resuspended in the same buffer to give an OD_600_ of 0.3. Liposomes were stained with 10 μM laurdan for 30 min. Laurdan fluorescence was measured in a BioTek Synergy MX plate reader using 350 nm excitation and 460 and 500 nm emission wavelengths. Readings were taken every 2 min. Laurdan generalized polarization (GP) values were calculated with the following formula: (I_460_-I_500_)/(I_460_+I_500_). Antibiotics were added to cells or liposomes after 4 min of measurements and fluorescence was further measured over 30 min.

### Lipid analysis

Overnight cultures were diluted 1:100 and grown at 30°C under steady agitation. At an OD_600_ of 0.3, cultures were split and 500 ml were treated with 0.25 μg/ml (0.5x MIC) rhodomyrtone or 1% DMSO as negative control. Lower concentrations of rhodomyrtone were necessary for these experiments because of different growth rates of *B*. *subtilis* in large cell culture flasks compared to microtiter plates due to different aeration (S23 Fig). Cells were further grown to an OD_600_ of 0.6 and subsequently chilled on slush ice for 10 min. Cells were harvested by centrifugation and washed once in ice-cold 100 mM NaCl. Washed cell pellets were immediately flash-frozen in liquid nitrogen and lyophilized at -50°C overnight. Freeze-dried pellets were sonicated and dissolved in hexane prior to analysis by gas chromatography-mass spectrometry (GC-MS) as described by Medema *et al*. [74]. In short, samples were taken up in 1 ml transmethylation reagent (3 M HCl in methanol) and incubated in the presence of 10 nmol internal standard (the methyl ester of 18-methylnonadecanoic acid) at 90°C for 4 hours. After cooling, the aqueous layer was extracted with 2 ml hexane, dried under nitrogen flow and resuspended in 80 μl hexane. One microliter of this solution was injected into a gas chromatograph (Hewlett Packard GC 5890) equipped with an Agilent J&W HP-FFAP, 25m, 0.20mm, 0.33μm GC Column. Eluting fatty acid methyl esters were detected by flame ionization. Fatty acid concentrations were calculated using the known amount of internal standard and expressed as percentage of the total amount of lipids. Experiments were performed in biological duplicates.

### Structured illumination microscopy (SIM)

3D SIM was performed using a Nikon Eclipse Ti N-SIM E microscope setup equipped with a CFI SR Apochromat TIRF 100x oil objective (NA1.49), a LU-N3-SIM laser unit, an Orca-Flash 4.0 sCMOS camera (Hamamatsu Photonics K.K.), and NIS elements Ar software. Cultures were grown and mounted on microscopy slides as described above. Cell membranes were stained with 1 μg/ml mitotracker green or 1 μg/ml nile red for 2 min. Liposomes were stained with 1 μg/ml DiIC12. For observation of samples stained with nile red or DiIC12 coverslips were coated with poly-dopamine as described earlier [28].

### Transmission electron microscopy

*B*. *subtilis* 168 was aerobically grown in LB until an OD_600_ of 0.3 and subsequently treated with 0.5 μg/ml (1x MIC) rhodomyrtone or left untreated as control. After 10 min of antibiotic treatment, 200 μl of sample were withdrawn, pelleted by centrifugation (16,000x g, 1 min), and resuspended in 10 μl fresh LB. Concentrated cell suspensions were spotted on 0.25 mm thick (Gene Frame AB0576, ThermoFischer Scientific) 1.5% agarose patches, and allowed to dry. Agarose patches were transferred to aluminum dishes and cells were fixed with 5% glutaraldehyde in 0.1 M cacodylate buffer (pH 7.4) for 20 min. After fixation, samples were washed three times with 0.1 M cacodylate, pH 7.4 for 5 min each, followed by incubation in 1% OsO_4_ (EMS) / 1% KRu(III)(CN)_6_ (Sigma Aldrich) for 30 min and three times washing with ultrapure water for 5 min each. Dehydration was performed in a series of incubation steps with rising concentrations of ultrapure ethanol as follows: 5 min 30% ethanol, 5 min 50% ethanol, twice 15 min 70% ethanol, 1 h 80% ethanol, 15 min 90% ethanol, 15 min 96% ethanol, 15 min 100% ethanol, 30 min 100% ethanol, water-free. Cells were then incubated for 30 min in a 1:1 mixture of EPON and propylene oxide, followed by 30 min incubation in 2:1 EPON / propylene oxide. Agarose patches were then transferred to fresh aluminum dishes, covered with fresh EPON, and incubated at 65°C for 48 h. After embedding, a region of interest was selected by observing the EPON-embedded bacterial layer under a light microscope and mounted for thin sectioning. Ultrathin sections (~80 nm) were cut parallel to the bacterial layer, collected on single-slot, Formvar-coated copper grids, and subsequently counterstained with uranyl acetate (Ultrostain I, laurylab) and lead citrate (Reynolds) in a Leica EM AC20 ultrastainer. Bacteria were imaged at 6000x magnification using a JEOL 1010 transmission electron microscope at an electron voltage of 60 kV using a side-mounted CCD camera (Modera, EMSIS).

### Molecular dynamics simulations

To mimic the *B*. *subtilis* membrane, a phospholipid bilayer composed of 3:1 1-palmitoyl-2-oleoyl-phosphatidylglycerol (POPG) and 1-palmitoyl-2-oleoylphosphatidylethanolamine (POPE) was used [75,76]. Two membrane systems were created, one with (S)- and one with (R)-rhodomyrtone. The bilayer system contained a total of 60 lipids in each leaflet and about 4000 water molecules were built. Sodium ions were added to neutralize the negatively charged head groups of each POPG molecule. Either (R)- or (S)-rhodomyrtone was added randomly. All system preparations were simultaneously performed using Packmol software [77]. The dimension size was in a rectangular box of 60 Å × 60 Å × 120 Å. All simulations were subjected to 1000 steps using the steepest descent algorithm, followed by 1000 steps of conjugate gradient minimization. To equilibrate the system, 0.5 ns NVT and 5.5 ns NPT simulations were performed at 310 K and 1 atm. Production simulations (NPT) for each lipid system were then conducted in the NPT ensemble at 310 K and 1 atm for 220 ns. All simulations used periodic boundary conditions, particle mesh Ewald with a 10 A° cut-off, and the SHAKE algorithm to constrain the bonds containing hydrogen atoms. All MD simulations were carried out with PMEMD implemented in AMBER16 package. The first 120 ns simulation was omitted and the last 1000 equidistant snapshots from 100 ns simulation were taken for an average and analysis. The analysis was carried out using the CPPTRAJ module in AMBER16 package and custom Fortran 95 written programs. Visualization was operated using Visual Molecular Dynamics (VMD 1.9.3) suite [78]. Probability per lipid of lipid type interacting with rhodomyrtone was calculated as follows:

$$\text{Probability}\;\text{per}\;\text{lipid}=\frac{\frac{\text{Number}\;\text{of}\;\text{frames}\;\text{of}\;\text{lipid}\;\text{interacting}\;\text{with}\;\text{rhodomyrtone}}{\text{Number}\;\text{of}\;\text{total}\;\text{simulated}\;\text{frames}}}{\text{Number}\;\text{of}\;\text{lipids}\;\text{in}\;\text{the}\;\text{membrane}\;\text{structure}}$$

### Zebrafish embryo experiments

All methods were carried out in accordance with relevant guidelines and regulations. *Danio rerio* (zebrafish) were handled in compliance with the local animal welfare regulations and maintained according to standard protocols (zfin.org). The breeding of zebrafish in authorized institutions such as the Amsterdam Animal Research Center of the VU University Amsterdam is in full compliance with the Dutch law on animal research. All animal experiments are supervised by the local Animal Welfare Body (Instantie voor Dierenwelzijn, IvD) of the VU University and the VU University Medical Center (IvD VU/VUmc). All used research protocols adhere to the international guidelines on the protection of animals used for scientific purposes, the EU Animal Protection Directive 2010/63/EU, which allows zebrafish embryos to be used up to the moment that they are able to independently take up external food (5 days after fertilization) without additional approval by the Central Committee for Animal Experiments in the Netherlands (Centrale Commissie Dierproeven, CCD). Because the zebrafish embryos used in this study meet these criteria, this specific study was therefore approved by the IvD VU/VUmc. *Casper* zebrafish embryos were infected at 1 day post fertilization in the caudal vein by microinjection with green fluorescent Streptococcus pneumoniae D39 strain, in which the superfolder green fluorescent protein is fused to HlpA, as previously described [56,79]. The embryos were treated 45 min after infection either by microinjection of rhodomyrtone into the caudal vein or by addition of the compound to the water at the indicated concentrations. Infected embryos were monitored at specific time-points with a Leica MZ16FA fluorescence microscope attached with a Leica DFC420C camera. All experiments were performed in triplicate.

### Activity against non-growing cells

*B*. *subtilis* 168 was grown over night at 37°C. Stationary phase cultures were then incubated with 4 or 40 μg/ml of rhodomyrtone or gramicidin S, respectively, for 9 h at 37°C under continuous shaking. CFU counts were determined by plating dilution series in LB plates without antibiotics.

## Supporting information

S1 Table *B*. *subtilis* strains used in this study. *gfp*: green-fluorescent protein, *mgfp*: monomeric *gfp*; *sfgfp*: superfolder *gfp*, *msfgfp*: monomeric superfolder *gfp; yfp*: yellow-fluorescent protein.
(DOCX)

S2 Table Adaptation of the fatty acid composition of membrane lipids. *B*. *subtilis* 168 was grown until an OD_600_ of 0.3 and treated with 0.25 μg/ml rhodomyrtone (0.5x MIC, sub-inhibitory). Cells were harvested after reaching an OD_600_ of 0.6. dev: standard deviation of the mean.
(DOCX)

S3 Table Raw data and analysis of fatty acid analysis. *B*. *subtilis* 168 was grown until an OD_600_ of 0.3 and treated with 0.25 μg/ml rhodomyrtone (0.5x MIC). Cells were harvested after reaching an OD_600_ of 0.6. dev: standard deviation.
(DOCX)

S4 Table Frequency of lipid interactions with rhodomyrtone during the last 100 ns MD simulation trajectories (1000 frames). (DOCX)

S5 Table Minimal inhibitory concentrations of different antibiotics against the PG depletion strain HM1365 compared to *B*. *subtilis* WT. Depletion of essential PG lipids leads to growth defects and morphological changes^15^ resulting in antibiotic hypersensitivity (**bold**) in *B*. *subtilis*. Daptomycin is an exception to this rule, since its activity depends on the presence of PG leading to higher daptomycin tolerance of the PG depletion strain.
(DOCX)

S1 Fig Delocalization of intracellular proteins by specific inhibitors. Cells were treated with antibiotics for 10 min (0.1 μg/ml ciprofloxacin, 0.1 μg/ml rifampicin, 0.2 μg/ml erythromycin) or 60 min (0.05 μg/ml mitomycin C) in mid-log phase. Scale bar 2 μm.
(TIF)

S2 Fig Overview of untreated *B*. *subtilis* cells expressing GFP-MreB. Scale bar 5 μm.
(TIF)

S3 Fig Overview of rhodomyrtone-treated *B*. *subtilis* cells expressing GFP-MreB. Cells were treated with 1xMIC for 10 min. Arrows indicate some of the MreB accumulations. Scale bar 5 μm.
(TIF)

S4 Fig Depolarization measured with DiSC(3)5 over 25 min. Arrow indicates time point of antibiotic addition.
(TIF)

S5 Fig Overview of FM5-95 stained control cells. Scale bar 5 μm.
(TIF)

S6 Fig Overview of FM5-95 stained cells treated with rhodomyrtone. Cell were treated with 1x MIC for 10 min. Arrows indicate some of the FM5-95 patches. Scale bar 5 μm.
(TIF)

S7 Fig Overview of DAPI-stained control cells. Scale bar 5 μm.
(TIF)

S8 Fig Overview of DAPI-stained rhodomyrtone-treated cells. Cells were treated with 1x MIC for 10 min. Note the heterogeneity of the DAPI stain due to increased membrane permeability in severely affected cells. Scale bar 5 μm.
(TIF)

S9 Fig Growth phase-dependent formation of visible DilC12-stained RIFs. *B*. *subtilis* 168 was aerobically grown in LB at 30°C. Discrete RIFS become visible during logarithmic growth and disappear upon entry into stationary phase.
(TIF)

S10 Fig Overview of DiIC12-stained control cells. Scale bar 5 μm.
(TIF)

S11 Fig Overview of DiIC12-stained cells treated with rhodomyrtone. Cells were treated with 1x MIC for 10 min. Arrows indicate some of the DiIC12 patches. Scale bar 5 μm.
(TIF)

S12 Fig Laurdan partitions into fluid membrane domains. Fluorescence intensity was measured in 460 nm laurdan fluorescence images. Error bars represent standard error of the mean.
(TIF)

S13 Fig Growth arrest does not cause membrane fluidization. *B*. *subtilis* 168 was treated with a bactericidal concentration of ciprofloxacin (1 μg/ml) for 10 min prior to spectroscopic **(A)** or microscopic **(B)** fluidity measurements with laurdan.
(TIF)

S14 Fig *B*. *subtilis* cells expressing AtpA-GFP stained with FM5-95. AtpA clearly accumulated in FM5-95-stained membrane domains.
(TIF)

S15 Fig Inhibition of either protein synthesis or lipid synthesis does not block formation of membrane patches. Cells were pre-treated with 100 μg/ml chloramphenicol (left panels) or 2.5 μg/ml triclosan (right panels) for 10 min to inhibit synthesis of proteins and lipids, respectively. Subsequently, rhodomyrtone was added and pictures were taken after additional 10 min. Membranes were stained with FM5-95.
(TIF)

S16 Fig Membrane proteins do not relocate to their normal localization up to 1 h after addition of compound. *B*. *subtilis* TNVS284 (*mraY-gfp*) was treated with 1x MIC of rhodomyrtone for 60 min. Membranes were stained with FM5-95. Red boxes indicate MraY trapped in membrane invaginations caused by rhodomyrtone. Scale bar 2 μm.
(TIF)

S17 Fig Molecular dynamics simulations with multiple rhodomyrtone molecules. Simulations were run with one **(A)**, two **(B)**, four **(C)**, or eight **(D)** rhodomyrtone molecules. Snapshots show aggregated rhodomyrtone molecules interacting with the phospholipid head groups. Arrows indicate bilayer disturbance in terms of fatty acyl chain spreading.
(TIF)

S18 Fig Low concentrations of rhodomyrtone can be washed from cells. *B*. *subtilis* 168 was treated with rhodomyrtone for 2 or 10 min, respectively, and subsequently washed twice with pre-warmed LB medium. Cells were then allowed to grow for 1 h and examined under the microscope. Membranes were stained with FM5-95. Arrows indicate membrane patches caused by rhodomyrtone. Scale bar 2 μm.
(TIF)

S19 Fig Effects of rhodomyrtone on zebrafish embryos infected with *S*. *pneumoniae*. **(A)** Rhodomyrtone-treated fish consistently showed less green fluorescence caused by infection with a GFP-expressing strain of *S*. *pneumoniae*. **(B)** Rhodomyrtone prevents damage to the heart region caused by *S*. *pneumoniae* infection (red arrows). Damage to the heart region was observed in 80% of untreated and 30% of rhodomyrtone-treated fish. One day old zebra fish embryos were injected with 160 CFU of *S*. *pneumoniae* JWV500 expressing HlpA-GFP in the tail vein. Fish were treated with two injections (45 and 75 min post infection) of 25 ng rhodomyrtone each. Pictures were taken 18 hours post infection. Experiments were performed in biological triplicates with a minimum of 15 fish per condition in each replicate.
(TIF)

S20 Fig Effect of rhodomyrtone on human erythrocytes. Fresh blood from a healthy donor was stained with 16 μg/ml DiIC12 for 10 min and subsequently treated with rhodomyrtone for 10 additional minutes prior to inspection by fluorescence light microscopy. Scale bar 10 μm.
(TIF)

S21 Fig Fluidizing effect of rhodomyrtone on POPG and POPC liposomes. PG is one of the main membrane lipid species in bacteria but only rarely present in mammalian cells, while PC is the major component of mammalian membranes but absent in most bacterial membranes. Green: untreated. Blue: 50 μg/ml rhodomyrtone (compound to lipid ratio 1:7).
(TIF)

S22 Fig Activity of rhodomyrtone against non-growing (overnight) cultures of *B*. *subtilis* 168. Stationary phase cells were treated with compounds for 9 h prior to plating on non-selective LB agar plates. Gramicidin S, which is known to kill persister cells^16^, was used as control.
(TIF)

S23 Fig Growth of cultures used for fatty acid analysis. In contrast to growth experiments in 96-well microtiter plates (Fig 3A), 1x MIC led to complete growth inhibition in this experiment (500 ml shaking cultures in 3 L flasks) due to different oxygen supply and subsequent differences in growth rate. Therefore, 0.5x MIC was used for fatty acid analysis.
(TIF)

S1 Movie Time lapse microscopy of *B*. *subtilis* HS63 expressing DivIVA-GFP treated with 0.5 μg/ml rhodomyrtone (1x MIC). Pictures were taken every 2 min. Scale bar 5 μm.
(AVI)

S2 Movie Molecular dynamics simulations of the interaction of rhodomyrtone with POPG/POPE (3:1) membranes (100 ns). Rhodomyrtone (orange) transiently interacts with both POPG (blue) and POPE (green).
(7Z) Click here for additional data file.

S1 References References for methods and strains used in this study. (DOCX)
