# Supplementary material for: The novel antibiotic rhodomyrtone traps membrane proteins in vesicles with increased fluidity
Source: PLoS Pathog. 2018 Feb 16;14(2):e1006876. doi: 10.1371/journal.ppat.1006876 (PMC5833292; doi:10.1371/journal.ppat.1006876)
Supplement: S1 Table — gfp: green-fluorescent protein, mgfp: monomeric gfp; sfgfp: superfolder gfp, msfgfp: monomeric superfolder gfp; yfp: yellow-fluorescent protein. (DOCX) [file ppat.1006876.s001.docx]

**Table S1:** *B. subtilis* strains used in this study. *gfp*: green-fluorescent protein, *mgfp*: monomeric *gfp*; *sfgfp*: superfolder *gfp*, *msfgfp*: monomeric superfolder *gfp; yfp:* yellow-fluorescent protein.

| **Strain** | **genotype** | **induction** | **reference** |
| --- | --- | --- | --- |
| *B. subtilis* strains | | | |
| 168 | *trpC2* | - | ^1^ |
| 1048 | *cat rpoC-gfp Pxyl-‘rpoC* | 1% xylose | ^2^ |
| 1049 | *amyE::spc Pxyl-rpsB-gfp* | 1% xylose | ^2^ |
| 4277 | *Ωneo3427 ∆mreB ∆mbl::cat ∆mreBH::erm ∆rsgI::spc* | - | ^3^ |
| BS23 | *atpA-gfp Pxyl-′atpA cat* | 0.1% xylose | ^4^ |
| BS121 | *sdhA-gfp Pxyl-‘sdhA cat* | 0.1% xylose | ^4^ |
| bSS82 | *amyE:: spc PrpsD-gfp* | - | ^5^ |
| EKB44 | *amyE::spc-Pxyl-msfgfp-pbpB* | 0.1% xylose | ^6^ |
| HS63 | *amyE::spc Pxyl-divIVA-msfgfp* | 0.5% xylose | ^5^ |
| HB5337 | *mprF::kan* | - | ^7^ |
| HB5361 | *pssA::spc* | - | ^7^ |
| HM771 | *dnaN::gfp-dnaN cat* (MS104 into 168) | - | ^8^ |
| HM1365 | *pgsA’Ω(Pxyl-pgsA cat) aprE::(Pspac-Pxyl-xylR lacI erm)* | 0.0016% xylose | ^9^ |
| LH131 | *amyE::spc Pxyl-gfp-minD* | 0.1% xylose | ^6^ |
| MW10 | *amyE::spc Pxyl-gfp-mreB* | 0.3% xylose | ^6^ |
| PG62 | *aprE::spc Pspac-yfp-ftsA* | 0.1 mM IPTG | ^10^ |
| SDB206 | *ywiE2::neo ywjE::spc clsA::pMUTIN4* | - | ^11^ |
| TNVS29D | *amyE::spc-Pxyl-mgfp-plsX* | 0.5% xylose | ^6^ |
| TNVS30D | *amyE::spc-Pxyl-mgfp-pgsA* | 0.5% xylose | this study |
| TNVS175 | *amyE::spc-Pxyl-murG-msfgfp* | 0.05% xylose | ^6^ |
| TNVS284 | *amyE::spc-Pxyl-mraY-msfgfp* | 0.1% xylose | ^6^ |
| UG-10 | *amyE::spc Pxyl-recA-mgfp* | 0.5% xylose | this study |
| other strains | | | |
| D39 | *S. pneumonaie* WT | - | ^12^ |
| JWV500 | *S. pneumoniae* D39 *hlpA-gfp_Cam^r^* | - | ^13^ |
| RN4220 | *S. aureus* WT | - | ^14^ |
|  |  |  |  |
