# Supplementary material for: The novel antibiotic rhodomyrtone traps membrane proteins in vesicles with increased fluidity
Source: PLoS Pathog. 2018 Feb 16;14(2):e1006876. doi: 10.1371/journal.ppat.1006876 (PMC5833292; doi:10.1371/journal.ppat.1006876)
Supplement: S2 Table — B. subtilis 168 was grown until an OD600 of 0.3 and treated with 0.25 μg/ml rhodomyrtone (0.5x MIC, sub-inhibitory). Cells were harvested after reaching an OD600 of 0.6. dev: standard deviation of the mean. (DOCX) [file ppat.1006876.s002.docx]

**Table S2:** Adaptation of the fatty acid composition of membrane lipids. *B. subtilis* 168 was grown until an OD_600_ of 0.3 and treated with 0.25 µg/ml rhodomyrtone (0.5x MIC, sub-inhibitory). Cells were harvested after reaching an OD_600_ of 0.6. dev: standard deviation of the mean.

|  | control 1 | control 2 | rhodomyrtone 1 | rhodomyrtone 2 | mean control | mean rhodomyrtone | dev control | dev mean |
| --- | --- | --- | --- | --- | --- | --- | --- | --- |
| saturated | 5.349 | 5.858 | 4.930 | 9.669 | 5.604 | 7.299 | 0.360 | 3.351 |
| unsaturated | 0.398 | 0.448 | 0.421 | 0.561 | 0.423 | 0.491 | 0.036 | 0.099 |
| ratio saturated / unsaturated | 13.457 | 13.066 | 11.724 | 17.232 | 13.261 | 14.478 | 0.277 | 3.894 |
|  |  |  |  |  |  |  |  |  |
| iso | 31.719 | 40.044 | 27.181 | 37.524 | 35.881 | 32.353 | 5.886 | 7.314 |
| anteiso | 44.810 | 52.491 | 36.575 | 50.784 | 48.651 | 43.680 | 5.431 | 10.047 |
| ratio iso / anteiso | 0.708 | 0.763 | 0.743 | 0.739 | 0.735 | 0.741 | 0.039 | 0.003 |
|  |  |  |  |  |  |  |  |  |
| short (<16) | 53.296 | 64.033 | 41.452 | 55.127 | 58.665 | 48.289 | 7.592 | 9.670 |
| long (>=16) | 28.980 | 34.808 | 31.861 | 46.991 | 31.894 | 39.426 | 4.121 | 10.699 |
| ratio long (≥16 C) / short (<16 C) | 0.544 | 0.544 | 0.769 | 0.852 | 0.544 | 0.811 | 0.000 | 0.059 |
|  |  |  |  |  |  |  |  |  |
| branched | 76.529 | 92.535 | 63.756 | 88.309 | 84.532 | 76.033 | 11.318 | 17.361 |
| non-branched | 5.747 | 6.307 | 5.351 | 10.230 | 6.027 | 7.790 | 0.396 | 3.450 |
| ratio non-branched / branched | 0.075 | 0.068 | 0.084 | 0.116 | 0.072 | 0.100 | 0.005 | 0.023 |
