# Supplementary material for: The novel antibiotic rhodomyrtone traps membrane proteins in vesicles with increased fluidity
Source: PLoS Pathog. 2018 Feb 16;14(2):e1006876. doi: 10.1371/journal.ppat.1006876 (PMC5833292; doi:10.1371/journal.ppat.1006876)
Supplement: S3 Table — B. subtilis 168 was grown until an OD600 of 0.3 and treated with 0.25 μg/ml rhodomyrtone (0.5x MIC). Cells were harvested after reaching an OD600 of 0.6. dev: standard deviation. (DOCX) [file ppat.1006876.s003.docx]

**Table S3:** Raw data and analysis of fatty acid analysis. *B. subtilis* 168 was grown until an OD_600_ of 0.3 and treated with 0.25 µg/ml rhodomyrtone (0.5x MIC). Cells were harvested after reaching an OD_600_ of 0.6. dev: standard deviation.

| fatty acid species | branching | chain length | saturation | control 1 | control 2 | rhodomyrtone 1 | rhodomyrtone 2 | ratio 1 | ratio 2 | ratio mean | ratio dev |
| --- | --- | --- | --- | --- | --- | --- | --- | --- | --- | --- | --- |
|  |  |  |  |  |  |  |  |  |  |  |  |
| 11 me C12:0 | iso | short | saturated | 0.111 | 0.161 | 0.100 | 0.124 | 0.900 | 0.770 | 0.835 | 0.092 |
| 10 me C12:0 | anteiso | short | saturated | 0.139 | 0.164 | 0.127 | 0.135 | 0.915 | 0.823 | 0.869 | 0.065 |
| 12me C13:0 | iso | short | saturated | 0.895 | 1.135 | 0.713 | 1.901 | 0.797 | 1.675 | 1.236 | 0.621 |
| C14:0 | non-branched | short | saturated | 0.319 | 0.344 | 0.311 | 0.512 | 0.975 | 1.490 | 1.233 | 0.364 |
| 13me C14:0 | iso | short | saturated | 18.292 | 22.904 | 15.439 | 17.597 | 0.844 | 0.768 | 0.806 | 0.054 |
| 12me C14:0 | anteiso | short | saturated | 33.541 | 39.326 | 28.629 | 40.000 | 0.854 | 1.017 | 0.935 | 0.116 |
| C15:0 | non-branched | short | saturated | 0.178 | 0.146 | 0.102 | 0.254 | 0.575 | 1.745 | 1.160 | 0.827 |
| 14 me C15:0 | iso | short | saturated | 3.365 | 4.127 | 3.153 | 7.891 | 0.937 | 1.912 | 1.425 | 0.690 |
| C16:0 | non-branched | long | saturated | 3.988 | 4.179 | 3.934 | 7.840 | 0.986 | 1.876 | 1.431 | 0.629 |
| C16:1w9 | non-branched | long | saturated | 0.010 | 0.010 | 0.008 | 0.010 | 0.796 | 0.957 | 0.876 | 0.114 |
| C16:1w7 | non-branched | long | saturated | 0.073 | 0.088 | 0.082 | 0.140 | 1.112 | 1.596 | 1.354 | 0.343 |
| 15 me C16:0 | iso | long | saturated | 8.993 | 11.636 | 10.192 | 13.198 | 1.133 | 1.134 | 1.134 | 0.001 |
| 14 me C16:0 | anteiso | long | saturated | 11.131 | 13.001 | 11.232 | 15.387 | 1.009 | 1.184 | 1.096 | 0.123 |
| C17:0 | non-branched | long | saturated | 0.069 | 0.059 | 0.068 | 0.167 | 0.997 | 2.821 | 1.909 | 1.290 |
| 16 me C17:0 | iso | long | saturated | 0.063 | 0.081 | 0.119 | 0.313 | 1.901 | 3.840 | 2.871 | 1.371 |
| C18:0 | non-branched | long | saturated | 0.796 | 1.131 | 0.975 | 1.798 | 1.224 | 1.590 | 1.407 | 0.259 |
| C18:1w9 | non-branched | long | unsaturated | 0.048 | 0.065 | 0.047 | 0.085 | 0.982 | 1.308 | 1.145 | 0.231 |
| fatty acid species | branching | chain length | saturation | control 1 | control 2 | rhodomyrtone 1 | rhodomyrtone 2 | ratio 1 | ratio 2 | ratio mean | ratio dev |
| C18:1w7 | non-branched | long | unsaturated | 0.009 | 0.010 | 0.009 | 0.012 | 1.038 | 1.213 | 1.125 | 0.124 |
| C18:2w6 | non-branched | long | unsaturated | 0.065 | 0.081 | 0.138 | 0.216 | 2.123 | 2.652 | 2.387 | 0.374 |
| C18:3w6 | non-branched | long | unsaturated | 0.009 | 0.009 | 0.011 | 0.014 | 1.275 | 1.566 | 1.421 | 0.206 |
| C20:3w6 | non-branched | long | unsaturated | 0.040 | 0.038 | 0.038 | 0.032 | 0.938 | 0.858 | 0.898 | 0.056 |
| C22:5w3 | non-branched | long | unsaturated | 0.123 | 0.125 | 0.097 | 0.084 | 0.789 | 0.671 | 0.730 | 0.083 |
