# Supplementary material for: The novel antibiotic rhodomyrtone traps membrane proteins in vesicles with increased fluidity
Source: PLoS Pathog. 2018 Feb 16;14(2):e1006876. doi: 10.1371/journal.ppat.1006876 (PMC5833292; doi:10.1371/journal.ppat.1006876)
Supplement: S4 Table — (DOCX) [file ppat.1006876.s004.docx]

**Table S4:** Frequency of lipid interactions with rhodomyrtone during the last 100 ns MD simulation trajectories (1000 frames).

|  | Number of frames of lipids | | Probability per lipid type interacting with rhodomyrtone | |
| --- | --- | --- | --- | --- |
|  | POPE | POPG | POPE (15 lipids) | POPG (45 lipids) |
| (R)-rhodomyrtone | 122 | 240 | 0.0081 | 0.0053 |
| (S)-rhodomyrtone | 153 | 343 | 0.0102 | 0.0076 |
