# Supplementary material for: The novel antibiotic rhodomyrtone traps membrane proteins in vesicles with increased fluidity
Source: PLoS Pathog. 2018 Feb 16;14(2):e1006876. doi: 10.1371/journal.ppat.1006876 (PMC5833292; doi:10.1371/journal.ppat.1006876)
Supplement: S5 Table — Depletion of essential PG lipids leads to growth defects and morphological changes15 resulting in antibiotic hypersensitivity (bold) in B. subtilis. Daptomycin is an exception to this rule, since its activity depends on the presence of PG leading to higher daptomycin tolerance of the PG depletion strain. (DOCX) [file ppat.1006876.s005.docx]

**Table S5:** Minimal inhibitory concentrations of different antibiotics against the PG depletion strain HM1365 compared to *B. subtilis* WT. Depletion of essential PG lipids leads to growth defects and morphological changes^15^ resulting in antibiotic hypersensitivity (**bold**) in *B. subtilis*. Daptomycin is an exception to this rule, since its activity depends on the presence of PG leading to higher daptomycin tolerance of the PG depletion strain.

| antibiotic compound | Minimal inhibitory concentration (µg/ml) | |
| --- | --- | --- |
|  | 168 WT | HM1365 PG depletion |
| rhodomyrtone | 0.5 | **0.1** |
| daptomycin | 1 | 2 |
| vancomycin | 0.5 | **0.25** |
| ampicillin | 0.5 | **0.0625** |
| gramicidin | 1 | **0.0625** |
| cephalexin | 0.5 | **0.0625** |
| nitrofurantoin | 8 | **4** |
| tetracycline | 8 | **1.5** |
