# Supplementary figures and images for: The novel antibiotic rhodomyrtone traps membrane proteins in vesicles with increased fluidity
Source: PLoS Pathog. 2018 Feb 16;14(2):e1006876. doi: 10.1371/journal.ppat.1006876 (PMC5833292; doi:10.1371/journal.ppat.1006876)

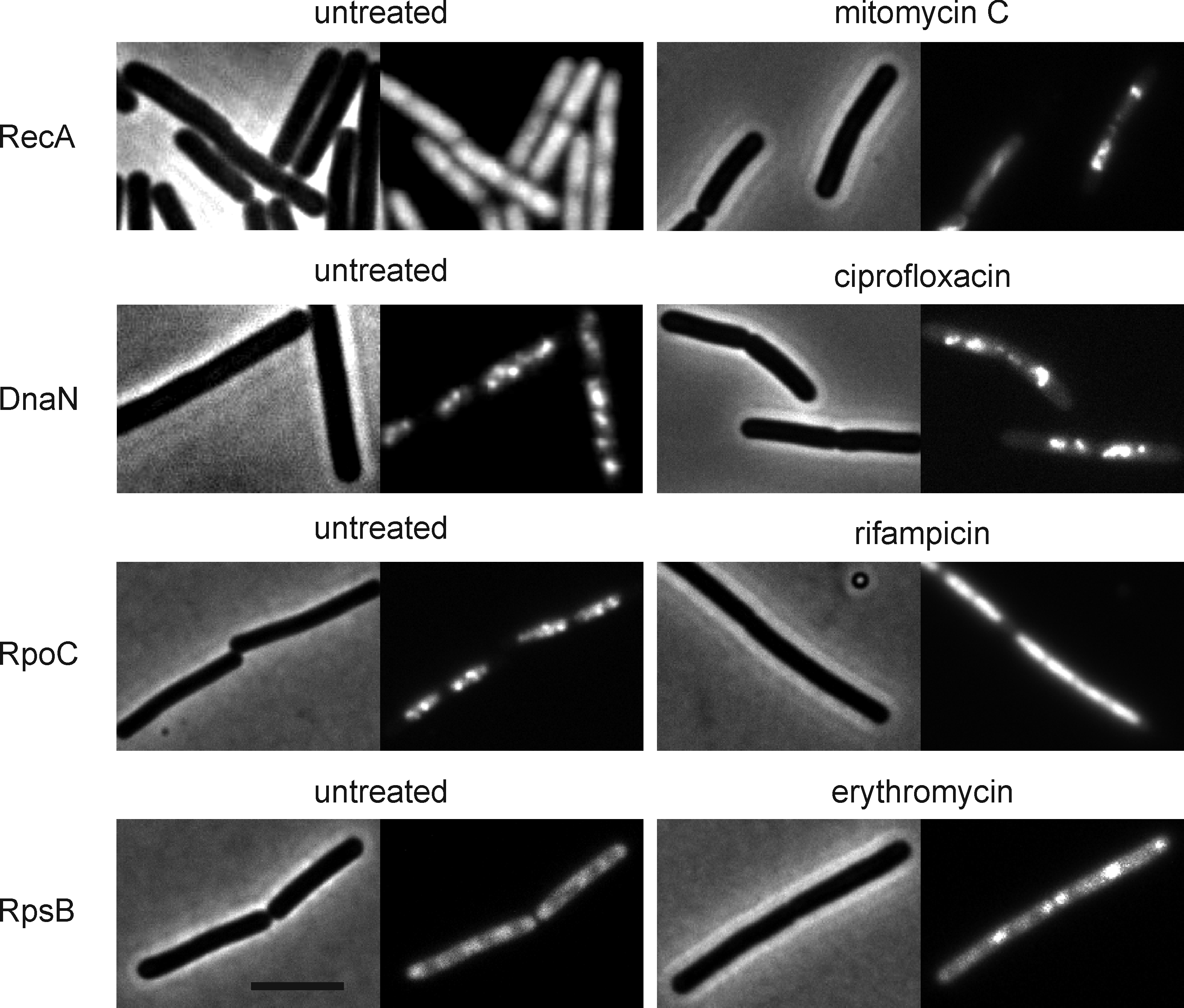

Supplement: S1 Fig — Cells were treated with antibiotics for 10 min (0.1 μg/ml ciprofloxacin, 0.1 μg/ml rifampicin, 0.2 μg/ml erythromycin) or 60 min (0.05 μg/ml mitomycin C) in mid-log phase. Scale bar 2 μm. (TIF) [file ppat.1006876.s006.tif]

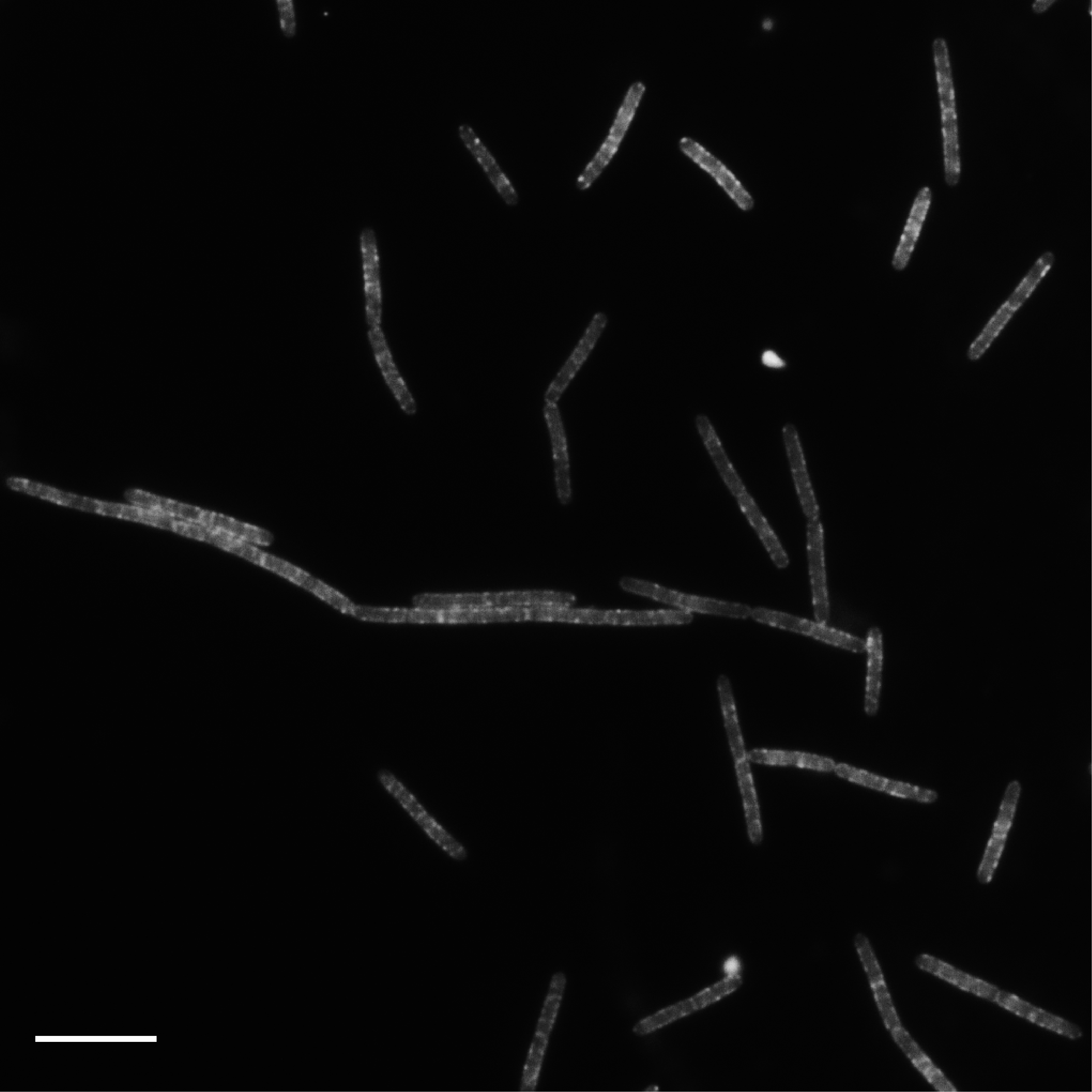

Supplement: S2 Fig — Scale bar 5 μm. (TIF) [file ppat.1006876.s007.tif]

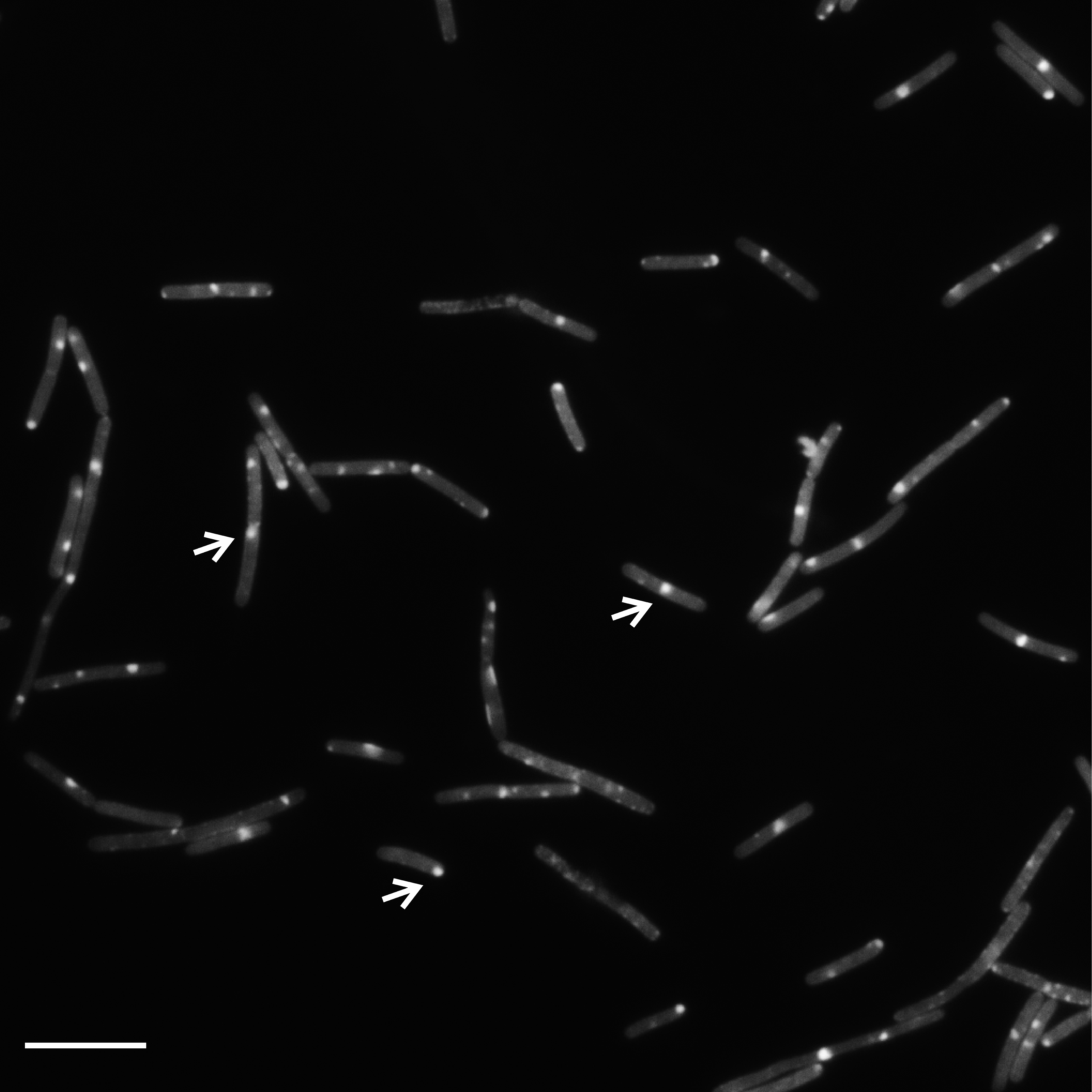

Supplement: S3 Fig — Cells were treated with 1xMIC for 10 min. Arrows indicate some of the MreB accumulations. Scale bar 5 μm. (TIF) [file ppat.1006876.s008.tif]

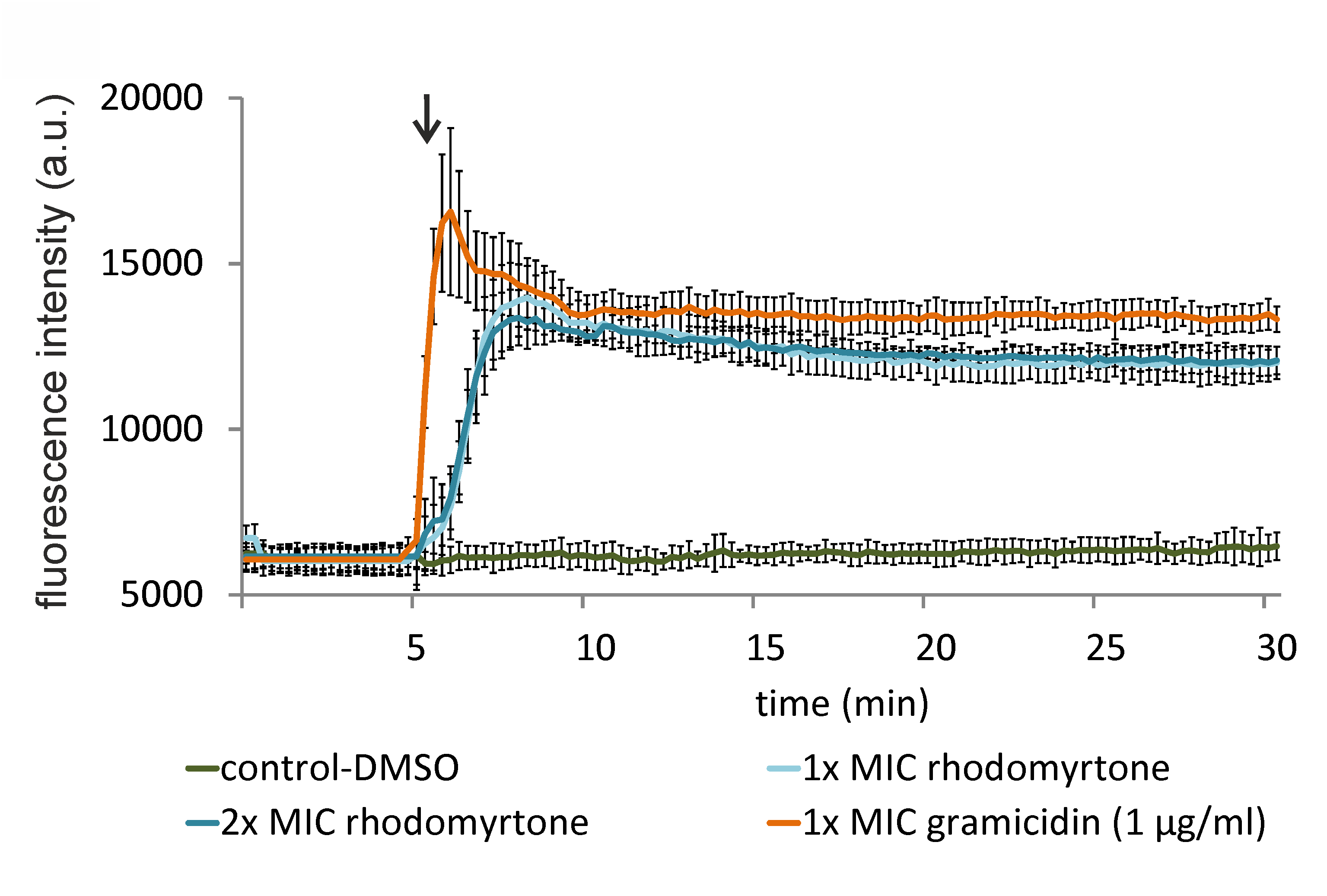

Supplement: S4 Fig — Arrow indicates time point of antibiotic addition. (TIF) [file ppat.1006876.s009.tif]

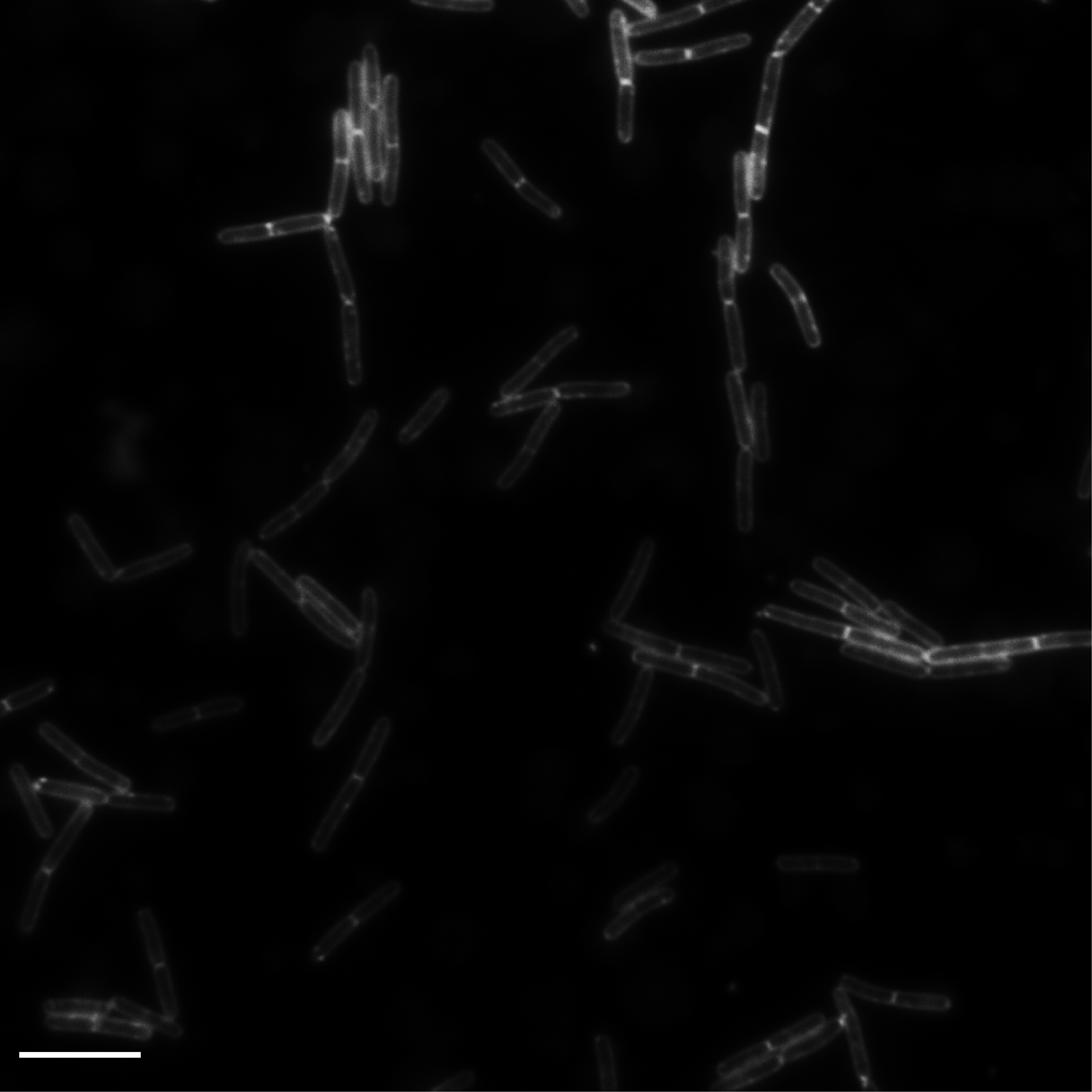

Supplement: S5 Fig — Scale bar 5 μm. (TIF) [file ppat.1006876.s010.tif]

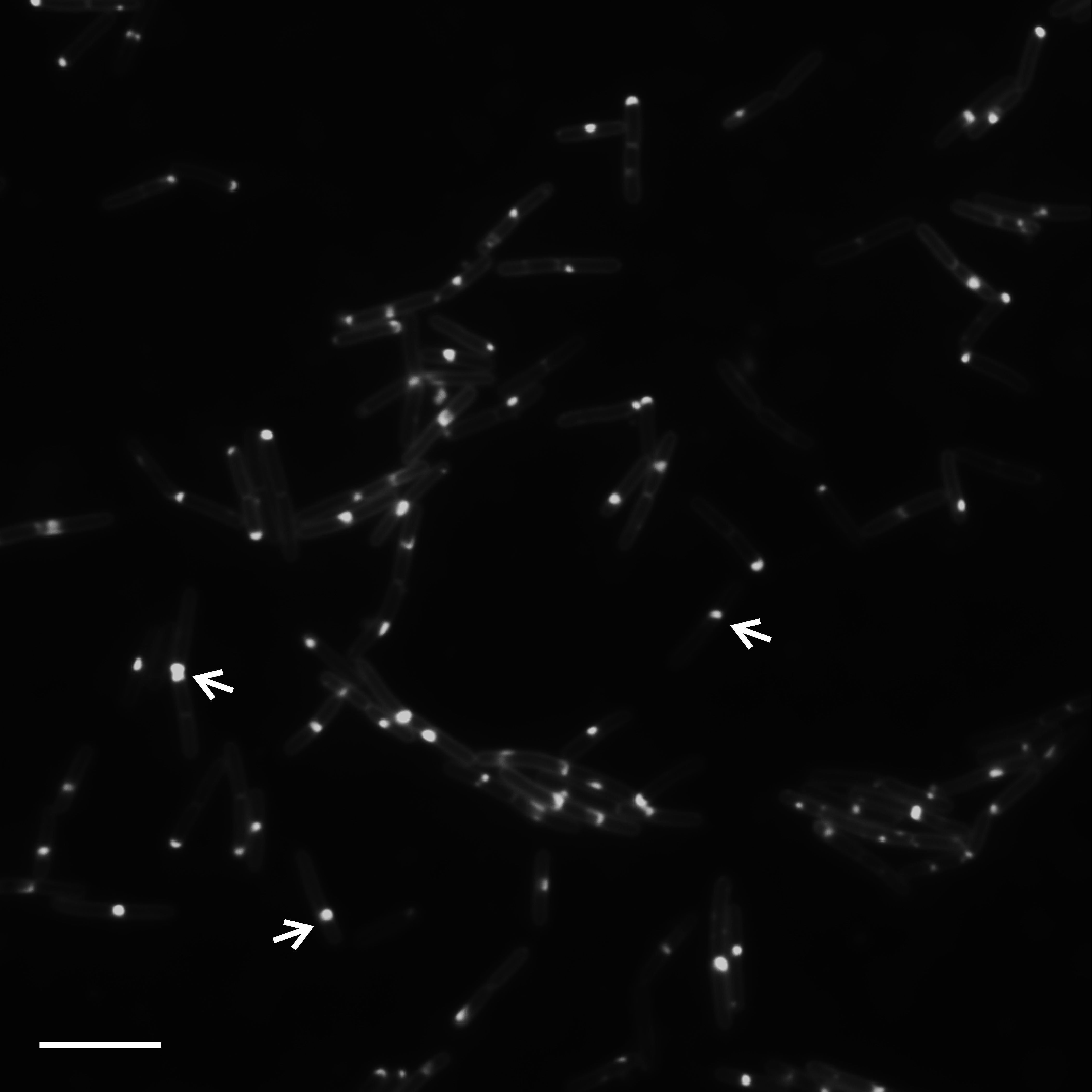

Supplement: S6 Fig — Cell were treated with 1x MIC for 10 min. Arrows indicate some of the FM5-95 patches. Scale bar 5 μm. (TIF) [file ppat.1006876.s011.tif]

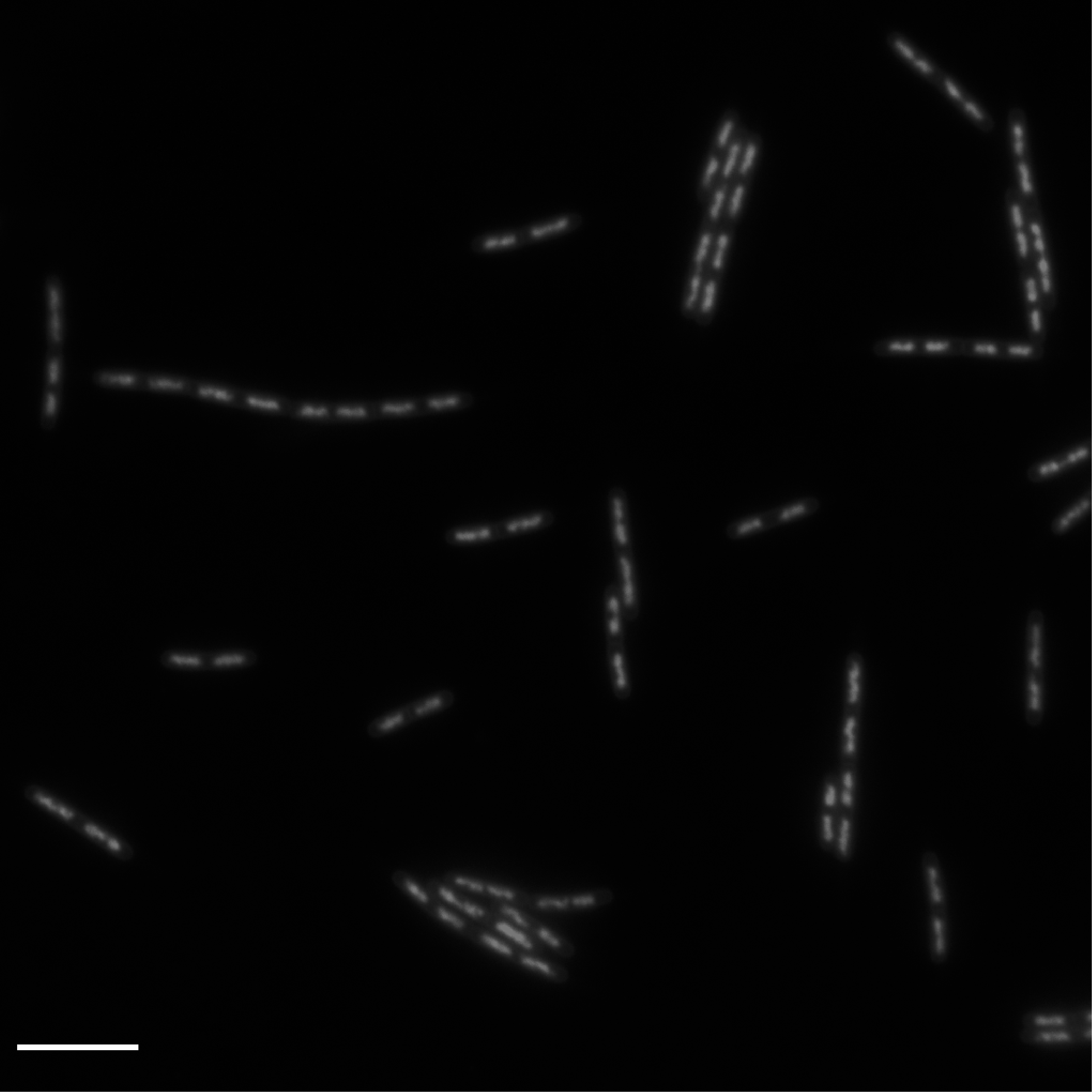

Supplement: S7 Fig — Scale bar 5 μm. (TIF) [file ppat.1006876.s012.tif]

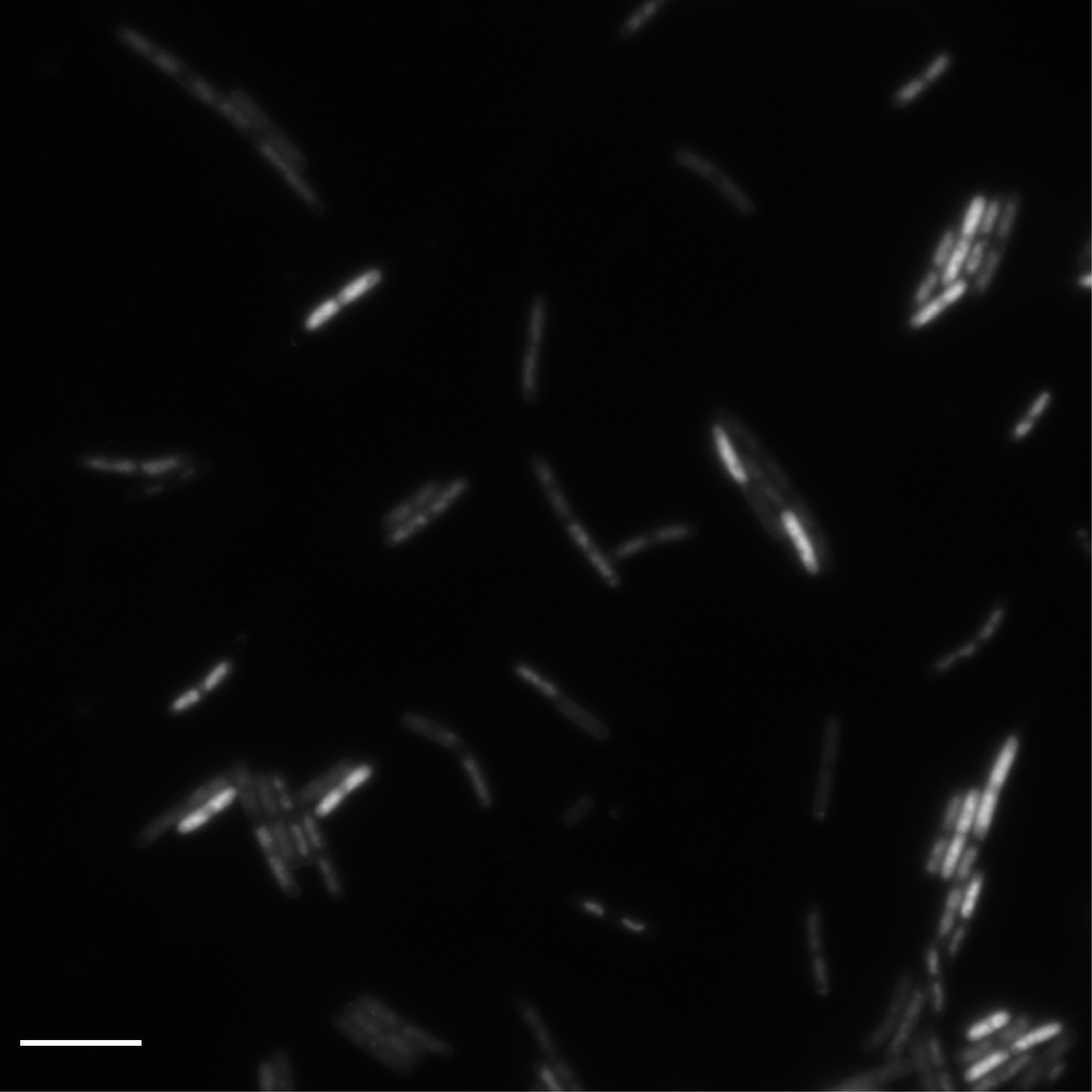

Supplement: S8 Fig — Cells were treated with 1x MIC for 10 min. Note the heterogeneity of the DAPI stain due to increased membrane permeability in severely affected cells. Scale bar 5 μm. (TIF) [file ppat.1006876.s013.tif]

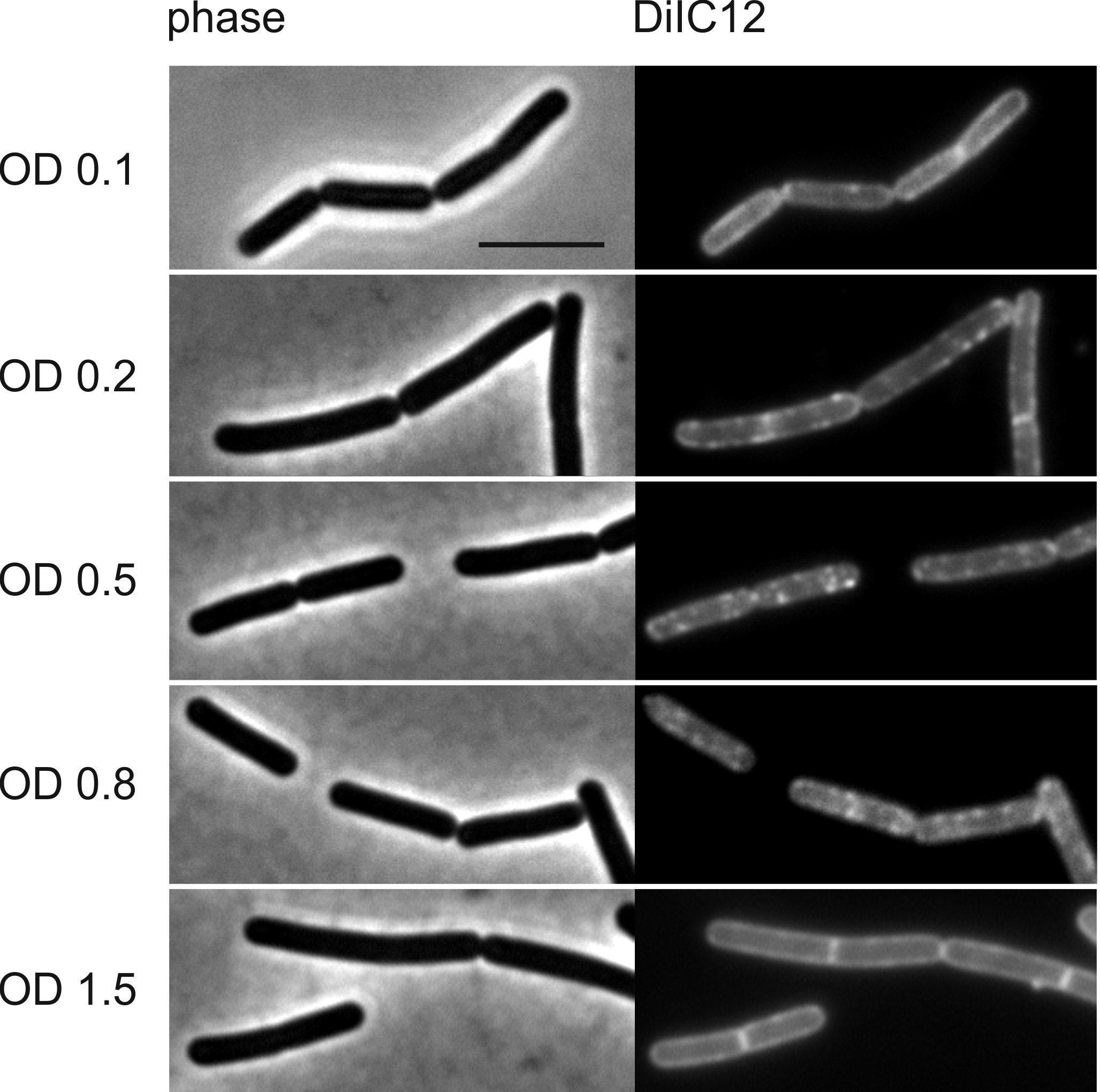

Supplement: S9 Fig — B. subtilis 168 was aerobically grown in LB at 30°C. Discrete RIFS become visible during logarithmic growth and disappear upon entry into stationary phase. (TIF) [file ppat.1006876.s014.tif]

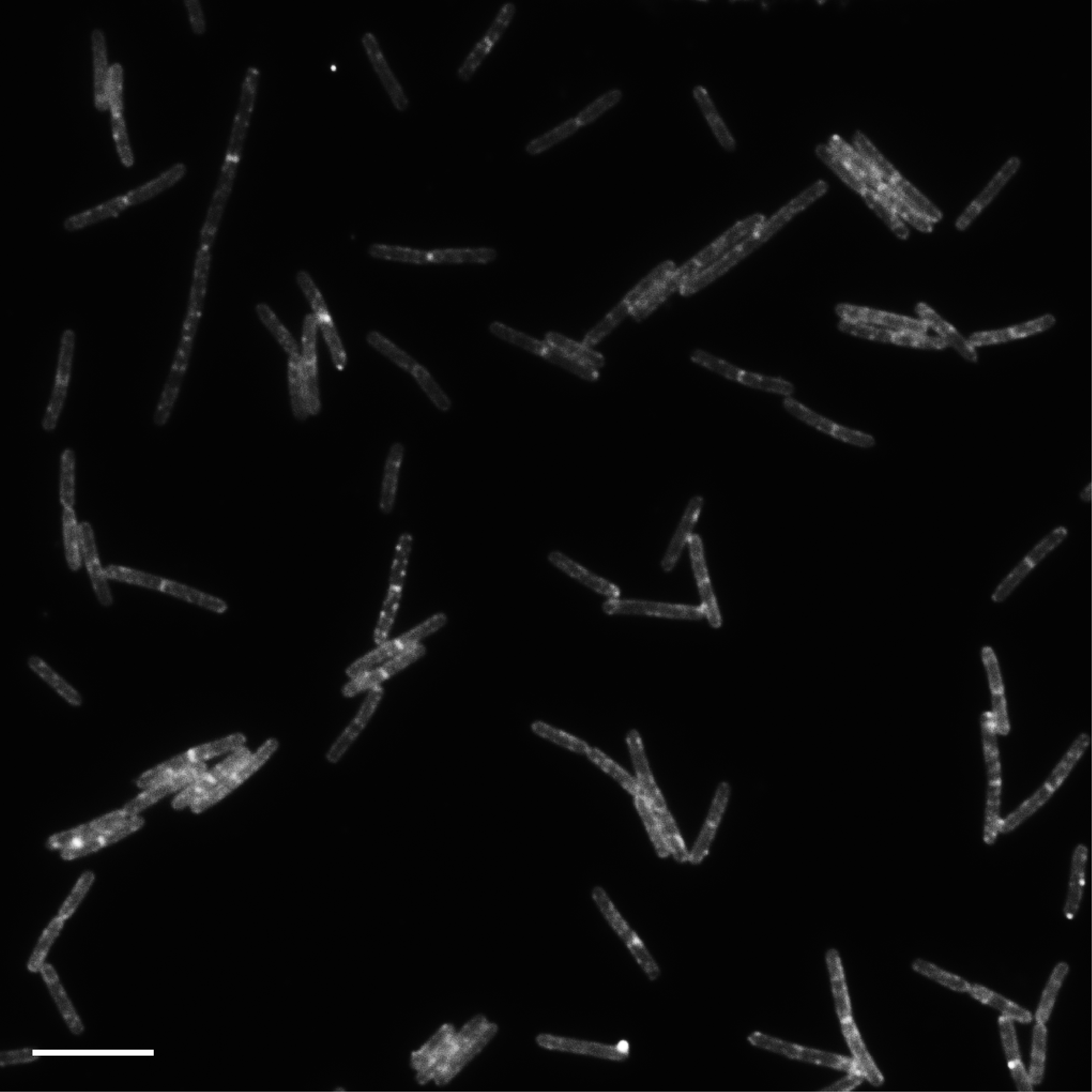

Supplement: S10 Fig — Scale bar 5 μm. (TIF) [file ppat.1006876.s015.tif]

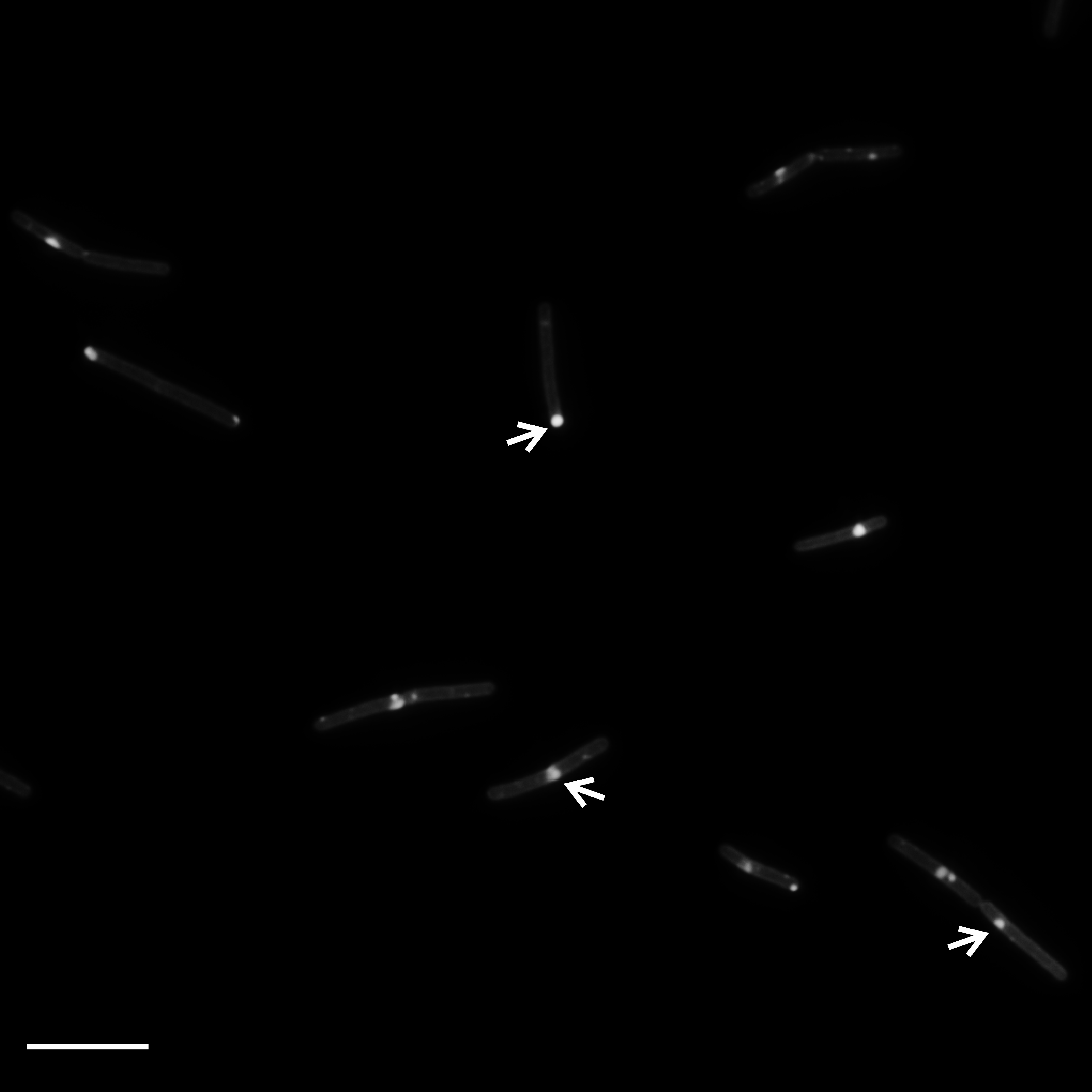

Supplement: S11 Fig — Cells were treated with 1x MIC for 10 min. Arrows indicate some of the DiIC12 patches. Scale bar 5 μm. (TIF) [file ppat.1006876.s016.tif]

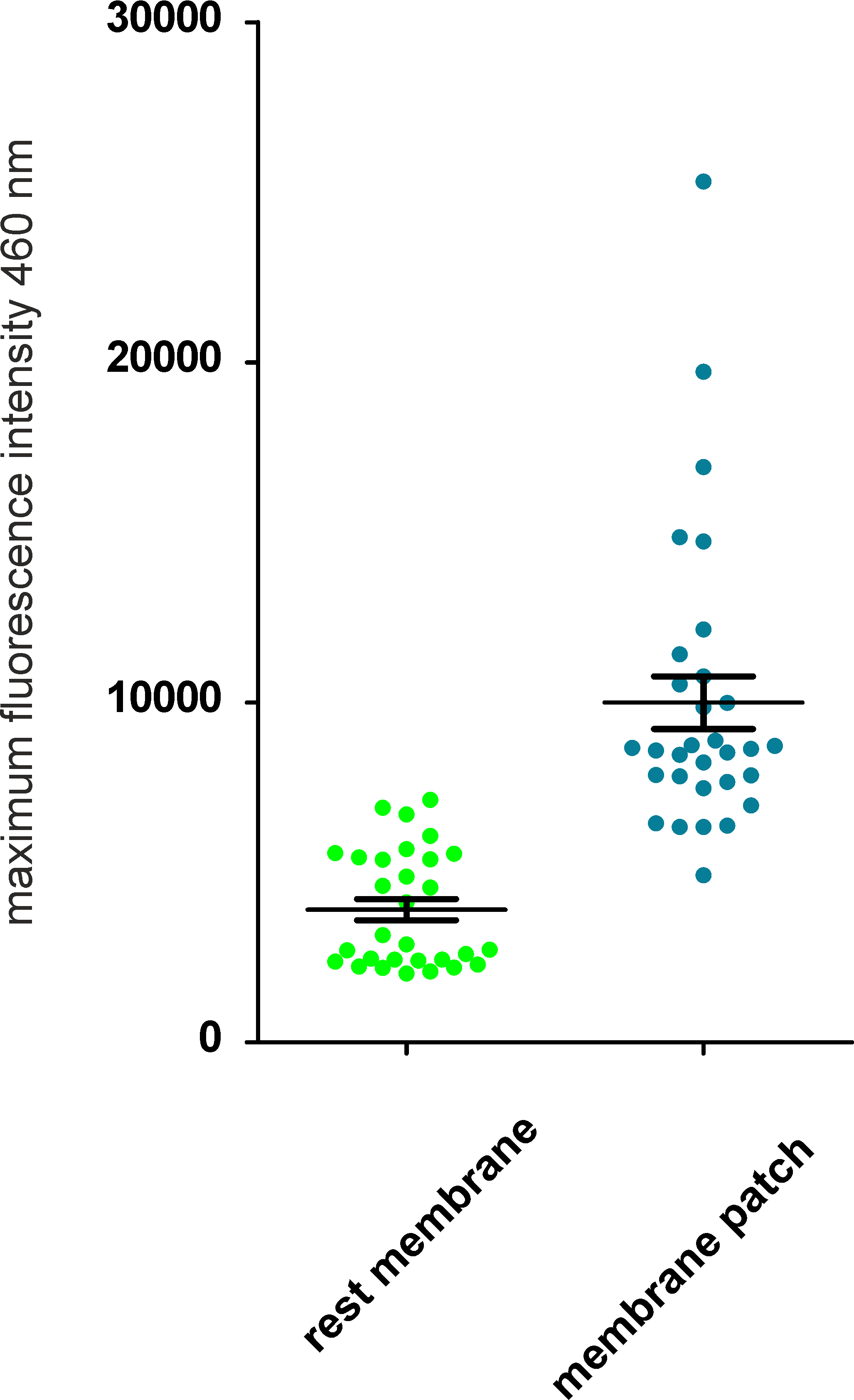

Supplement: S12 Fig — Fluorescence intensity was measured in 460 nm laurdan fluorescence images. Error bars represent standard error of the mean. (TIF) [file ppat.1006876.s017.tif]

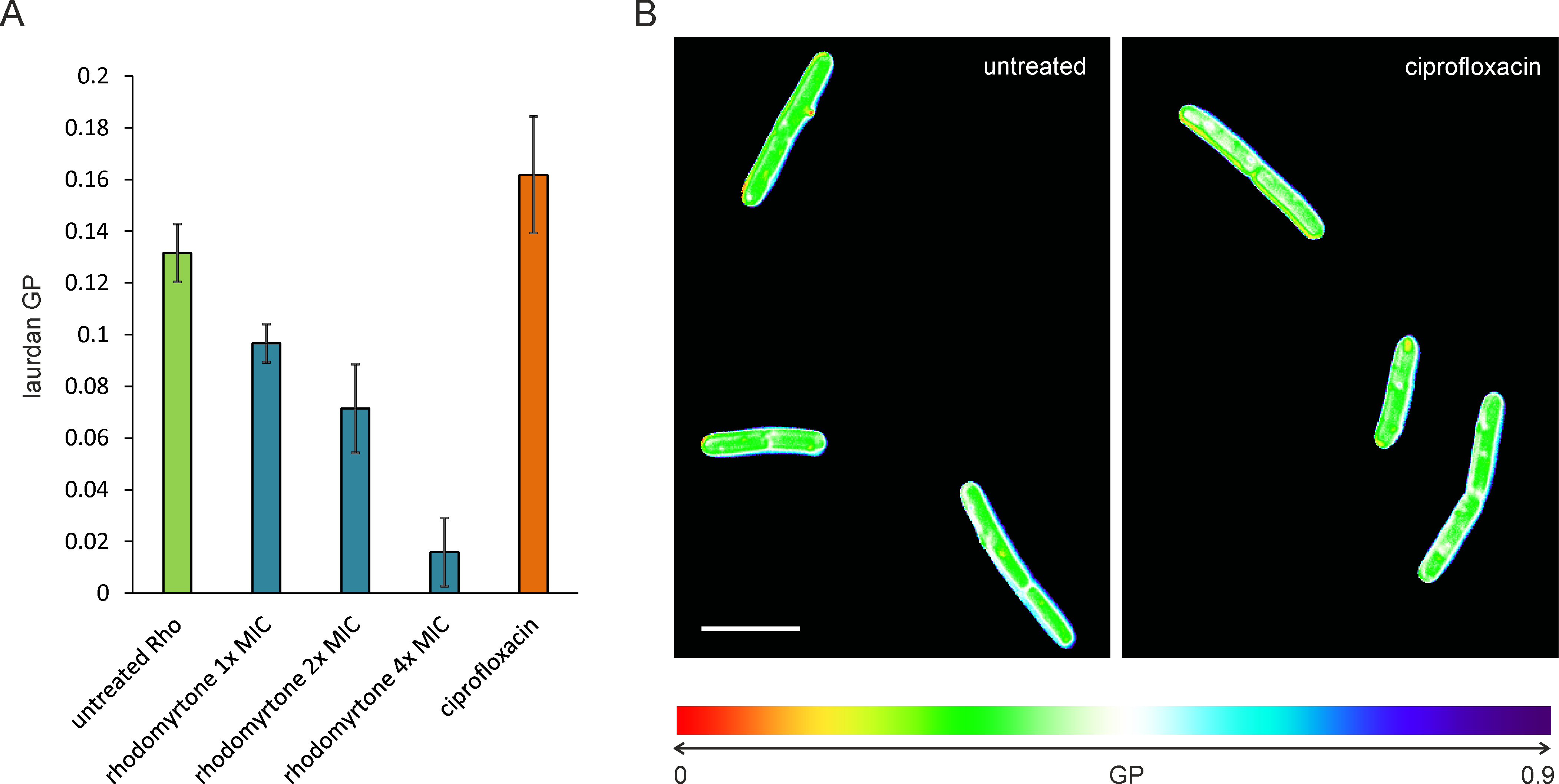

Supplement: S13 Fig — B. subtilis 168 was treated with a bactericidal concentration of ciprofloxacin (1 μg/ml) for 10 min prior to spectroscopic (A) or microscopic (B) fluidity measurements with laurdan. (TIF) [file ppat.1006876.s018.tif]

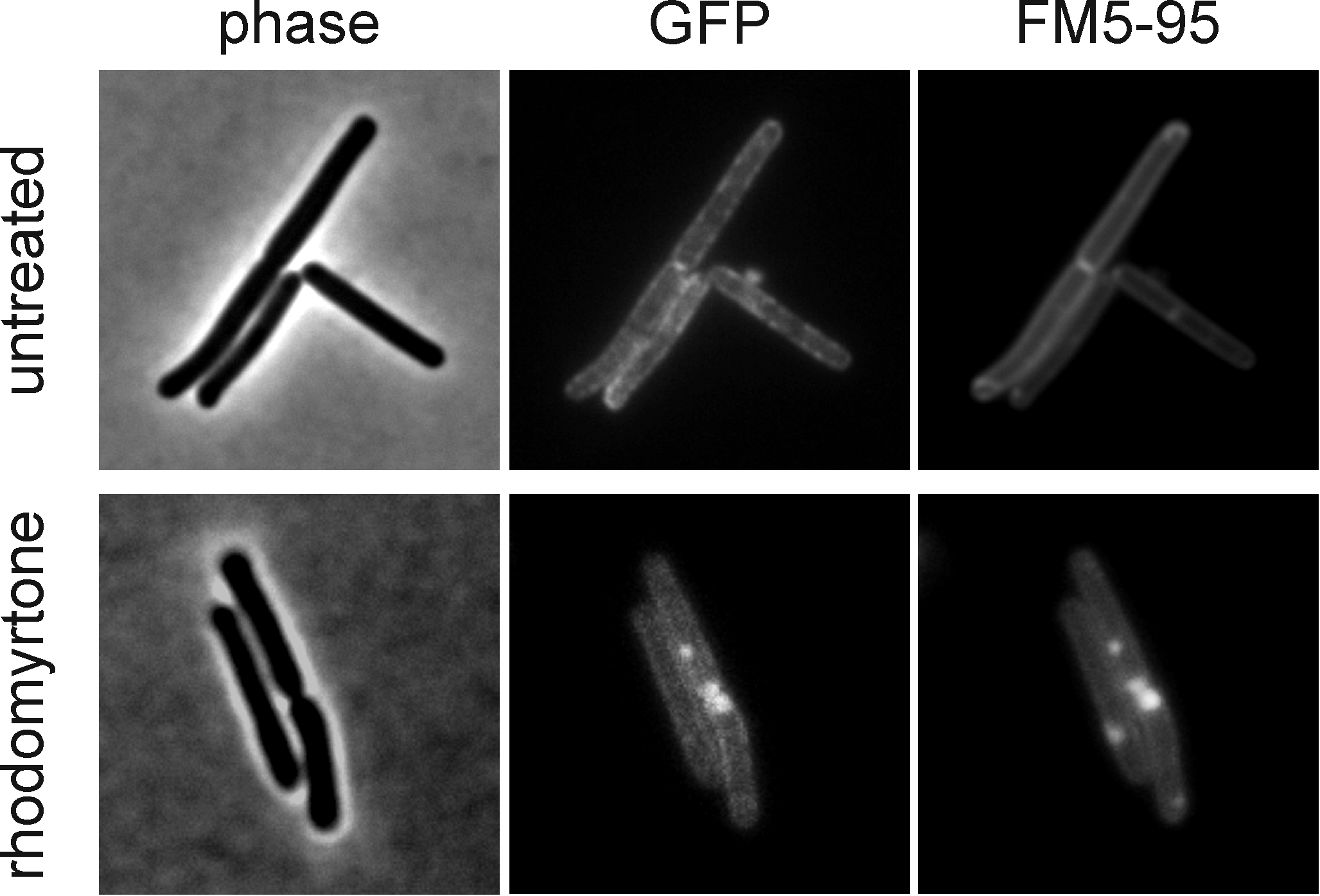

Supplement: S14 Fig — AtpA clearly accumulated in FM5-95-stained membrane domains. (TIF) [file ppat.1006876.s019.tif]

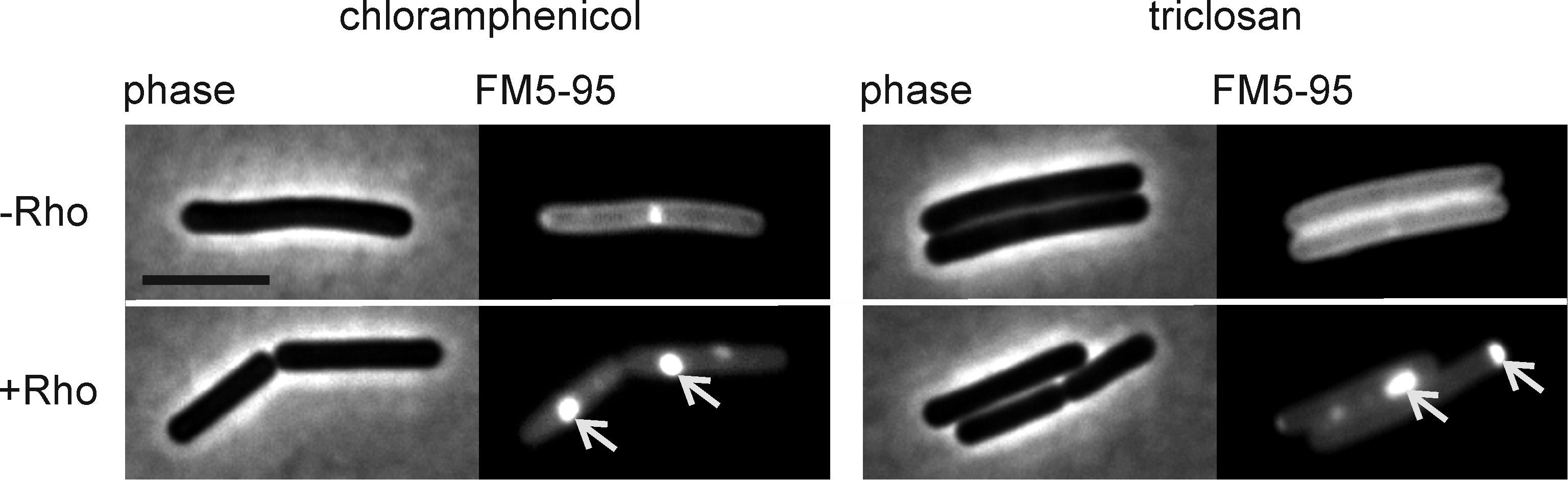

Supplement: S15 Fig — Cells were pre-treated with 100 μg/ml chloramphenicol (left panels) or 2.5 μg/ml triclosan (right panels) for 10 min to inhibit synthesis of proteins and lipids, respectively. Subsequently, rhodomyrtone was added and pictures were taken after additional 10 min. Membranes were stained with FM5-95. (TIF) [file ppat.1006876.s020.tif]

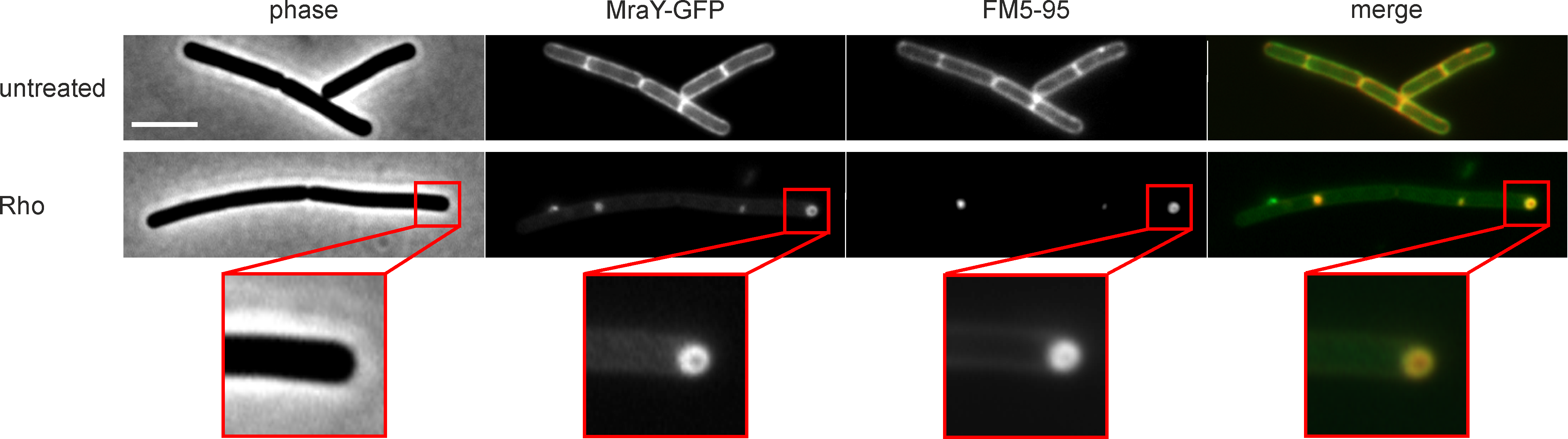

Supplement: S16 Fig — B. subtilis TNVS284 (mraY-gfp) was treated with 1x MIC of rhodomyrtone for 60 min. Membranes were stained with FM5-95. Red boxes indicate MraY trapped in membrane invaginations caused by rhodomyrtone. Scale bar 2 μm. (TIF) [file ppat.1006876.s021.tif]

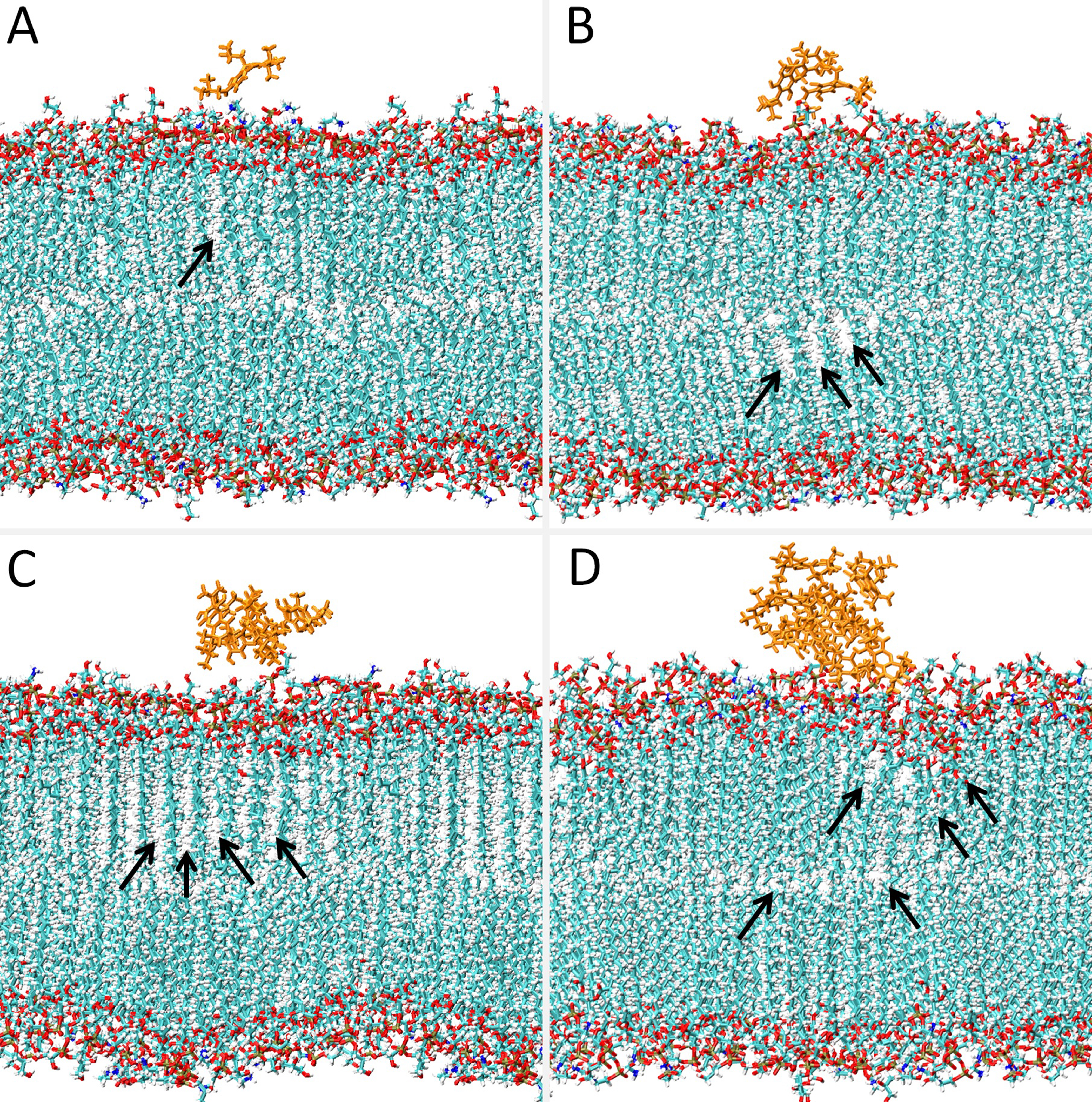

Supplement: S17 Fig — Simulations were run with one (A), two (B), four (C), or eight (D) rhodomyrtone molecules. Snapshots show aggregated rhodomyrtone molecules interacting with the phospholipid head groups. Arrows indicate bilayer disturbance in terms of fatty acyl chain spreading. (TIF) [file ppat.1006876.s022.tif]

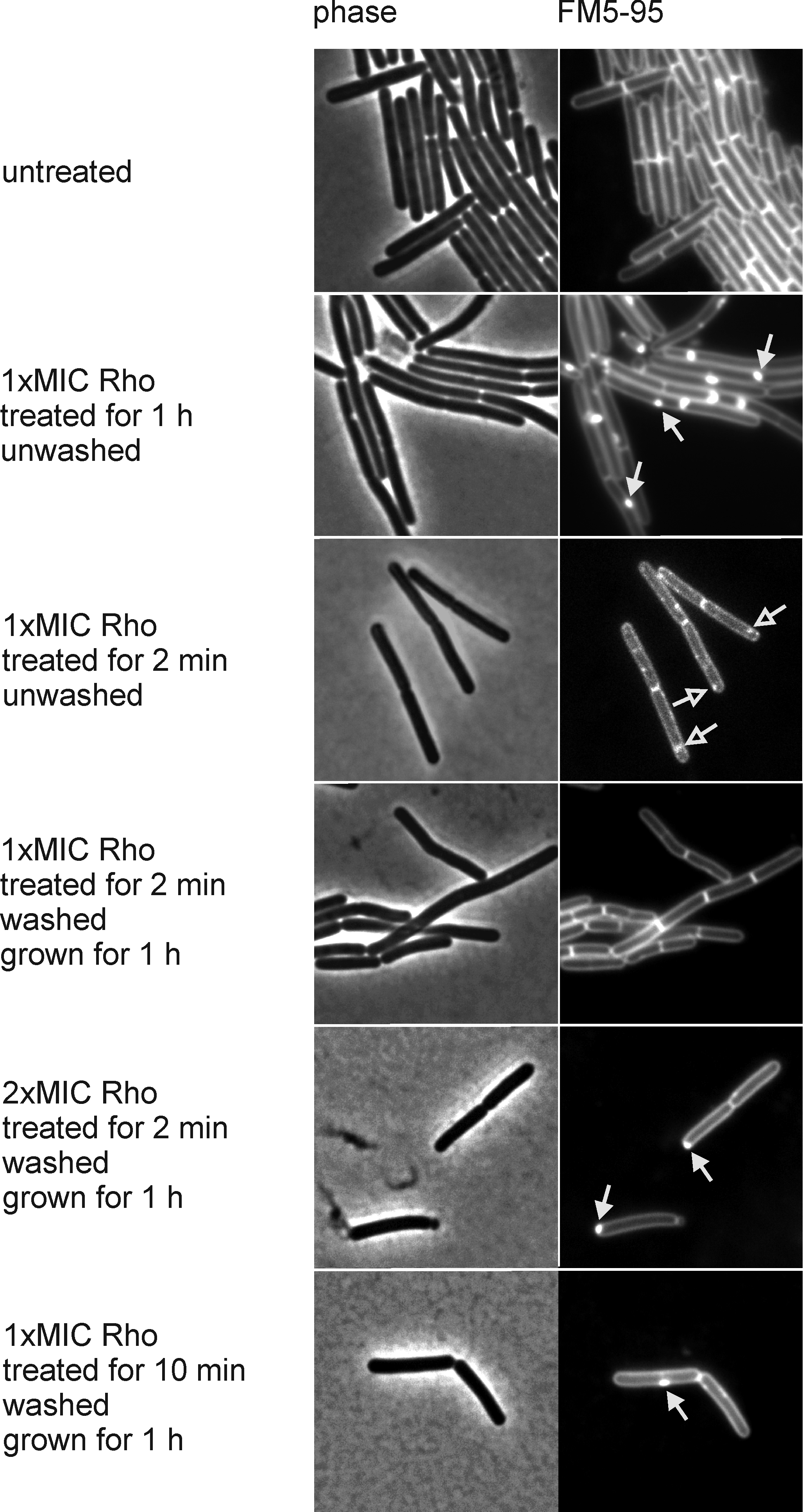

Supplement: S18 Fig — B. subtilis 168 was treated with rhodomyrtone for 2 or 10 min, respectively, and subsequently washed twice with pre-warmed LB medium. Cells were then allowed to grow for 1 h and examined under the microscope. Membranes were stained with FM5-95. Arrows indicate membrane patches caused by rhodomyrtone. Scale bar 2 μm. (TIF) [file ppat.1006876.s023.tif]

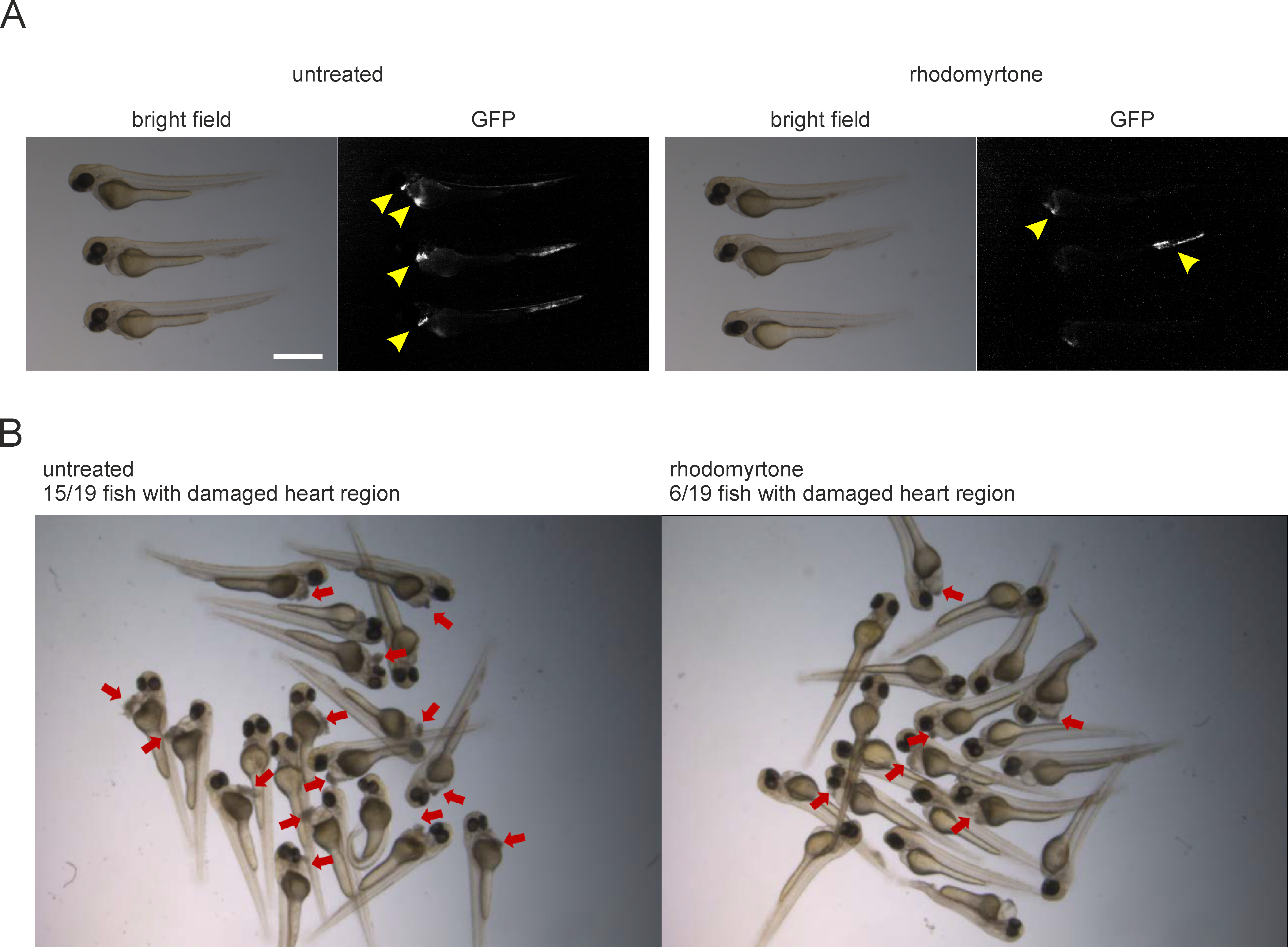

Supplement: S19 Fig — (A) Rhodomyrtone-treated fish consistently showed less green fluorescence caused by infection with a GFP-expressing strain of S. pneumoniae. (B) Rhodomyrtone prevents damage to the heart region caused by S. pneumoniae infection (red arrows). Damage to the heart region was observed in 80% of untreated and 30% of rhodomyrtone-treated fish. One day old zebra fish embryos were injected with 160 CFU of S. pneumoniae JWV500 expressing HlpA-GFP in the tail vein. Fish were treated with two injections (45 and 75 min post infection) of 25 ng rhodomyrtone each. Pictures were taken 18 hours post infection. Experiments were performed in biological triplicates with a minimum of 15 fish per condition in each replicate. (TIF) [file ppat.1006876.s024.tif]

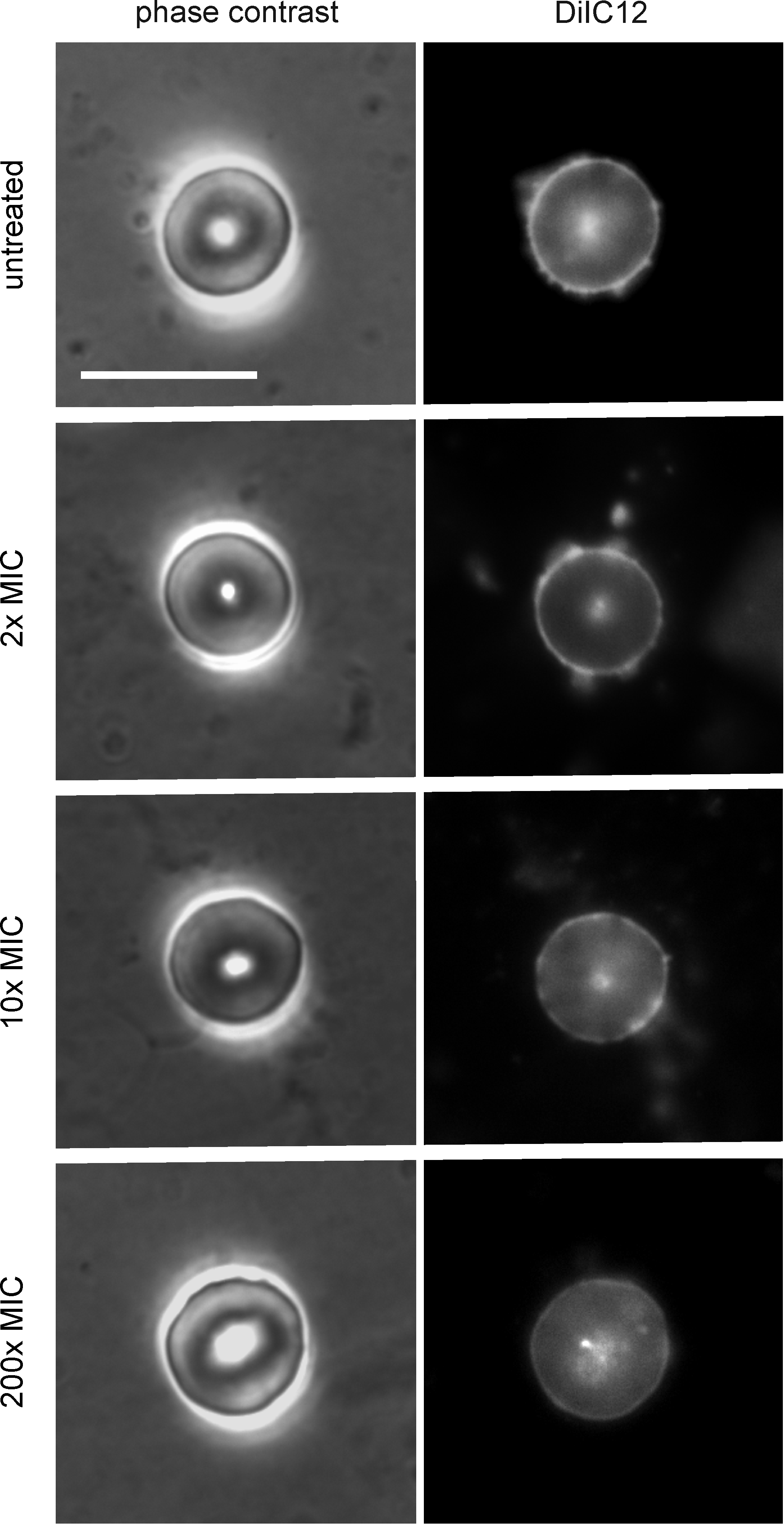

Supplement: S20 Fig — Fresh blood from a healthy donor was stained with 16 μg/ml DiIC12 for 10 min and subsequently treated with rhodomyrtone for 10 additional minutes prior to inspection by fluorescence light microscopy. Scale bar 10 μm. (TIF) [file ppat.1006876.s025.tif]

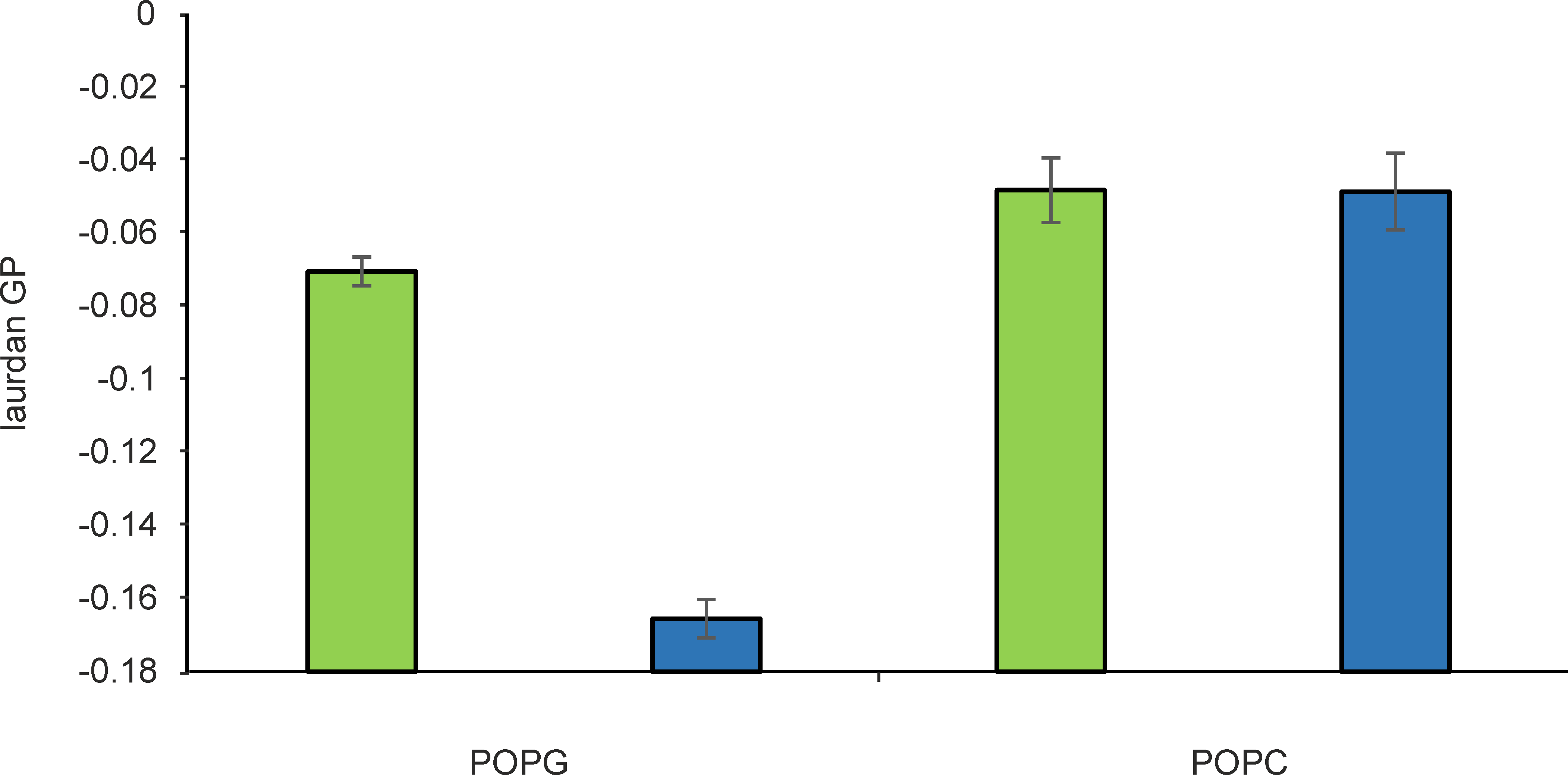

Supplement: S21 Fig — PG is one of the main membrane lipid species in bacteria but only rarely present in mammalian cells, while PC is the major component of mammalian membranes but absent in most bacterial membranes. Green: untreated. Blue: 50 μg/ml rhodomyrtone (compound to lipid ratio 1:7). (TIF) [file ppat.1006876.s026.tif]

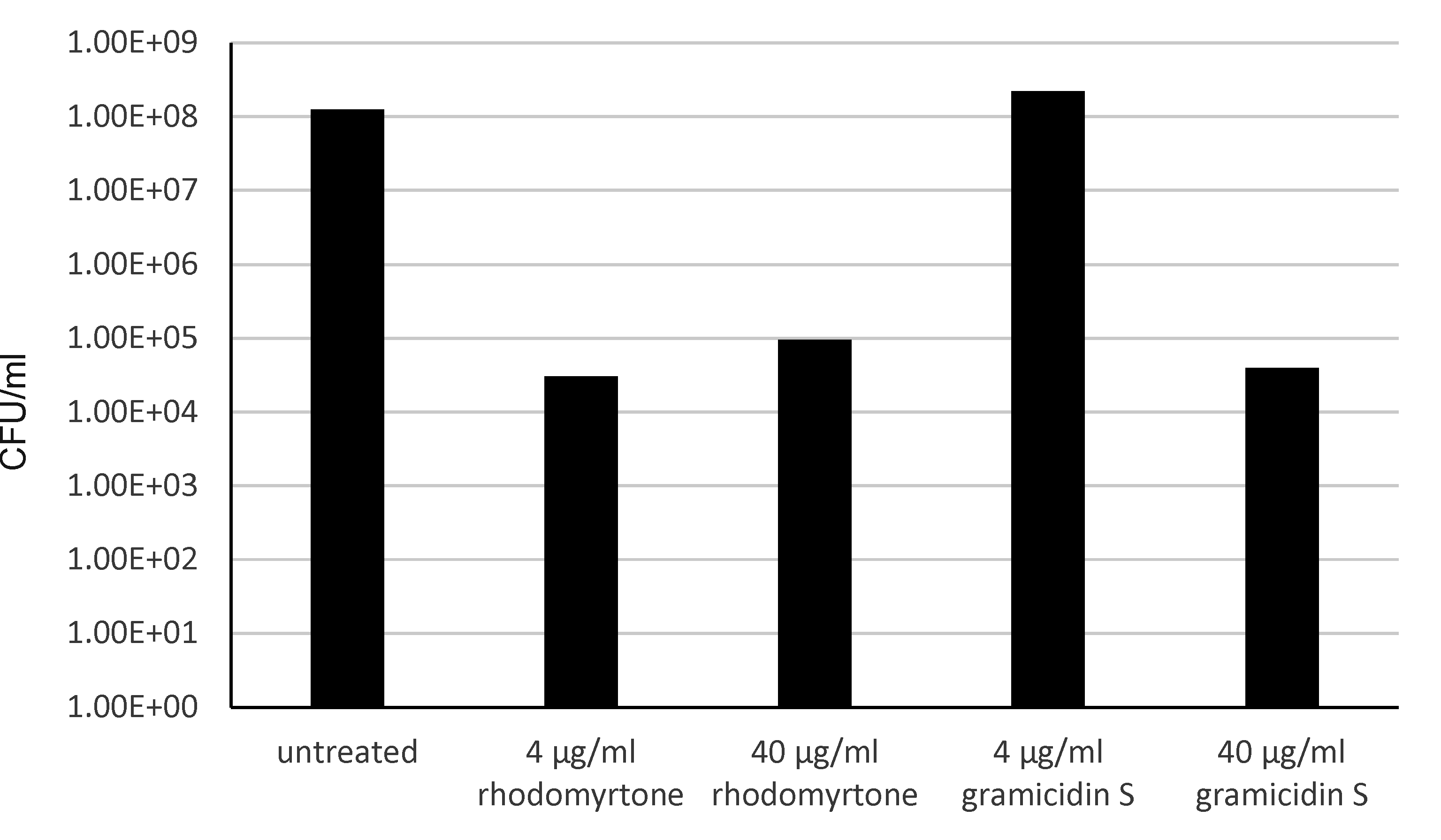

Supplement: S22 Fig — Stationary phase cells were treated with compounds for 9 h prior to plating on non-selective LB agar plates. Gramicidin S, which is known to kill persister cells16, was used as control. (TIF) [file ppat.1006876.s027.tif]

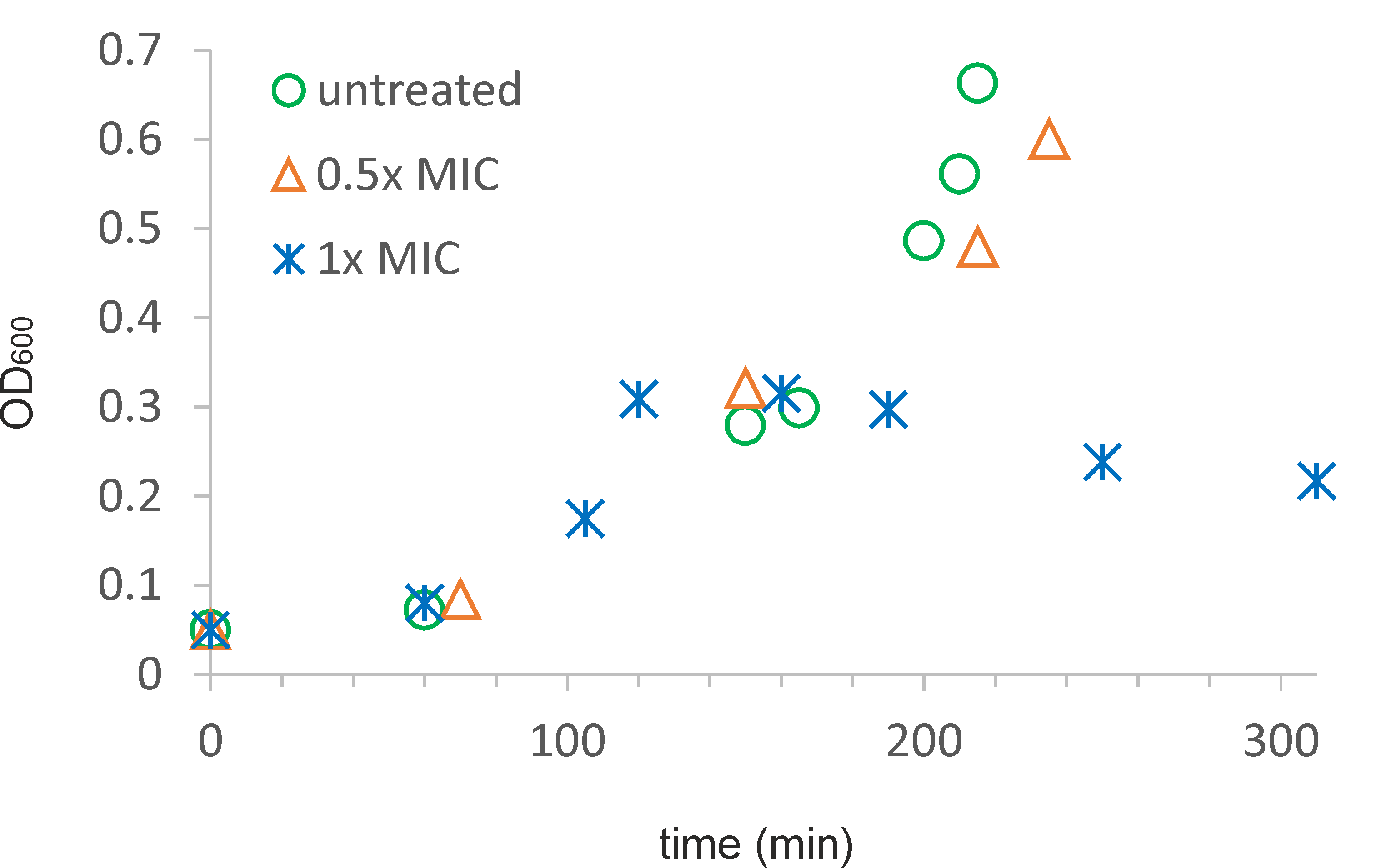

Supplement: S23 Fig — In contrast to growth experiments in 96-well microtiter plates (Fig 3A), 1x MIC led to complete growth inhibition in this experiment (500 ml shaking cultures in 3 L flasks) due to different oxygen supply and subsequent differences in growth rate. Therefore, 0.5x MIC was used for fatty acid analysis. (TIF) [file ppat.1006876.s028.tif]
