# Supplementary material for: The novel antibiotic rhodomyrtone traps membrane proteins in vesicles with increased fluidity
Source: PLoS Pathog. 2018 Feb 16;14(2):e1006876. doi: 10.1371/journal.ppat.1006876 (PMC5833292; doi:10.1371/journal.ppat.1006876)
Supplement: S1 References — (DOCX) [file ppat.1006876.s031.docx]

**S1 References:** References for methods and bacterial strains used in this study.

1. Anagnostopoulos, C. & Spizizen, J. Requirements for transformation in Bacillus subtilis. *J. Bacteriol.* **81,** 741–746 (1960).

2. Lewis, P. J., Thaker, S. D. & Errington, J. Compartmentalization of transcription and translation in Bacillus subtilis. *EMBO J.* **19,** 710–718 (2000).

3. Schirner, K. & Errington, J. The cell wall regulator {sigma}I specifically suppresses the lethal phenotype of mbl mutants in Bacillus subtilis. *J. Bacteriol.* **191,** 1404–1413 (2009).

4. Johnson, A. S., van Horck, S. & Lewis, P. J. Dynamic localization of membrane proteins in Bacillus subtilis. *Microbiology* **150,** 2815–2824 (2004).

5. Jahn, N., Brantl, S. & Strahl, H. Against the mainstream: the membrane-associated type I toxin BsrG from Bacillus subtilis interferes with cell envelope biosynthesis without increasing membrane permeability. *Mol. Microbiol.* **98,** 651–666 (2015).

6. Mueller, A. *et al.* Daptomycin inhibits bacterial cell envelope synthesis by interfering with fluid membrane microdomains. *Proc. Natl. Acad. Sci. U. S. A.* **113,** E7077-7086 (2016).

7. Salzberg, L. I. & Helmann, J. D. Phenotypic and Transcriptomic Characterization of Bacillus subtilis Mutants with Grossly Altered Membrane Composition . *Journal of Bacteriology* **190,** 7797–7807 (2008).

8. Su’etsugu, M. & Errington, J. The replicase sliding clamp dynamically accumulates behind progressing replication forks in Bacillus subtilis cells. *Mol. Cell* **41,** 720–732 (2011).

9. Murray, H. & Koh, A. Multiple regulatory systems coordinate DNA replication with cell growth in Bacillus subtilis. *PLoS Genet.* **10,** e1004731 (2014).

10. Gamba, P., Veening, J. W., Saunders, N. J., Hamoen, L. W. & Daniel, R. A. Two-step assembly dynamics of the Bacillus subtilis divisome. *J. Bacteriol.* **191,** 4186–4194 (2009).

11. Kawai, F. *et al.* Cardiolipin domains in Bacillus subtilis marburg membranes. *J. Bacteriol.* **186,** 1475–1483 (2004).

12. Avery, O. T., Macleod, C. M. & McCarty, M. Studies on the chemical nature of the substane inducing transformation of pneumococcal types: Induction of transformation by a desoxyribonucleic acid fraction isolated from Pneumococcus type III. *J. Exp. Med.* **79,** 137–158 (1944).

13. Kjos, M. *et al.* Bright fluorescent Streptococcus pneumoniae for live-cell imaging of host-pathogen interactions. *J. Bacteriol.* **197,** 807–818 (2015).

14. Kreiswirth, B. N. *et al.* The toxic shock syndrome exotoxin structural gene is not detectably transmitted by a prophage. *Nature* **305,** 709–712 (1983).

15. Strahl, H., Burmann, F. & Hamoen, L. W. The actin homologue MreB organizes the bacterial cell membrane. *Nat. Commun.* **5,** 3442 (2014).

16. Berditsch, M., Lux, H., Babii, O., Afonin, S. & Ulrich, A. S. Therapeutic Potential of Gramicidin S in the Treatment of Root Canal Infections. *Pharmaceuticals (Basel).* **9,** (2016).
